# AAV‐delivered diacylglycerol kinase DGKk achieves long‐term rescue of fragile X syndrome mouse model

**DOI:** 10.15252/emmm.202114649

**Published:** 2022-04-04

**Authors:** Karima Habbas, Oktay Cakil, Boglárka Zámbó, Ricardos Tabet, Fabrice Riet, Doulaye Dembele, Jean‐Louis Mandel, Michaël Hocquemiller, Ralph Laufer, Françoise Piguet, Hervé Moine

**Affiliations:** ^1^ Institut de Génétique et de Biologie Moléculaire et Cellulaire (IGBMC) Département de Médecine Translationelle et Neurogénétique Centre National de la Recherche Scientifique (CNRS UMR7104) Institut National de la Santé et de la Recherche Médicale (INSERM U1258) Université de Strasbourg Illkirch France; ^2^ Institut de Génétique et de Biologie Moléculaire et Cellulaire (IGBMC) PHENOMIN‐ICS, Centre National de la Recherche Scientifique (CNRS UMR7104), Institut National de la Santé et de la Recherche Médicale (INSERM U1258) Université de Strasbourg Illkirch France; ^3^ Lysogene Neuilly‐sur‐Seine France; ^4^ NeuroGenCell, INSERM U1127, Paris Brain Institute (ICM), Sorbonne University, CNRS, AP‐HP University Hospital Pitié‐Salpêtrière Paris France; ^5^ Present address: Pfizer, Inc Cambridge MA USA

**Keywords:** AAV, diacylglycerol kinase, *Fmr1*‐KO, FMRP, Fragile X syndrome, Genetics, Gene Therapy & Genetic Disease, Neuroscience

## Abstract

Fragile X syndrome (FXS) is the most frequent form of familial intellectual disability. FXS results from the lack of the RNA‐binding protein FMRP and is associated with the deregulation of signaling pathways downstream of mGluRI receptors and upstream of mRNA translation. We previously found that diacylglycerol kinase kappa (DGKk), a main mRNA target of FMRP in cortical neurons and a master regulator of lipid signaling, is downregulated in the absence of FMRP in the brain of *Fmr1*‐KO mouse model. Here we show that adeno‐associated viral vector delivery of a modified and FMRP‐independent form of DGKk corrects abnormal cerebral diacylglycerol/phosphatidic acid homeostasis and FXS‐relevant behavioral phenotypes in the *Fmr1*‐KO mouse. Our data suggest that DGKk is an important factor in FXS pathogenesis and provide preclinical proof of concept that its replacement could be a viable therapeutic strategy in FXS.

## Graphical abstract

Brain neuron‐restricted expression of an FMRP‐independent DGKk enzyme via intra‐cerebral adeno‐associated virus (AAV) gene therapy corrects phosphatidic acid deficiency and rescues disease relevant behavioral phenotypic alterations in a mouse model of Fragile X syndrome (FXS).

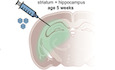

**The paper explained**

**Problem**

Triplet repeat expansion mutations downstream of the FMR1 gene promoter result in methylation of the mutated locus and the silencing of the gene transcription. Lack of FMR1 gene product, FMRP protein, represents the first cause of inherited intellectual disability known as Fragile X syndrome (FXS). There are currently no disease‐modifying treatments for FXS.

**Results**

In this study, we demonstrated that diacylglycerol kinase kappa, a main mRNA target of FMRP involved in brain lipid signaling control, is downregulated in the brain of FXS patients. We showed that DGKk post‐transcriptional control is dependent on FMRP, and its N‐terminal truncation (∆N‐DGKk) alleviates FMRP requirement for its synthesis while preserving its function. We demonstrated that intracerebral injection of AAVRh10‐Synapsin I‐∆N‐DGKk restored DGKk cerebral expression and function associated with reversal of the abnormal behavioral alterations of adolescent *Fmr1*‐null mice.

**Impact**

These results provide support of an important role of DGKk dysfunction in the pathomechanism of the disease and a preclinical proof of concept of DGKk gene therapy at the post‐developmental stage in *Fmr1*‐null mice model of FXS.

## Introduction

Fragile X syndrome (FXS) is a main cause of familial intellectual disability and autistic spectrum disorder (ASD) with a prevalence in general population estimated as 1 in 5,000 males and 1 in 8,000 females (Kaufmann *et al*, 2004; Hagerman *et al*, 2017). FXS is also generally associated with variable behavioral symptoms that can include anxiety, hyperactivity, hypersensitivity, stereotypies, memory deficits, and sleeping problems. FXS results from the loss of the fragile X mental retardation protein (FMRP), an RNA‐binding protein associated with mRNAs and the translation machinery and whose absence in *Fmr1*‐deleted mice (*Fmr1*‐KO) recapitulates FXS‐like phenotypes (The Dutch‐Belgian Fragile X Consortium, 1994; Mientjes *et al*, 2006), with perturbation of neuronal protein synthesis in hippocampus and cortex (Qin *et al*, 2005; Dolen *et al*, 2007). FMRP loss is associated with an overactivation of metabotropic group 1 glutamate receptor (mGluR)‐dependent local mRNA translation (Bear *et al*, 2004), leading to neuronal protein synthesis increase in hippocampus and cortex (Qin *et al*, 2005; Dolen *et al*, 2007), affecting the level of many neuronal proteins including phosphatidylinositol 3‐kinase enhancer (PIKE) (Gross *et al*, 2015), matrix metalloproteinase 9 (MMP9) (Gkogkas *et al*, 2014; Sidhu *et al*, 2014), glycogen synthase kinase 3 (GSK3) (Guo *et al*, 2012), amyloid‐β A4 protein (APP) (Westmark *et al*, 2011; Pasciuto *et al*, 2015), and phosphodiesterase 2A (Maurin *et al*, 2018).

The mGluR signaling overactivation is one well‐established triggering factor of FXS pathomechanism, and we recently showed that mGluR diacylglycerol‐ (DAG) and phosphatidic (PA)‐dependent signaling upstream of local mRNA translation is disrupted by FMRP loss (Tabet *et al*, 2016a). The diacylglycerol kinase kappa (DGKk) transcript was identified as the mRNA species most efficiently bound by FMRP in mouse cortical neurons cultures and with highest *in vitro* binding affinity. DGKk expression was found severely reduced in the brain of *Fmr1*‐KO mouse, and a perturbation of DAG/PA acid homeostasis was observed in *Fmr1*‐KO cortical neurons and in the brain of FXS individuals, suggestive of a decreased DGK activity and altered DAG/PA signaling (Tabet *et al*, 2016a). DGKk knockdown in wild‐type mouse brains recapitulated FXS‐like behaviors, and overexpression of DGKk in *Fmr1*‐KO hippocampal slices rescued their abnormal dendritic spine morphology (Tabet *et al*, 2016a). Overall, DGKk appears to play a key role in dendritic spine morphology and function and in the determination of FXS‐like behaviors. DGKk loss of function was proposed to be at the origin of various abnormal forms of synaptic signaling in FXS by causing altered DAG/PA signaling (Tabet *et al*, 2016b). Thus, being a most proximal downstream mediator of FMRP action, DGKk could represent an interesting actionable therapeutic target.

Here we show that DGKk expression is lost in FXS patients’ postmortem brains similarly to what was shown in *Fmr1*‐KO mice (Tabet *et al*, 2016a). We show that the 5′ proximal region of DGKk mRNA encompassing 5′UTR and N‐terminal region is important for its positive translational control by FMRP and the deletion of this region renders the expression of the truncated protein (∆N‐DGKk) independent of FMRP, suggesting that FMRP alleviates a translation blockade. ∆N‐DGKk conserves its ability to trigger DAG/PA signaling like full‐length protein and to modulate protein synthesis rate and eIF4E phosphorylation in neurons. Moreover, ∆N‐DGKk expression in mouse brain using adeno‐associated virus Rh10 (AAV Rh10) has no overt side effect in wild‐type mouse while it is able to correct brain lipid profile dysregulations and to achieve long‐term behavioral rescue (over 8 weeks after injection) of *Fmr1*‐KO mouse, providing a first proof of principle of *DGKk* gene therapy in a mouse model of FXS.

## Results

### DGKk mRNA translation requires FMRP and DGKk N‐terminal truncation alleviates FMRP control

**Figure 1:**
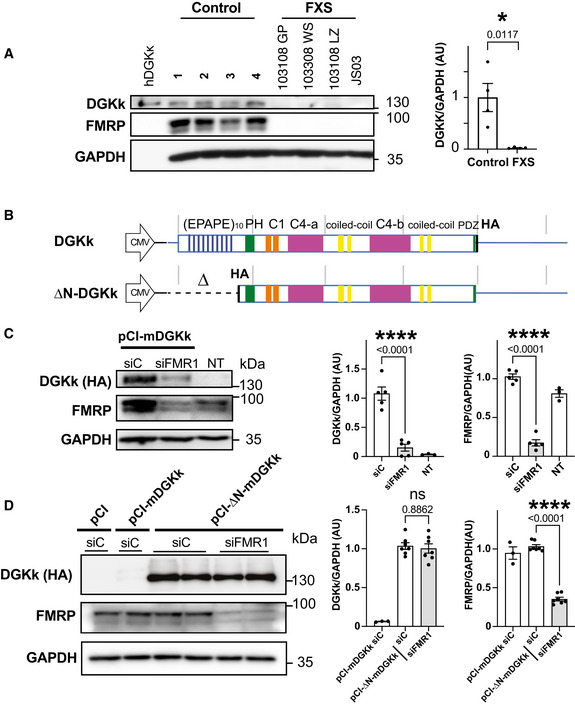
DGKk expression is altered in FXS, and FMRP is required for translation control via its N‐terminal region AWestern blot analysis of lysates from cerebellum of control and FXS patients “FXS” (*n* = 4). Extract of pCI‐hDGKk‐transfected Hela cells (hDGKk) was used as antibody specificity control and size marker. Representative images of immunoblots probed with antibodies against the indicated proteins are shown. GAPDH was the loading control. Quantification of Western blots is shown on right. Protein amounts of DGKk are normalized to GAPDH and presented as fold change relative to control. Bars and error bars represent means ± SEM. *P* value was calculated using an unpaired two‐tailed *t*‐test. *P < 0.05.BSchematic map of DGKk constructs used for the transfection experiments and subsequent vector preparations. The different domains of the protein are indicated and represented at scale (repeated EPAPE, Pleckstrin Homology PH domain, phorbol ester/diacyl glycerol binding C1 domain, catalytic split C4 a and b domains, putative PDZ binding motive, HA‐tag, gray bars interval 1kB), 5′ and 3′ UTR regions are represented with blue line, 3′UTR not at scale (3.8 kB).C, DImmunoblots and quantification of lysates from Cos‐1 cells transfected with plasmid pCI‐mDGKk‐HA or non‐transfected (NT) and pre‐transfected 24 h before with siRNA control (siC) or against FMRP (siFMR1). GAPDH was used as a loading control. For quantification, the DGKk and FMRP signals were normalized against GAPDH signal and presented relative to the signal for siC‐treated cells (C). Immunoblots and quantification of lysates from Cos‐1 cells transfected with pCI (empty plasmid), pCI‐HA‐∆N‐DGKk, or pCI‐mDGKk‐HA, and pre‐treated with siRNA control (siC) or against FMRP (siFMR1) (D). Quantifications as in C. Each point represents data from an individual culture, and all values are shown as mean ± SEM. *P* values were calculated using one‐way ANOVA with Tukey's multiple comparison test. ****P < 0.0001; ns, *P* > 0.05. Source data are available online for this figure.

We previously showed that the expression of diacylglycerol kinase kappa (DGKk) is altered in the brain of *Fmr1*‐KO mouse (Tabet *et al*, 2016a) in agreement with the fact that DGKk mRNA was identified as the most abundant mRNA species associated with FMRP by crosslinking immunoprecipitation in dissociated cortical neuron cultures, and with the highest *in vitro* binding affinity. Although availability of quality protein extracts from human brain is very limited, we could show that DGKk expression is highly reduced in FXS postmortem cerebellum extracts compared to unaffected controls (Fig 1A) similarly to what was observed in mouse. These data suggest that DGKk requires FMRP for its proper expression.

**Figure EV1:**
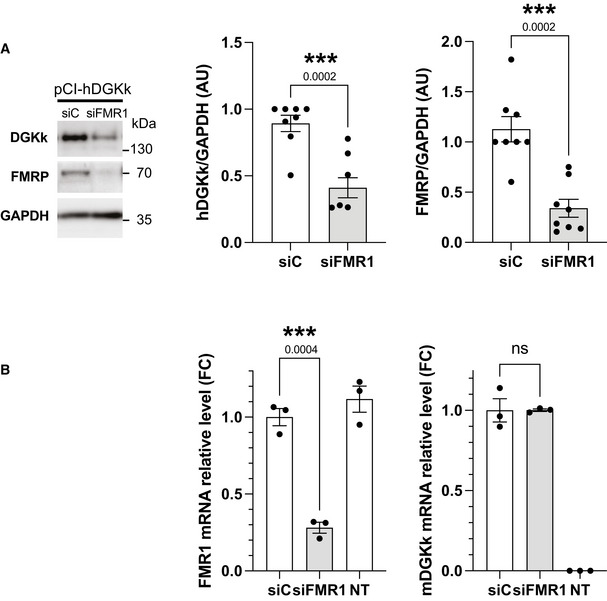
Influence of FMRP protein on human DGKk protein expression and on mouse DGKk mRNA level Immunoblots and quantification of lysates from Hela cells transfected with plasmid pCI‐hDGKk encoding human DGKk and pre‐transfected 24 h before with siRNA control (siC) or against FMRP (siFMR1). GAPDH was used as a loading control. For quantification, DGKk and FMRP signals were normalized against GAPDH signal and presented relative to the signal for siC‐treated cells. Each point represents data from an individual culture, and all values are shown as mean ± SEM ***P < 0.001, calculated by unpaired Student *t*‐test.Quantification of mouse DGKk mRNA by qRT‐PCR in RNA extracts of Cos‐1 cells transfected with plasmid pCI‐mDGKκ‐HA and pre‐transfected with siRNA control (siC) or siRNA against FMRP (siFMR1) or mock transfected (NT). Each point represents data from an individual culture, and all values are means of fold change ± SEM, determined using ∆∆Ct method with Actb as normalizer, *n* = 3 biological replicates. *P* values were calculated using one‐way ANOVA with Tukey's multiple comparison test. ***P < 0.001; ns, *P* > 0.05. Source data are available online for this figure.

DGKk is mostly expressed in neurons and is almost absent in non‐neuronal cells (Tabet *et al*, 2016a). We then tested if it is possible to recapitulate FMRP control in a non‐neuronal cell system. We analyzed the influence of FMRP on the expression of HA‐tagged mouse and human DGKk (Figs 1B and EV1A) borne on plasmids transfected into two different cell lines, Cos‐1 and Hela. Knockdown of endogenous FMRP with siRNA severely reduced the expression level of mouse DGKk (mDGKk) compared to control siRNA treated Cos‐1 cells (Fig 1C) indicating a strong FMRP requirement for DGKk expression. Human DGKk (hDGKk) level is also affected by FMRP knockdown, indicating that FMRP control is conserved between mouse and human (Fig EV1A). While its protein level is severely reduced, the level of mDGKk mRNA is not influenced by the lack of FMRP (Fig EV1B), supporting a control mechanism at the post‐transcriptional level. This is in agreement with our previous data in mouse brain indicating that the loss of FMRP does not affect DGKk mRNA transcript level but alters its association with polyribosomes (Tabet *et al*, 2016a).

Noticeably, DGKk is the only DGK isozyme whose transcript strongly interacts with FMRP (Tabet *et al*, 2016a) and bears a long N‐terminal extension constituted of unique proline‐rich and EPAP repeated motives (Imai *et al*, 2005) (Fig 1B). The N‐terminal part of the protein might play a critical role in DGKk expression considering the repetitive nature of the EPAP domain at the beginning of the coding sequence. Thus, we generated an mDGKk construct lacking the 5′UTR and the first 696 bases following the start codon, encompassing the EPAP domain (∆N‐DGKk), for expression assessment in cells. ∆N‐DGKk mutation led to a strong increase of its level of protein expression, about ten‐fold higher than full‐length protein (Fig 1D). FMRP reduction did not affect ∆N‐DGKk (Fig 1D), suggesting that the 5′UTR and/or N‐terminal domain of DGKk is required for FMRP control and that ∆N‐DGKk expression does no longer depend on the presence of FMRP. Removal of N‐terminal region of DGKk does not impact DGKk activity *in vitro* (Imai *et al*, 2005), and we previously showed that transfection of ∆N‐DGKk in CA1 neurons of 13 DIV hippocampal organotypic slice cultures corrected the abnormal morphology and dynamics of their dendritic spines *in vitro* (Tabet *et al*, 2016). Therefore, ∆N‐DGKk could represent a potential therapeutic candidate for FXS gene therapy by bypassing the need for FMRP.

### ∆N‐DGKk counteracts FXS‐like molecular defects

**Figure 2:**
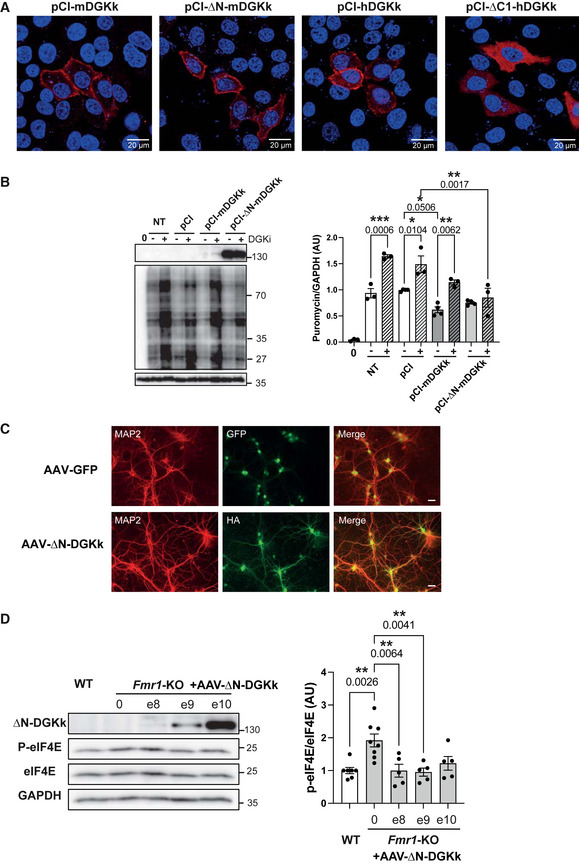
∆N‐DGKk localizes and impacts cellular signaling similarly to full‐length protein HeLa cells transfected with indicated plasmids expressing mouse (m) or human (h) DGKk were analyzed by immunofluorescence confocal microscopy for HA tag (red) and Dapi staining (blue). ∆C1‐hDGKk construct bears deletion of phorbol ester C1 domain (328–449).Immunoblots and quantification of lysates from Cos‐1 cells transfected with plasmid pCI‐HA‐∆N‐DGKk, untransfected (NT), or plasmid pCI control, with the indicated amount of plasmid (µg) and after 24 h, incubated with puromycin (20 µg/ml, 30 min) to measure rates of protein synthesis. GAPDH was used as a loading control. 0 indicates no puromycin treatment, −/+ indicates treatment without or with DGK inhibitor (DGKi) 3 µM R59022 and 0.2 µM R59949 at 6 µM, 15 min. Densitogram of puromycin incorporation is presented as change relative to mock transfected conditions. Each point represents data from an individual culture, and all values are shown as mean ± SEM. *P* values were calculated using one‐way ANOVA with Tukey’s multiple comparison test. *P < 0.05; **P < 0.01; ***P < 0.001.Representative immunofluorescence staining of cortical neuron cultures transduced at 8 DIV (days *in vitro*) with 10e9 VG/ml of AAVRh10‐GFP or AAVRh10‐∆N‐DGKk and assessed after 5 days using anti‐MAP2 and anti‐HA for ∆N‐DGKk or direct 488 nm excitation for GFP. DAPI was used to visualize nuclei on merged images. Scale bar, 40 µm.Representative immunoblots of lysates from WT and *Fmr1*‐KO cortical neurons transduced with AAVRh10‐∆N‐DGKk (AAV ∆N‐DGKk), at the indicated titers (VG/ml culture volume) and quantification of phosphorylation and total levels of eIF4E. GAPDH was used as a loading control. For quantification, the phospho‐protein signal was normalized first against total protein signal and is presented relative to the signal for WT culture. Each point represents data from an individual culture, and all values are shown as mean ± SEM. *P* values were calculated using one‐way ANOVA with Tukey's multiple comparison test. **P < 0.01. Source data are available online for this figure.

**Figure EV2:**
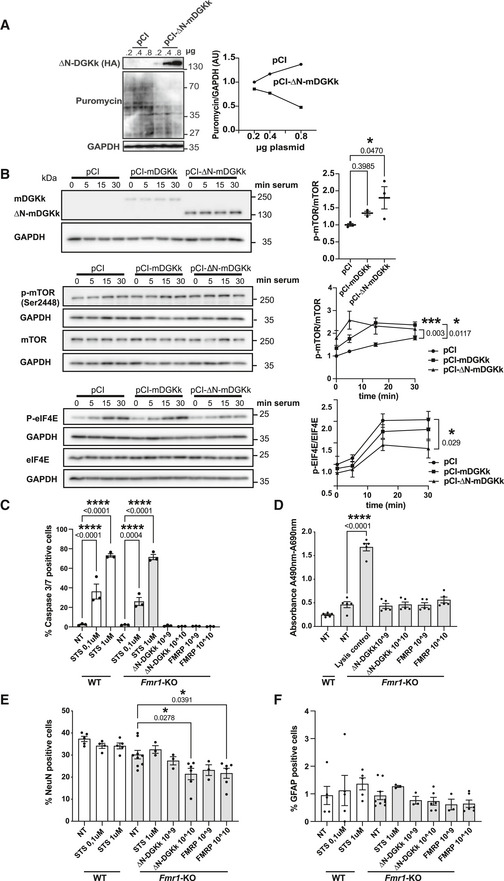
∆N‐DGKk expression modulates mTOR and EIF4E signaling without significant toxicity AImmunoblots and quantification of lysates from Cos‐1 cells transfected with plasmid pCI control or pCI‐HA‐∆N‐DGKk, with the indicated amount of plasmid (µg) and after 24 h incubated with puromycin (20 µg/ml for 30 min) to measure basal rates of protein synthesis. GAPDH was used as a loading control. Densitogram of puromycin incorporation is presented as change relative to pCI (0.2 µg) condition.BCells transfected with 0.5 μg pCI, pCI‐HA‐FL‐mDGKk, or pCI‐HA‐∆N‐mDGKk plasmids were serum starved for 24 h and then incubated with 10% FCS for the indicated time period. Cells were then collected and immunoblotted with HA (DGKk), p‐mTOR, mTOR, p‐EIF4E, or EIF4E antibodies. Blots were normalized for GAPDH and then phospho/non‐phospho ratio was calculated. Upper panel: mDGKk expression. Middle panel: m‐TOR phosphorylation at Ser‐2448. Bottom panel: p‐eIF4E phosphorylation at Ser‐209. Significance was determined by 2‐way ANOVA compared to pCI control (*n* = 3).C–FQuantification of caspase 3/7 activity (C), release of lactate dehydrogenase (LDH) (D), percentage of NeunN (E), and GFAP positive cells (F), in WT and *Fmr1*‐KO cortical neurons untreated (NT) or transduced at 8 DIV for 8 days with indicated titers of AAV (viral genome copies) AAVRh10‐∆N‐DGKk or AAVRh10‐FMRP by immunofluorescence high‐throughput cell imaging. Positive control wells were treated with apoptotic inducer staurosporine (STS) at 0.1 and 1 µM for 6 h. Data information: Data are mean ± SEM of individual cultures and analyzed using one‐way ANOVA and Tukey’s multiple comparisons test. *P < 0.05, ****P < 0.0001. Source data are available online for this figure.

DGKk deregulation has been proposed to play a key role in FXS pathomechanism by altering DAG/PA signaling and by leading to an excess of DAG and lack of PA potentially responsible of excessive protein synthesis, a major molecular hallmark of FMRP defects (Tabet *et al*, 2016a, 2016b). To test the potential rescuing activity of ∆N‐DGKk and compare it with that of full‐length protein, we first analyzed its intracellular localization. ∆N‐DGKk mostly localizes at the plasma membrane, like full‐length protein or its human ortholog as previously shown (Imai *et al*, 2005), and unlike ∆C1‐hDGKk where DAG‐binding domain deletion prevents its localization to the plasma membrane (Fig 2A). We then analyzed the ability of ∆N‐DGKk to modulate protein synthesis rate. Expression of ∆N‐DGKk in Cos‐1 cells reduced in a concentration‐dependent manner the protein synthesis rate (Fig EV2A), and this effect was counteracted by pretreatment of cells with DGK‐specific inhibitors R59022 and R59949 (Jiang *et al*, 2000) (Fig 2B). Compared to full‐length DGKk, cells expressing ∆N‐DGKk showed lower apparent reduction of protein synthesis rate but were less sensitive to DGKi. These data suggest that DGKk‐dependent conversion of DAG into PA modulates global protein synthesis level, and DGK inhibitors, by blocking DGK enzymatic activity, counteract the decrease of translation rate. In addition, expression of ∆N‐DGKk induced the phosphorylation of mTOR (Ser2448) in basal conditions and after serum stimulation (Fig EV2B), in agreement with increased DGK‐activity‐dependent PA level (Avila‐Flores *et al*, 2005). Full‐length DGKk expression gave similar yet less‐pronounced effect, probably related to its lower expression level. Conversely, ∆N‐mDGKk expression reduced eIF4E phosphorylation (Ser209), mostly visible after serum activation, in agreement with a reduction of intracellular DAG level (Wang *et al*, 1998). Altogether, these data indicate that ∆N‐DGKk has conserved its ability to modulate DAG/PA and protein synthesis levels.

To assess the biodistribution and efficacy of ∆N‐DGKk in an FXS mouse model, we built the expression cassette to be driven by the neuron‐specific promoter synapsin and packaged into two different adeno‐associated viral vectors (AAV), Rh10 and PHP.eB (Chan *et al*, 2017). Both AAV vectors harboring ∆N‐DGKk demonstrated efficient neuronal transduction in *Fmr1*‐KO cortical neurons, confirming that ∆N‐DGKk can be expressed in the absence of FMRP (Fig 2C). Phosphorylation of initiation factor eIF4E, which correlates with protein synthesis (Sonenberg, 1994) and DAG‐signaling activation (Wang *et al*, 1998), is increased in *Fmr1*‐KO mouse and FXS patients cerebral extracts (Gantois *et al*, 2017). We show that P‐eIF4E is also increased in *Fmr1*‐KO mouse cortical neurons compared to WT littermates and ∆N‐DGKk expression is able to normalize eIF4E phosphorylation (Fig 2D).

**Figure EV3:**
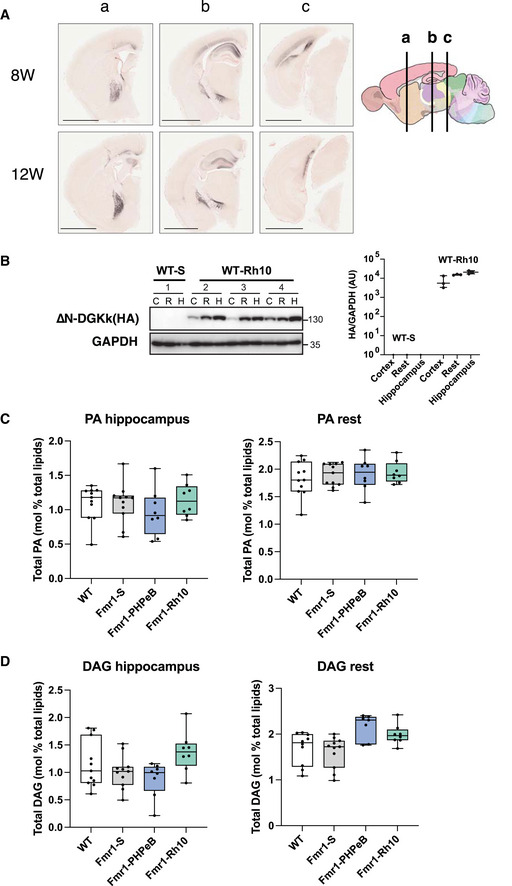
∆N‐DGKk expression in brain with AAV vectors is stable over time and normalizes abnormal phosphatidic acid level in *Fmr1*‐KO cortex Representative coronal brain sections processed for detection of ∆N‐DGKk (HA) at 8 and 12 weeks post‐injections using immunohistochemistry on *Fmr1*‐KO mice treated with indicated treatment, counterstained with eosin hematoxylin. Three regions, a, b, c, are shown with their corresponding position on brain sagittal map. Scale bar 2 mm. Image of brain 8W b is a reuse of Fig 3D Rh10.Immunoblots and quantification of ∆N‐DGKk protein in Fmr1‐WT brain lysates from cortex (c), hippocampus (h), and rest (r) areas as in Fig 3C. GAPDH was used as a loading control. AU, arbitrary units.Measure of total phosphatidic acid (PA) level by mass spectrometry in hippocampus and rest of brain of WT mice treated with saline solution (WT) and *Fmr1*‐KO mice treated with saline (Fmr1‐S), AAVPHP.eB‐∆N‐DGKk (Fmr1‐PHP.eB), AAVRh10‐∆N‐DGKk (Fmr1‐Rh10) 8 weeks after injections. Data are expressed as mol % of total lipids and analyzed using one‐way ANOVA and Tukey’s multiple comparisons test, *n* = 8 individual animals, except for WT and Fmr1‐S *n* = 11 and represented as median with interquartile range with minimum and maximum values.Total diacylglycerol (DAG) level in cortex measured as in C). Source data are available online for this figure.

**Figure EV4:**
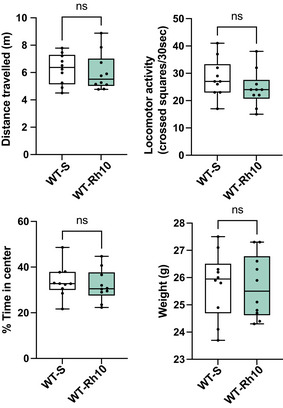
AAVRh10‐∆N‐DGKk does not lead to visible behavior effect in *Fmr1*‐WT mice 4 weeks after its administration Locomotor activity (distance in m and crossed squares per 30 s) in the whole arena and percentage of time spent in the center during 15 min habituation, weight of vehicle‐treated WT mice (WT‐S) compared to AAVRh10‐∆N‐DGKk‐treated WT mice (WT‐Rh10). Data information: Data are expressed as median with interquartile range with minimum and maximum values. Statistical analysis: unpaired *t*‐test (*n* = 10 mice per group), ns not significant. Source data are available online for this figure.

**Figure EV5:**
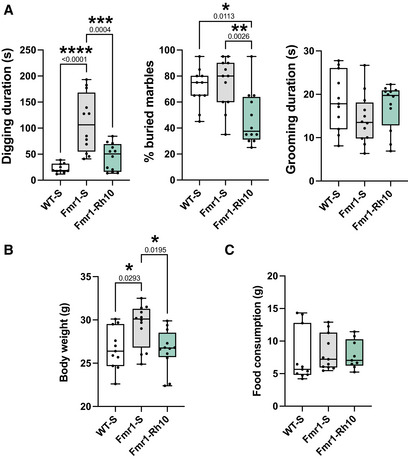
Behavioral analyses of AAVRh10‐∆N‐DGKk‐ treated *Fmr1*‐KO mice (8 weeks after injection) Digging, marble burying, and grooming duration tests.Body weight of mice (g).Food consumption (g). Data information: Data are expressed as median with interquartile range with minimum and maximum values for other panels. Statistical analysis: one‐way ANOVA with Tukey’s multiple comparisons test (*n* = 12) *P < 0.05, **P < 0.01, ***P < 0.001, ****P < 0.0001. Source data are available online for this figure.

No sign of ∆N‐DGKk‐specific neuronal toxicity was observed in neuronal cultures transduced with AAV expressing ∆N‐DGKk at high multiplicity of infection (MOI) (Fig EV2, EV3, EV4, EV5). An overall reduction in NeuN positive cells was observed upon treatment of cells with AAV, independently from ∆N‐DGKk expression, as the same effect was observed with AAV‐FMRP. Such effect was visible with a 10‐fold lower amount of AAV‐FMRP (10e9 VG/well) (Fig EV2E). Additional tests (caspase 3/7, LDH) did not show signs of toxicity *in vitro* (Fig EV2C and D).

### ∆N‐DGKk corrects cortical phosphatidic acid level in *Fmr1*‐KO adult mice using multiple routes of administration

**Figure 3:**
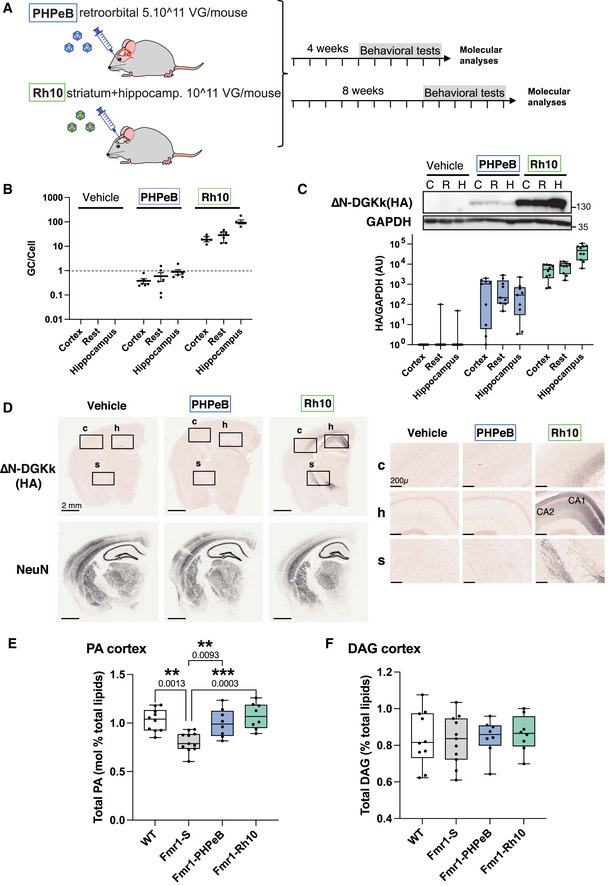
∆N‐DGKk expression *in vivo* with AAV vectors corrects abnormal cortical phosphatidic acid level of Fmr1‐KO mice Scheme of timeline analyses performed.Viral titers (viral genome copy VG per cell) determined by qPCR in cortical, hippocampal, and rest of brain areas of *Fmr1*‐KO mice treated with saline solution (Vehicle), AAVPHP.eB‐∆N‐DGKk (PHP.eB), AAVRh10‐∆N‐DGKk (Rh10) 8 weeks after injections. Data are mean ± SEM. Each dot represents an individual mouse.Immunoblots and quantification of ∆N‐DGKk protein in lysates from brain areas of mice treated as in B. GAPDH was used as a loading control. Densitogram of ∆N‐DGKk expression is presented as change relative to vehicle conditions. Data are expressed as median with interquartile range with minimum and maximum values and analyzed (*n* = 5).Representative coronal brain sections processed for detection of ∆N‐DGKk using immunohistochemistry on *Fmr1*‐KO mice treated with indicated treatment, 8 weeks post‐injections, counterstained with eosin hematoxylin. Adjacent sections were immunolabeled with NeuN. Three mice per genotype were processed. The sections shown are between Bregma levels –1.50 mm and –1.80 mm. Scale bar is 2 mm. Magnifications of regions of cortex (c), hippocampus (h), and striatum (s) are shown in side panels, scale bar 200 µm.Total phosphatidic acid (PA) level measure by mass spectrometry in cortex of WT mice treated with saline solution (WT‐S) and *Fmr1*‐KO mice treated with saline (Fmr1‐S), AAVPHP.eB‐∆N‐DGKk (Fmr1‐PHP.eB), AAVRh10‐∆N‐DGKk (Fmr1‐Rh10) 8 weeks after injections. Data are expressed as mol % of total lipids and represented as median with interquartile range with minimum and maximum values. Statistics: one‐way ANOVA and Tukey’s multiple comparisons test, *n* = 8 individual animals, except for WT‐S and Fmr1‐S *n* = 11. **P < 0.01, ***P < 0.001.Total diacylglycerol (DAG) level in cortex measured as in E. Source data are available online for this figure.

∆N‐DGKk was administered to 5‐week‐old *Fmr1*‐KO mice by intravenous injection of AAVPHP.eB‐∆N‐DGKk or intracerebral injection into the striatum and hippocampus of AAVRh10‐∆N‐DGKk, respectively. Single retro‐orbital injection of AAVPHP.eB‐∆N‐DGKk at 10^11^ VG/mouse enabled 0.5‐1 VG/cell throughout the brain (Fig 3A and B) 4 or 8 weeks after injection, with low protein expression as visualized by Western blot (Fig 3C) and immunohistochemistry (Fig 3D). Intracerebral injection of AAVRh10‐∆N‐DGKk at 5 × 10^11^ VG/mouse enabled higher brain transduction, with about 100 VG/cell in the hippocampus and about 25 VG/cell in rest of brain leading to high protein expression levels (Fig 3B–D). AAVRh10 vector led to robust ∆N‐DGKk expression throughout the hippocampus (CA1 and CA2 regions mainly), cortex, and striatum (Figs 3D and EV3A). Similar expression of AAVRh10‐∆N‐DGKk was also obtained in *Fmr1*‐WT mice (Fig EV3B).

DGKk deregulation was shown to alter DAG/PA balance in neuronal cultures and human postmortem brains (Tabet *et al*, 2016a). We confirmed a marked decrease of total PA level (22% ±8) in the cortex of *Fmr1*‐KO mice at 13 weeks of age by tandem mass spectrometry analysis (Fig 3E). The other mouse brain regions did not show significant differences (Fig EV3C and D). The decrease in the total pool of PA is reflected by a reduction of most PA species (including abundant species 34:1, 36:2, 38:4 or low abundance species 38:3, 40:1; Appendix Fig S1A–C). Thus, DGKk loss in *Fmr1*‐KO cortex led to an alteration of PA synthesis independent from fatty acid patterns. ∆N‐DGKk delivery to the brain alleviated PA reduction in *Fmr1*‐KO and led to a PA level comparable to vehicle injected WT mice (Fig 3E). This is true for all fatty acid PAs, with levels undistinguishable from control, demonstrating a rescue of PA level by ∆N‐DGKk expression (Appendix Fig S1A–C). Intravenous delivery of PHP.eB‐∆N‐DGKk led to partial correction of total PA level (Fig 3E) that was further confirmed at the individual PA species level, indicating that ∆N‐DGKk is able to balance the PA with only few neurons transduced (Appendix Fig S1A–C). Total DAG level was not significantly altered in the cortex (Fig 3F) and the other brain areas tested (Fig EV3D), and at the level of individual DAG species (Appendix Fig S2A–C). Nine other lipid classes tested did not show significant differences between the groups (Appendix Fig S3) suggesting that the effect of ∆N‐DGKk is restricted to the correction of DAG/PA balance.

### AAVRh10‐∆N‐DGKk achieves long‐term rescue of *Fmr1*‐KO mice behavioral defects

Four weeks after injections, a battery of behavioral tests was performed on the AAV injected *Fmr1*‐KO and *Fmr1*‐WT mice and their vehicle injected WT (WT‐S) or *Fmr1*‐KO (Fmr1‐S) littermate controls (Table 1). To control that ∆N‐DGKk did not induce nonspecific behavioral alterations, a cohort of *Fmr1*‐WT mice was intracerebrally injected with the AAVRh10‐∆N‐DGKk vector that provides the highest ∆N‐DGKk expression level among the tested conditions (Fig 3B and C) or vehicle treated. Extensive SHIRPA modified tests encompassing 27 separate observational parameters (Hatcher *et al*, 2001) did not reveal any sensorimotor deficit in the AAVRh10‐∆N‐DGKk treated mice compared to the vehicle injected group (Appendix Table S1).

**Table 1:**
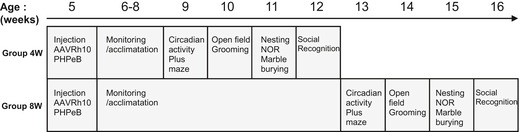
Phenotyping pipeline.

**Figure 4:**
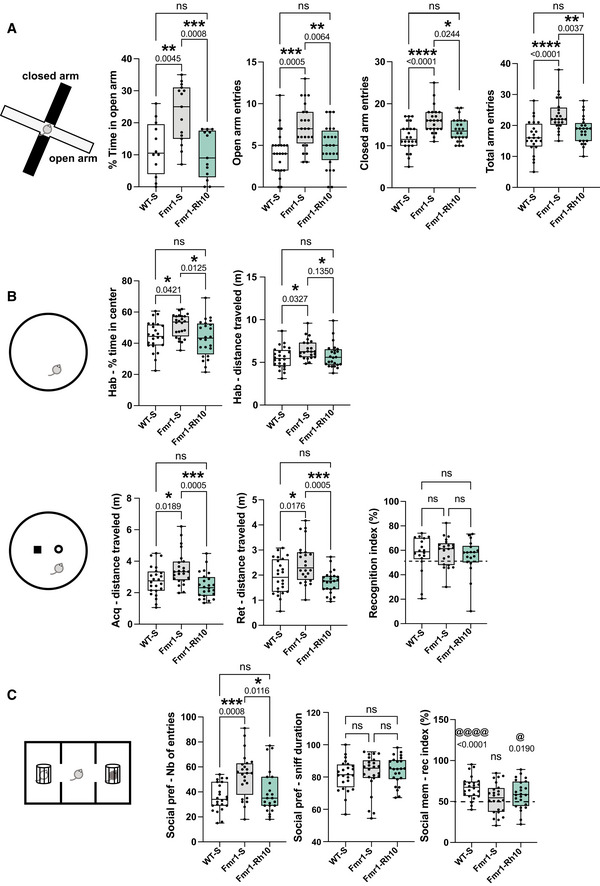
AAVRh10‐∆N‐DGKk rescues behavior alterations of *Fmr1*‐KO mice 4 weeks after injections Elevated Plus Maze. Percentage of time spent in open arms and number of entries in open, closed, and total (open+closed) arms (*n* = 12 for time in open arm, *n* = 24 for arm entries).Novel object recognition. Locomotor activity (distance in m) in the whole arena and percentage of time spent in the center during 15 min habituation. Locomotor activity during the acquisition and retention trials. Recognition index (*n* = 24).Social recognition. Number of entries in the two side compartments during preference session (left), social preference (percentage of exploration of a congener vs. an object) (center), and social memory (percentage of exploration of a novel vs. familiar congener) (right) (*n* = 24). Data information: Data are expressed as median with interquartile range with minimum and maximum values and analyzed using one‐way ANOVA and Tukey’s multiple comparisons test : *P < 0.05, **P < 0.01, ***P < 0.001, ****P < 0.0001; and one group *t*‐test (recognition index): ^@^ *P* < 0.05, ^@@@@^ *P* < 0.0001 vs chance (50%). Source data are available online for this figure.

The vehicle injected *Fmr1*‐KO mice showed an increase of time spent and in number of entries in the open arms of the elevated plus maze (EPM) compared to the WT‐vehicle mice (1.9‐ and 1.8‐fold of mean increase, respectively), suggesting a decreased anxiety induced by the genotype. In contrast, the AAVRh10‐∆N‐DGKk‐treated mice (Fmr1‐Rh10) showed no difference compared to the WT‐S mice (Fig 4A). A similar phenotype was observed in the open field arena of novel object recognition test (NOR), where vehicle injected *Fmr1*‐KO mice showed an increase of time spent in the center of the arena, while AAVRh10‐∆N‐DGKk treated *Fmr1*‐KO mice were not different from WT‐vehicle (Fig 4B). Effect of the AAVRh10‐∆N‐DGKk treatment seemed to be specific of the *Fmr1*‐KO genotype since it had no significant effect in WT context in these tests (Fig EV4).

Vehicle injected *Fmr1*‐KO mice showed increased locomotor activity compared to WT‐vehicle mice. This phenotype was seen in the EPM test (Fig 4A, number of entries in open and closed arms), in the open field arena of NOR (Fig 4B, distance traveled in habituation, acquisition and retention phases) and in the habituation phase of three‐chambers social recognition test (Fig 4C, number of entries), and was corrected in all tests with AAVRh10‐∆N‐DGKk treatment. Signs of hyperactivity of *Fmr1*‐KO mice were also seen in the first hour of light and dark phases of actimetry test (Appendix Fig S4A and B), suggesting this phenotype is related to novelty of environment. AAVRh10‐∆N‐DGKk‐treated mice did not show this phenotype. WT‐Rh10 mice did not show reduced locomotor activity compared to vehicle‐treated WT‐S (Fig 4B, distance travelled, Appendix Fig S4A) suggesting again that treatment effect was specific of the genotype. No difference in recognition index was observed between genotypes in the NOR test (Fig 4B).

Performing the NOR test in a smaller size arena ameliorated the recognition index of objects (Appendix Fig S5B), in correlation with longer object exploration times of objects (Appendix Fig S5A vs B, explorations). However, no difference was observed between the genotypes, suggesting an apparent lack of significant memory impairment of the *Fmr1*‐KO mice. In small size arena, the hyperlocomotor activity phenotype of *Fmr1*‐KO mice was not visible (Appendix Fig S5B), potentially because this environment was less anxiogenic than a larger arena. *Fmr1*‐KO vehicle mice showed a trend to dig more, to bury more marbles, and to spend less time in grooming compared to the WT‐vehicle mice, but these differences were not statistically significant (Appendix Fig S5C). ∆N‐DGKk treatment seemed to counteract these effects, but the high variability of these phenotypes between mice precluded drawing conclusions.

Vehicle injected *Fmr1*‐KO mice showed an increased number of entries in the three‐chambers social recognition test and this phenotype was corrected with AAVRh10‐∆N‐DGKk (Fig 4C, Appendix Fig S6). For social preference over object, no difference was observed between genotype groups (Fig 4C, Appendix Fig S6) indicating there was no significant effect of mutation or treatment on social preference. In the social memory test, WT‐vehicle mice showed preference for novel mouse compared to previously encountered mouse (65 ± 2% significantly different than chance, *P* < 0.000, Fig 4C), while *Fmr1*‐KO mice did not show increased interaction with the novel mouse (i.e., recognition index is not statistically different than chance). *Fmr1*‐KO mice treated with AAVRh10‐∆N‐DGKk had a higher recognition index relative to untreated mice and relative to chance (*P* = 0.019) indicating a rescuing effect of treatment. Vehicle injected *Fmr1*‐KO mice also showed a reduced ability to build a nest after 5 and 24 h. AAVRh10‐∆N‐DGKk treatment possibly rescued this phenotype (Fmr1‐Rh10 being not significantly different from WT‐S, Appendix Fig S7).

The phenotypes observed at 4 weeks after injections were generally recapitulated in older animals at 8 weeks after injection (i.e., 13‐week‐old) (Appendix Figs S8 and S9, Fig EV5). Consistent with the stability of the treatment effect, no significant variation of ∆N‐DGKk protein level was observed between 8 and 12 weeks after AAV injection (corresponding to animal groups phenotyped at 4 and 8 weeks, respectively) (Fig EV3A). *Fmr1*‐KO‐vehicle mice tested at 8 weeks showed reduced recognition index in NOR test compared to WT‐vehicle and *Fmr1*‐KO‐Rh10‐∆N‐DGKk (Appendix Fig S9B). However, within a smaller test arena, where *Fmr1*‐KO‐vehicle mice exhibit less hyperactivity (Appendix Fig S9C), all groups performed equally, suggesting that the memory performance alteration of *Fmr1*‐KO mice might be caused by hyperactivity. *Fmr1*‐KO‐vehicle mice tested at 8 weeks after injection showed a strong digging behavior compared to WT‐vehicle and *Fmr1*‐KO‐Rh10‐∆N‐DGKk (Fig EV5A), a phenotype that was not visible at 4 weeks (Appendix Fig S5C).

An increase in body weight was observed in *Fmr1*‐KO‐vehicle group compared to WT‐vehicle. WT‐vehicle and *Fmr1*‐KO‐Rh10‐∆N‐DGKk showed no weight difference, suggesting the treatment rescued this phenotype (Fig EV5B) independently of the food consumption that was not significantly different between the groups (Fig EV5C). WT‐Rh10 mice (Fig EV5B) did not show reduced weight 4 weeks after treatment compared to WT‐S, indicating an absence of a specific effect of treatment on this parameter.

*Fmr1*‐KO‐vehicle phenotypes were reproduced in another cohort aimed at testing retro‐orbital administration of PHP.eB‐∆N‐DGKk (Appendix Fig S10A–F). But unlike the mice which received AAVRh10‐∆N‐DGKk (and except for distance travelled during retention phase of NOR, Appendix Fig S10D), *Fmr1*‐KO‐PHP.eB‐∆N‐DGKk mice showed no significant improvement compared to *Fmr1*‐KO‐vehicle, suggesting that the level of ∆N‐DGKk, although well distributed throughout the brain, was too low to achieve a sufficient effect. Macroorchidism, a well‐established phenotype of *Fmr1*‐KO model, was not found corrected by the treatments (Appendix Fig S11), possibly reflecting a non‐neuronal origin.

## Discussion

There are currently no disease‐modifying treatments for FXS (Yamasue *et al*, 2019). In this study, we provide evidence that neuron‐targeted expression of a modified DGKk enzyme with an AAVRh10‐based gene therapy approach is able to provide long‐term correction of disease‐relevant behavioral abnormalities of the young adult *Fmr1*‐KO mouse model of FXS (Appendix Table S2).

DGKk is an enzyme whose mRNA was previously found to be a most prominent target of FMRP in cortical neurons (Tabet *et al*, 2016a). We show that DGKk expression is strongly dependent on FMRP and severely altered in FXS brain. In fact, to our knowledge, no other protein has been demonstrated yet to be so critically dependent upon FMRP. Being a member of master regulator of second messenger lipids DAG/PA balance, DGKk could play a critical role in manifestation of FXS phenotypes because its loss of activity has the potential to be a triggering cause of the many altered neuronal signaling pathways observed in FXS (Tabet *et al*, 2016b). Loss of DGKk activity is expected to cause cellular DAG excess and lack of PA. While an excess of DAG has been seen in dissociated *Fmr1*‐KO cortical neuron cultures (Tabet *et al*, 2016), such excess was not observed in the three brain region homogenates analyzed at 15 weeks of age. PA, instead, was seen diminished but only in cortex homogenates. While we have no definitive explanation for these observations, metabolic buffering compensations (including from the other DGK isozymes) may exist to counteract an increase of DAG in *Fmr1*‐KO, alternatively, the fact that *DGKk* is one of the least expressed genes of the DGK family (https://gtexportal.org/home/gene/DGKK) and its exquisite expression in restricted neuron populations indicated by spatially resolved transcriptomics (Hu *et al*, 2021) might have hindered the measures in the brain homogenates.

Removal of the 5′UTR region and N‐terminal part of DGKk does not impact DGKk activity *in vitro* (Imai *et al*, 2005), while it abolishes regulation by FMRP (Figs 1C and EV1A) and enables its synthesis in conditions of absence of FMRP. The FMRP‐independent ∆N‐DGKk protein conserved its ability to modulate cell signaling (Fig EV2B) and showed capacity to rescue disease‐relevant behavioral abnormalities of the *Fmr1*‐KO mouse while administered in developed brain (Fig 4).

Among all the behavioral tests we performed, the most pronounced phenotypes were seen during habituation phases (Appendix Table S2), which suggests that *Fmr1*‐KO mice are hyperactive when faced with novelty and which correlates to the well‐established deficit in habituation, hypersensitivity, and hyperactivity observed in patients with FXS (Hagerman *et al*, 2017). Several tests showed apparent contradiction with some previous studies (e.g., no or little impact was seen on object and social memory, lower anxiety instead of higher anxiety was seen in open field and plus maze). These tests have shown variability across studies (Santos *et al*, 2014) and may suffer from prevailing hyperactivity phenotype that can bias anxiety and memory tests.

At the AAV dose used, intravenous administration of AAVPHP.eB‐∆N‐DGKk was unable to rescue the *Fmr1*‐KO phenotype, presumably because of insufficient, albeit homogenous expression in the brain. In contrast, intracerebral injection of AAVRh10‐∆N‐DGKk led to higher expression of ∆N‐DGKk in the mouse brain and correction of disease phenotypes, without affecting survival and with no signs of toxicity several weeks after dosing. Remarkably, these effects seem to be specific of the *Fmr1*‐KO genotype since no adverse effect was detected in WT context. Consistently, high ∆N‐DGKk expression in neuronal cultures was not associated with cellular toxicity. Rescuing *Fmr1‐KO* mouse with AAV‐based FMRP administration at an early stage (P0‐P5) has provided the first proof of concept of a gene therapy approach for FXS (Gholizadeh *et al*, 2014; Hampson *et al*, 2019), but also revealed that inappropriate levels of FMRP expression can lead to worsening of FXS phenotypes, possibly due to the fact that FMRP, like other RNA‐binding proteins, induces cellular stress when overexpressed (Mazroui *et al*, 2002). *Fmr1*‐KO mice have been shown to exhibit increased nonfat body weight (Leboucher *et al*, 2019), a phenotypic trait found in a proportion of FXS patients (Hagerman *et al*, 2017; de Vries *et al*, 1995). The origin of this phenotype in patients is unknown. The apparent ability of neuronal ∆N‐DGKk to interfere with this phenotype independently of food intake (Fig EV5B and C) suggests a cerebral origin linked to DAG/PA signaling. Macroorchidism in contrast could be of non‐neuronal origin or with a closed developmental window.

Overall, the rescue of the *Fmr1*‐KO phenotypes with ∆N‐DGKk expression in neurons strengthened the notion that DAG/PA imbalance in neurons is a critical factor of the disease and acting on this imbalance is beneficial for FXS‐like condition, including at a late stage of development. Use of ∆N‐DGKk could offer potential for FXS gene therapy, representing a very specific target in the complex pathomechanism of the disease.

## Materials and Methods

### Animal model

Mouse strain B6.129P2‐Fmr1tm1.2Cidz/J (also called Fmr1‐KO2 model, missing exon 1 (Mientjes *et al*, 2006) males from same genotype (thereafter called *Fmr1*‐KO or *Fmr1*‐WT) were grouped by 3 or 4 individuals at weaning age (4 weeks), in individually ventilated cages (GM500, Tecniplast, UK), with poplar shaving bedding (Lignocell Select, JRS, Germany), and maintained under standard conditions, on a 12‐h light/dark cycle (7 h/19 h), with standard diet food (standard diet D04, Scientific Animal Food and Engineering, France) and water available *ad libitum*. Mice from a same cage received the same treatment and were transferred in the animal facility of the phenotyping area the next week.

Animal work involved in this study was conducted according to ARRIVE guidelines and received authorization from relevant national committee (Comité National de Réflexion Ethique en Expérimentation Animale) with number APAFIS#5874‐20l6062915583967 v2 and APAFIS #17544‐2018111516571205 v7 at the ICS mouse facility (Illkirch, France). Every procedure on mice was performed with the aim of ensuring that discomfort, distress, pain, and injury would be minimal.

### Cloning of human and mouse DGKk mRNA

Mouse DGKk was subcloned from clone IMAGE IRAVp968H03163D. The missing 3′ UTR and 5′ UTR‐N‐ter region were cloned by PCR from mouse genomic DNA with primer sets (GCAGCTAGCTCCTTGAAAGCTGGAAGGAGA and AATAGAATGCGGCC‐GCCAGCTTCAACAGCACTTGTAG) and (CCAgtcgacTTAGACCTCAGAGCTGCGCTAGC and CCAgctagcCCAGGACTCTGGGGCCCTCTCCAT), respectively. The 3′UTR region was introduced at XbaI and NotI sites of the pYX‐∆N DGKk vector to give pYX‐∆N‐DGKκ‐3′UTR, and the 5′‐UTR‐Nter region at SalI‐NheI sites of the pYX‐DGKκ‐3′UTR, NheI site, was subsequently deleted by PCR mutagenesis. pCI‐mDGKk‐HA and pCI‐HA‐∆N‐DGKk were obtained by PCR subcloning into pCI vector (GenBank U47119) with addition of the HA sequence before the STOP codon or after ATG, respectively. Human hDGKk was subcloned from plasmid pAcGFPC1humDGKk (Imai *et al*, 2005) into Nhe1 of pCI vector with addition of 5′ and 3′UTRs by PCR cloning to produce pCI‐hDGKk.

### AAV ∆N‐DGKk vectors construction and preparation

∆N‐DGKk HA‐tagged DGKk was cloned under the control of the hSynapsin promoter replacing EGFP in the control plasmid pENN.AAV.hSynapsin.EGFP.RBG (provided by the Penn Vector Core at University of Pennsylvania, Philadelphia) to give pAAV‐∆N‐DGKk. Recombinant adeno‐associated virus serotype 9 (AAV9), Rh10 (AAVRh10), PHP.eB (AAVPHP.eB) production was carried out by using the AAV Helper‐Free system (Agilent Technologies) with some modifications. AAV vectors were generated by triple transfection of 293T/17 cell line using Polyethylenimine (PEI) and plasmids pAAV‐hsynapsin‐HA‐∆N‐DGKk or pENN.AAV.hSynapsin.EGFP.RBG together with pHelper (Agilent) and pAAV2/9 or pAAV2/Rh10 (provided by J. Wilson and J. Johnston at Penn Vector Core), or pUCmini‐iCAP‐PHP.eB (provided by V. Gradinaru and J. Johnston) for serotypes 9, Rh10, and PHP.eB, respectively. Two days after transfection, cells were collected, lysed by three freeze/thaw cycles in dry ice‐ethanol and 37°C baths, further treated with 100 U/ml Benzonase (Novagen) for 30 min at 37°C, and clarified by centrifugation at 3,000 *g* for 15 min. Viral vectors were purified by iodixanol (Optiprep, Axis Shield) gradient ultracentrifugation followed by dialysis and concentration against PBS containing 0.5 mM MgCl_2_ using centrifugal filters (Amicon Ultra‐15.100 K) and filtered through 0.22 u (Zolotukhin *et al*, 2002). Viral particles were quantified by real‐time PCR Q‐PCR using LightCycler480 SYBR Green I Master (Roche) and primers targeting the flanking sequence of ITR2 (GTAGATAAGTAGCATGGC and CTCCATCACTAGGGGTTCCTTG) or the flanking sequence of rabbit β‐globin polyadenylation signal (CCCTTGAGCATCTGACTTCTGG and AGGGTAATGGGTATTATGGGTGGT). To achieve comparable working concentrations, viruses were diluted to a final concentration of 1 × 10^13^ viral genome per ml (VG/ml) and stored at −80°C until use.

### Cell culture and transfections

COS‐1 and HeLa cells were from ATCC and have been tested for negative mycoplasma contamination. Cells were grown in DMEM supplemented with 10% (v/v) FCS and 1 g/l glucose in the presence of 40 μg/ml gentamicin at 37°C in 5% CO2. The day before transfection, 4 × 10^4^ cells were plated into 24‐well format plates in 500 μl of antibiotic‐free medium. Transfections of plasmids were performed in triplicate with Lipofectamine 2000 (Invitrogen) as directed by the manufacturer with 10 pmol of siRNA (Control or On Target plus Smart Pool mouse *FMR1*; Thermo Fisher Scientific) in a final volume of 600 μl. Twenty‐four hours later, 300 ng of the reporter pCI‐DGKκ‐HA or pCI‐HA‐∆N‐DGKk and 10 nM shRNA were cotransfected as above. Twenty‐four hours later, cells washed twice in PBS were lysed directly in 4× Laemmli buffer (Tris–HCl 120 mM pH 7, SDS 8%, DTT 0.1 M, glycerol 32%, bromophenol blue 0.004%, β‐mercaptoethanol 1%) for 3 min at 95°C. For serum starvation experiments, the medium was replaced by 0% FCS medium 12 h post‐transfection. After 24 h, the medium was replaced by 10% FCS medium and incubated for the indicated time period (5‐15‐30 min). For the 0 min time point, the medium was not replaced. Cells were then washed rapidly with cold PBS and lysed directly in 4× Laemmli buffer.

### Primary cortical neuron cultures and treatments

Neuron cultures were performed as described previously (Tabet *et al,* 2016). Briefly, cortices from C57BL/6J *Fmr1*‐WT or *Fmr1*‐KO mouse embryos (embryonic day E17.5) were dissected in 1xPBS, 2.56 mg/ml d‐glucose, 3 mg/ml BSA, and 1.16 mM MgSO4, incubated for 20 min with 0.25 mg/ml trypsin and 0.08 mg/ml DNase I, and mechanically dissociated after supplementation of medium with 0.5 mg/ml trypsin soybean inhibitor, 0.08 mg of DNase I, and 1.5 mM MgSO_4_. The cells were plated on poly‐L‐lysine hydrobromide‐coated six‐well culture plates for 8 days in Neurobasal Medium (GIBCO) supplemented with B27, penicillin/streptomycin, and 0.5 μM l‐glutamine. Where indicated, cultures were treated with addition of puromycin solution at the indicated concentrations and times. DGK inhibitors R59022 (DGK Inhibitor I; Calbiochem) and R59949 (DGK Inhibitor II; Calbiochem) were applied at concentrations of 3 and 0.2 μM each for 15 min at 37°C. After treatment, cells were immediately washed with ice‐cold PBS and lysed in 4× Laemmli buffer.

### Western blot analyses

Immunoblotting was performed as described previously (Tabet *et al*, 2016a). Proteins (equivalent to 15 µg) were denatured 5 min at 95°C and resolved by 10% SDS–PAGE. Separated proteins were transferred onto PVDF Immobilon P membrane (Millipore) using a Trans‐Blot Turbo Transfer System and Trans‐Blot Turbo RTA Transfer Kit (Biorad). Membranes were blocked for 1 h with TBS‐T 1X (Tris‐Buffer Saline, pH 7.4 and 0.1% Tween‐20 v/v) containing 5% (w/v) BSA or 5% nonfat dry milk. Membranes were incubated overnight at 4°C with primary antibodies (Appendix Table S3) diluted in TBS‐T buffer containing 5% w/v BSA or milk. Membranes were washed in TBS‐T buffer and then incubated for an hour at room temperature with the corresponding horseradish peroxidase‐conjugated pre‐adsorbed secondary antibody (1:5,000, blocking solution corresponding, Molecular Probes). Membranes were washed in TBS‐T buffer and immunoreactive bands were visualized with the SuperSignal West Pico Chemiluminescent Substrate (Pierce). Immunoblot pictures were acquired using LAS600 GE Amersham and density of the resulting bands was quantified using ImageJ and data represented with GraphPad Prism 7 software.

### Analysis of cortical neuron cultures by immunofluorescence microscopy

Primary cortical neurons grown on poly‐L‐lysine hydrobromide‐coated glass coverslips were fixed in 4% (w/v) paraformaldehyde (PFA) in 1xPBS at room temperature (RT) for 20 min, permeabilized with 1X PBS and 0.2% Triton X‐100 for 10 min at RT, and blocked for 1 h in 1x PBS and 0.1% Triton X‐100 with 5% (w/v) BSA. Neurons were incubated with primary antibodies (Appendix Table S3) overnight at 4°C. After three washes in 1× PBS and 0.1% Triton X‐100 for 10 min, neurons were incubated with secondary antibody anti‐rabbit and anti‐mouse (Appendix Table S3) for 1 h at RT, and subsequently washed three times in 1xPBS and 0.1% Triton X‐100 for 10 min. Coverslips were mounted with antifading medium (Vectashield, Vector) with DAPI and analyzed by fluorescence microscopy. Images were acquired with CellInsight CX7 (Thermo Scientific) using a 10× objective and analyzed with HCS Studio Cell Analysis Software (nuclear segmentation, NeuN and GFAP intensities). Quantification of positive cells for each of these staining was done by applying a threshold manually, based on nuclear segmentation and across 81 fields. Percentage of cells positive for NeuN and GFAP staining was quantified for each well.

### Caspase 3/7 activity detection

Caspase 3/7 positive cells were determined with CellEvent Caspase‐3/7 Green Detection Reagent following manufacturer’s instructions. Briefly, primary neurons were prepared as for LDH assays and treated with reagent diluted at 8 µM in 5% FBS NBM. Positive control wells were treated with apoptotic inducer staurosporine at 0.1 and 1 µM for 6 h. Cells were fixed in 4% (w/v) PFA and nuclei were counterstained with DAPI. Images were acquired with CellInsight CX7 (Thermo Scientific) using a 10x objective and analyzed with HCS Studio Cell Analysis Software (nuclear segmentation and casp3/7 intensities). Quantification of percentage of caspase‐3/7 positive cells was done for each well by applying a threshold manually, based on nuclear segmentation and across 81 fields.

### Lactate dehydrogenase releasing assay

Lactate dehydrogenase (LDH) release was determined with the Cytotoxicity Detection KitPLUS kit (Roche) following manufacturer’s instructions. Briefly, primary neurons from *Fmr1‐*WT or *Fmr1*‐KO E17.5 embryos plated at 300,000 cells/well in 24‐well plate and transduced at 7 DIV with indicated AAV were tested after 7 days. LDH release was measured in microplate reader at 490 nm after 2 h at 37°C with reaction medium. Maximum LDH release was measured in same conditions after 30 min at RT with stop solution. The % of cell death was determined using the formula: % cell death = experimental LDH release /maximum LDH release.

### Stereotaxic surgery and AAV injections

Five‐week‐old mice C57BL/6J *Fmr1*‐KO or C57BL/6J *Fmr1*‐WT littermates were deeply anesthetized with ketamine/xylazine (Virbac/Bayer, 100/10 mg/kg, 13 ml/kg, intraperitoneal) dissolved in sterile isotonic saline (NaCl 0.9%) and mounted onto a stereotaxic frame (World Precision Instruments). AAVRh10‐DGKk were injected bilaterally into the striatum (coordinates relative to bregma: anterior‐posterior + 0.5 mm; lateral = ±2.2 mm; vertical −3.5 mm) and hippocampus (coordinates relative to bregma: anterior‐posterior ‐ 1.7 mm; lateral = ±1.5 mm; vertical −2.0 mm) according to the mouse brain atlas (Paxinos & Franklin 2001). A volume of 2.5 μl of AAV vector (corresponding to 10^e^11 Genome copies) or saline solution was delivered bilaterally per site of injection with a slow injection rate (0.2 μl/min) through a 32‐gauge small hub removable needle mounted on a 10 µl Hamilton syringe connected to a micropump (World Precision Instruments). After each injection was completed, the injector was left in place for an additional 2 min to ensure optimal diffusion and minimize backflow while withdrawing the injector.

### Retro‐orbital AAV injections

Five‐week‐old mice C57BL/6J *Fmr1*‐KO or C57BL/6J *Fmr1*‐WT littermates were deeply anesthetized with ketamine/xylazine (Virbac/Bayer, 100/10 mg/kg, 10 ml/kg, intraperitoneal) dissolved in sterile isotonic saline (NaCl 0.9%). AAVPHP.eB‐DGKk were injected retro‐orbitally with a volume of 80 µl.

### Analysis of ∆N‐DGKk expression in brain sections

Freshly dissected brains were fixed overnight in PFA 4% and stored in PBS1X before being processed following Neuroscience Associates procedure https://www.neuroscienceassociates.com/technologies/multibrain/. Half‐brains were washed in PBS1X solution and embedded in gelatin matrix. MultiBrain^®^ cryosections were prepared with 30 µm thickness for free‐floating immunolabeling with anti‐HA 3F10 (Appendix Table S3) and counter stained with hematoxylin/eosin. Adjacent sections were immunolabeled with anti‐NeuN (Appendix Table S3).

### Behavioral experiments

Behavioral experiments were conducted 4 or 8 weeks after AAV injections to allow sufficient time for viral transduction and DGKk expression. Effective gene expression was assessed by q‐PCR to measure viral titer, by western blot and by immunohistochemistry in three different brain areas (cortex, hippocampus and rest of brain). The behavioral studies were conducted under experimenter‐independent conditions or by experimenters naive to the treatment conditions. Phenotyping pipeline is described in Table 1. Individual cohorts of littermate animals (issued from *Fmr1* ^y/+^ × *Fmr1* ^+/−^ mating) composed of WT‐vehicle, *Fmr1*‐KO‐vehicle, *Fmr1*‐KO‐AAV subgroups were constituted for each treatment (Rh10, PHPeB) and treatment time (4 weeks, 8 weeks). An additional independent cohort was constituted to analyze treatment effect in WT animals. Analyses were performed independently on each cohort.

### Gross neurological examination

General health and basic sensory‐motor functions were evaluated using a modified SHIRPA protocol as described in (Hatcher *et al*, 2001). This analysis provides an overview of physical appearance, body weight, neurological reflexes, and sensory‐motor abilities (Ouagazzal *et al*, 2003).

### Circadian activity

Spontaneous locomotor activity and rears are measured using individual cages (20 × 10 × 8 cm) equipped with infrared captors. The quantity of water and food consumed is measured during the test period using automated pellet feeder and lickometer (Imetronic, Pessac, France). Mice are tested for 32 h in order to measure habituation to the apparatus as well as nocturnal and diurnal activities. Results are expressed per 1 h periods and/or as a total of the different activities.

### Elevated plus maze

The apparatus used is completely automated and made of PVC (Imetronic, Pessac, France). It consists of two open arms (30 × 5 cm) opposite one to the other and crossed by two enclosed arms (30 × 5 × 15 cm). The apparatus is equipped with infrared captors allowing the detection of the mouse in the enclosed arms and different areas of the open arms. Mice were tested for 5 min during which the number of entries into and time spent in the open arms were measured and used as an index of anxiety. Closed arm entries and total arm entries were used as measures of general motor activity.

### Novel object recognition task

Mice were tested in a circular arena (50 cm diameter and 30 cm height basin). The locomotor activity was recorded with the EthoVision XT video tracking system (Noldus, Wageningen, Netherlands). The arena was virtually divided into central and peripheral regions and homogeneously illuminated at 40 Lux. Animals were first habituated to the arena for 15 min. Each mouse was placed in the periphery of the arena and allowed to explore freely the apparatus, with the experimenter out of the animal’s sight. The distance traveled and time spent in the central and peripheral regions were recorded over the test session. The percentage of time spent in center area was used as index of emotionality/anxiety. The next day, mice were tested for object recognition in the same arena. They were submitted to a 10‐min acquisition trial during which they were placed in the arena in presence of a sample objects (A and A′) (2.5 cm diameter marble or 2 cm edge plastic dice). The time the animal took to explore the samples (sniffing) was manually recorded. A 10‐min retention trial was performed 24 h later. During this trial, one of the samples A and another object B (marble or dice depending on acquisition) were placed in the open field, and the times tA and tB the animal took to explore the two objects were recorded. A recognition index (RI) was defined as (tB / [tA + tB]) ×100.

### Nest building

On the day of test, mice were singly transferred in a standard cage for the duration of nest building measurement. A block of nesting material (5 × 5 cm hemp square, Happi Mats, Utopia) was placed in the cage. Pictures were taken and visual scoring occurred at 2, 5, 24 h without disturbing the animals. The room temperature was noted when the nest was scored, since nest building has a thermoregulatory function and therefore may be influenced by ambient temperatures. We used a 0‐5 scale described by (Gaskill *et al*, 2013): 0 = undisturbed nesting material; 1 = disturbed nesting material but no nest site; 2 = a flat nest without walls; 3 = a cup nest with a wall less than ½ the height of a dome that would cover a mouse; 4 = an incomplete dome with a wall ½ the height of a dome; 5 = a complete dome with walls taller than ½ the height of a dome, which may or may not fully enclose the nest.

### Social recognition test

Social recognition test evaluates the preference of a mouse for a congener as compared to an object placed in an opposite compartment. This test is also used for evaluation of social memory by measuring exploration of a novel congener as compared to a familiar one. Social behavior is altered in several diseases such as autism and mental retardation. The apparatus is a transparent cage composed with a central starting compartment and two side compartments where circular grid cup (goal box) is placed at each extremity, and where the congener can be placed during testing. Testing was performed for 2 consecutive days. On the first day, the mouse was placed in central box then allowed to explore freely the apparatus for 10 min in order to attenuate their emotionality. On the second day, a C57Bl/6J congener from the same sex was placed in one goal box and an object was placed in the opposite one. The mouse was then placed in the starting central compartment and allowed to explore freely the apparatus for 10 min. The position of the congener and object boxes was counterbalanced to avoid any potential spatial preference. The duration of exploration of each goal box (when the mouse is sniffing the grid delimiting the goal box) was manually measured and the percentage of time the mouse took to explore the congener was used as index of social preference (recognition preference). A 10‐min retention trial was then performed during which the object was replaced by a novel congener. The duration of exploration of each goal box was manually measured and the percentage of time the mouse takes to explore the congener was used as index of social memory. The social preference index (SR) is defined as (time Congener / [time Object + time Congener]) ×100; and the social memory index as (time novel Congener / [familiar congener + time novel Congener]) ×100.

### Lipidomic analyzes

Nitrogen frozen brain samples (cortex, hippocampus, rest) were let thaw on ice and mechanically homogenized with 1 vol H_2_0 with Precellys 24 system during 2 × 15 s at 4°C and 5,300 rpm. Protein concentration of sample was adjusted at 5 mg/ml concentration and lipids were analyzed on Lipotype GmbH platform. Lipids were extracted using chloroform and methanol (Sampaio *et al*, 2011) with Hamilton Robotics STARlet. Samples were spiked with lipid class‐specific internal standards prior to extraction. After drying and resuspending in MS acquisition mixture, lipid extracts were subjected to mass spectrometric analysis. Mass spectra were acquired on a hybrid quadrupole/Orbitrap mass spectrometer (Thermo Scientific Q‐Exactive) equipped with an automated nano‐flow electrospray ion source in both positive and negative ion mode. Lipid identification using LipotypeXplorer (Herzog *et al*, 2011) was performed on unprocessed (*.raw format) mass spectra. For MS‐only mode, lipid identification was based on the molecular masses of the intact molecules. MSMS mode included the collision‐induced fragmentation of lipid molecules, and lipid identification was based on both the intact masses and the masses of the fragments. Prior to normalization and further statistical analysis, lipid identifications were filtered according to mass accuracy, occupation threshold, noise, and background. Intensity of lipid class‐specific internal standards was used for lipid quantification. The identified lipid molecules were quantified by normalization to a lipid class‐specific internal standard. The amounts in pmol of individual lipid molecules (species of subspecies) of a given lipid class were summed to yield the total amount of the lipid class. The amounts of the lipid classes were normalized to the total lipid amount yielding mol% per total lipids.

### Statistical analyses

Sample size was chosen based on interindividual variability and expected intergenotype effect (known from pilot study or previous literature reports) to reach a desired power of 0.8. For animal studies, sample size was determined based on a pilot study intended to reproduce literature data and enabling detection of 20% range effect with a power of 0.8. No animal or sample was *a priori* excluded from the analyses. Where indicated, outliers were removed from analyses using ROUT method with Q = 1% with GraphPad Prism 9 software. Animals from same genotype were randomly grouped by 3 or 4 by cage and all animals from a given cage randomly received either treatment or placebo. Behavioral studies were conducted under experimenter‐independent conditions (actimetry, three chamber test, elevated plus maze, novel object recognition) or by experimenters naive to expected effect of genotype and treatment (nesting, grooming, digging). Graphs and statistical analyses were performed using Prism 9 (GraphPad, San Diego). The results are presented as mean ± SEM. Unpaired Student’s *t*‐test was used to compare two parametric sample populations. For the comparison with chance, one group *t*‐test was used. Qualitative parameters (nesting) were analyzed using χ^2^ test. For more than two populations, a one‐way ANOVA and Tukey’s multiple comparisons test was performed. For grouped datasets, ordinary two‐way ANOVA or multiple *t*‐tests using the Holm–Sidak method was used, assuming sample populations have the same scatter (SD). The significance level of α = 0.05 was accepted (*P* < 0.05 *, < 0.01 **, < 0.001 ***).

## Author contributions

**Karima Habbas:** Validation; Investigation; Visualization; Methodology; Writing—original draft. **Oktay Cakil:** Formal analysis; Investigation; Methodology; Writing—original draft. **Boglárka Zámbó:** Conceptualization; Formal analysis; Validation; Investigation; Visualization; Methodology; Writing—original draft; Writing—review & editing. **Ricardos Tabet:** Conceptualization; Writing—original draft. **Fabrice Riet:** Conceptualization; Formal analysis; Validation; Methodology. **Doulaye Dembele:** Formal analysis; Methodology. **Jean‐Louis Mandel:** Conceptualization. **Michaël Hocquemiller:** Conceptualization; Methodology; Writing—original draft; Writing—review & editing. **Ralph Laufer:** Conceptualization; Methodology; Writing—original draft; Writing—review & editing. **Françoise Piguet:** Conceptualization; Resources; Methodology. **Hervé Moine:** Conceptualization; Data curation; Formal analysis; Supervision; Funding acquisition; Writing—original draft; Project administration; Writing—review & editing.

In addition to the CRediT author contributions listed above, the contributions in detail are:

KH, OC, BZ, FR, and FP designed and performed experiments and helped writing the manuscript. DD performed statistical analyses. RT, J‐LM, MH, RL helped designing the experiments and writing manuscript. HM supervised the project, designed experiments, and wrote the manuscript.

## Disclosure and competing interests statement

HM and RT are listed as inventors on a patent describing the AAV construct reported in this manuscript. M.H. and R.L. are full‐time employees and hold equity in Lysogene. All other authors declare no competing interests.

## Supporting information



Appendix

Expanded View Figures PDF

Source Data for Expanded View and Appendix

Source Data for Figure 1

Source Data for Figure 2

Source Data for Figure 3

Source Data for Figure 4

## Data Availability

This study includes no data deposited in external repositories.

## References

[emmm202114649-bib-0030] Avila‐Flores A , Santos T , Rincon E , Merida I (2005) Modulation of the mammalian target of rapamycin pathway by diacylglycerol kinase‐produced phosphatidic acid. J Biol Chem 280: 10091–10099 15632115 10.1074/jbc.M412296200

[emmm202114649-bib-0001] Bear MF , Huber KM , Warren ST (2004) The mGluR theory of fragile X mental retardation. Trends Neurosci 27: 370–377 15219735 10.1016/j.tins.2004.04.009

[emmm202114649-bib-0002] Chan KY , Jang MJ , Yoo BB , Greenbaum A , Ravi N , Wu W‐L , Sánchez‐Guardado L , Lois C , Mazmanian SK , Deverman BE *et al* (2017) Engineered AAVs for efficient noninvasive gene delivery to the central and peripheral nervous systems. Nat Neurosci 20: 1172–1179 28671695 10.1038/nn.4593 PMC5529245

[emmm202114649-bib-0003] Dolen G , Osterweil E , Rao BS , Smith GB , Auerbach BD , Chattarji S , Bear MF (2007) Correction of fragile X syndrome in mice. Neuron 56: 955–962 18093519 10.1016/j.neuron.2007.12.001 PMC2199268

[emmm202114649-bib-0004] Gantois I , Khoutorsky A , Popic J , Aguilar‐Valles A , Freemantle E , Cao R , Sharma V , Pooters T , Nagpal A , Skalecka A *et al* (2017) Metformin ameliorates core deficits in a mouse model of fragile X syndrome. Nat Med 23: 674–677 28504725 10.1038/nm.4335

[emmm202114649-bib-0005] Gaskill BN , Karas AZ , Garner JP , Pritchett‐Corning KR (2013) Nest building as an indicator of health and welfare in laboratory mice. J Vis Exp 82: 51012 10.3791/51012 PMC4108067 24429701

[emmm202114649-bib-0006] Gholizadeh S , Arsenault J , Xuan IC , Pacey LK , Hampson DR (2014) Reduced phenotypic severity following adeno‐associated virus‐mediated Fmr1 gene delivery in fragile X mice. Neuropsychopharmacology 39: 3100–3111 24998620 10.1038/npp.2014.167 PMC4229583

[emmm202114649-bib-0007] Gkogkas C , Khoutorsky A , Cao R , Jafarnejad S , Prager‐Khoutorsky M , Giannakas N , Kaminari A , Fragkouli A , Nader K , Price T *et al* (2014) Pharmacogenetic inhibition of eIF4E‐dependent Mmp9 mRNA translation reverses fragile X syndrome‐like phenotypes. Cell Rep 9: 1742–1755 25466251 10.1016/j.celrep.2014.10.064 PMC4294557

[emmm202114649-bib-0008] Gross C , Chang C‐W , Kelly S , Bhattacharya A , McBride S , Danielson S , Jiang M , Chan C , Ye K , Gibson J *et al* (2015) Increased expression of the PI3K enhancer PIKE mediates deficits in synaptic plasticity and behavior in fragile X syndrome. Cell Rep 11: 727–736 25921541 10.1016/j.celrep.2015.03.060 PMC4418204

[emmm202114649-bib-0009] Guo W , Murthy AC , Zhang L , Johnson EB , Schaller EG , Allan AM , Zhao X (2012) Inhibition of GSK3beta improves hippocampus‐dependent learning and rescues neurogenesis in a mouse model of fragile X syndrome. Hum Mol Genet 21: 681–691 22048960 10.1093/hmg/ddr501 PMC3259018

[emmm202114649-bib-0010] Hagerman RJ , Berry‐Kravis E , Hazlett HC , Bailey DB , Moine H , Kooy RF , Tassone F , Gantois I , Sonenberg N , Mandel JL *et al* (2017) Fragile X syndrome. Nat Rev Dis Primers 3: 17065 28960184 10.1038/nrdp.2017.65

[emmm202114649-bib-0011] Hampson DR , Hooper AWM , Niibori Y (2019) The application of adeno‐associated viral vector gene therapy to the treatment of fragile X syndrome. Brain Sci 9: 32 10.3390/brainsci9020032 PMC6406794 30717399

[emmm202114649-bib-0033] Hatcher JP , Jones DN , Rogers DC , Hatcher PD , Reavill C , Hagan JJ , Hunter AJ (2001) Development of SHIRPA to characterise the phenotype of gene‐targeted mice. Behav Brain Res 125: 43–47 11682092 10.1016/s0166-4328(01)00275-3

[emmm202114649-bib-0012] Herzog R , Schwudke D , Schuhmann K , Sampaio JL , Bornstein SR , Schroeder M , Shevchenko A (2011) A novel informatics concept for high‐throughput shotgun lipidomics based on the molecular fragmentation query language. Genome Biol 12: R8 21247462 10.1186/gb-2011-12-1-r8 PMC3091306

[emmm202114649-bib-0035] Hu J , Li X , Coleman K , Schroeder A , Ma N , Irwin DJ , Lee EB , Shinohara RT , Li M (2021) SpaGCN: integrating gene expression, spatial location and histology to identify spatial domains and spatially variable genes by graph convolutional network. Nat Methods 18: 1342–1351 34711970 10.1038/s41592-021-01255-8

[emmm202114649-bib-0013] Imai S , Kai M , Yasuda S , Kanoh H , Sakane F (2005) Identification and characterization of a novel human type II diacylglycerol kinase, DGK kappa. J Biol Chem 280: 39870–39881 16210324 10.1074/jbc.M500669200

[emmm202114649-bib-0014] Jiang Y , Sakane F , Kanoh H , Walsh JP (2000) Selectivity of the diacylglycerol kinase inhibitor 3‐[2‐(4‐[bis‐(4‐fluorophenyl)methylene]‐1‐piperidinyl)ethyl]‐2, 3‐dihydro‐2‐thioxo‐4(1H)quinazolinone (R59949) among diacylglycerol kinase subtypes. Biochem Pharmacol 59: 763–772 10718334 10.1016/s0006-2952(99)00395-0

[emmm202114649-bib-0015] Kaufmann WE , Cortell R , Kau AS , Bukelis I , Tierney E , Gray RM , Cox C , Capone GT , Stanard P (2004) Autism spectrum disorder in fragile X syndrome: communication, social interaction, and specific behaviors. Am J Med Genet A 129A: 225–234 15326621 10.1002/ajmg.a.30229

[emmm202114649-bib-0034] Leboucher A , Bermudez‐Martin P , Mouska X , Amri E‐Z , Pisani DF , Davidovic L (2019) Fmr1‐deficiency impacts body composition, skeleton, and bone microstructure in a mouse model of fragile X syndrome. Front Endocrinol (Lausanne) 10: 678 31632352 10.3389/fendo.2019.00678 PMC6783488

[emmm202114649-bib-0031] Maurin T , Lebrigand K , Castagnola S , Paquet A , Jarjat M , Popa A , Grossi M , Rage F , Bardoni B (2018) HITS‐CLIP in various brain areas reveals new targets and new modalities of RNA binding by fragile X mental retardation protein. Nucleic Acids Res 46: 6344–6355 29668986 10.1093/nar/gky267 PMC6158598

[emmm202114649-bib-0016] Mazroui R , Huot ME , Tremblay S , Filion C , Labelle Y , Khandjian EW (2002) Trapping of messenger RNA by Fragile X mental retardation protein into cytoplasmic granules induces translation repression. Hum Mol Genet 11: 3007–3017 12417522 10.1093/hmg/11.24.3007

[emmm202114649-bib-0017] Mientjes EJ , Nieuwenhuizen I , Kirkpatrick L , Zu T , Hoogeveen‐Westerveld M , Severijnen L , Rife M , Willemsen R , Nelson DL , Oostra BA (2006) The generation of a conditional Fmr1 knock out mouse model to study Fmrp function *in vivo* . Neurobiol Dis 21: 549–555 16257225 10.1016/j.nbd.2005.08.019

[emmm202114649-bib-0038] Ouagazzal A‐M , Moreau J‐L , Pauly‐Evers M , Jenck F (2003) Impact of environmental housing conditions on the emotional responses of mice deficient for nociceptin/orphanin FQ peptide precursor gene. Behav Brain Res 144: 111–117 12946601 10.1016/s0166-4328(03)00066-4

[emmm202114649-bib-0018] Pasciuto E , Ahmed T , Wahle T , Gardoni F , D’Andrea L , Pacini L , Jacquemont S , Tassone F , Balschun D , Dotti C *et al* (2015) Dysregulated ADAM10‐mediated processing of APP during a critical time window leads to synaptic deficits in fragile X syndrome. Neuron 87: 382–398 26182420 10.1016/j.neuron.2015.06.032

[emmm202114649-bib-0032] Paxinos G , Franklin KBJ (2001) The mouse brain in stereotaxic coordinates, 2^nd^ edn. San Diego: Academic Press

[emmm202114649-bib-0019] Qin M , Kang J , Burlin TV , Jiang C , Smith CB (2005) Postadolescent changes in regional cerebral protein synthesis: an in vivo study in the FMR1 null mouse. J Neurosci 25: 5087–5095 15901791 10.1523/JNEUROSCI.0093-05.2005 PMC6724856

[emmm202114649-bib-0020] Sampaio JL , Gerl MJ , Klose C , Ejsing CS , Beug H , Simons K , Shevchenko A (2011) Membrane lipidome of an epithelial cell line. Proc Natl Acad Sci USA 108: 1903–1907 21245337 10.1073/pnas.1019267108 PMC3033259

[emmm202114649-bib-0037] Santos AR , Kanellopoulos AK , Bagni C (2014) Learning and behavioral deficits associated with the absence of the fragile X mental retardation protein: what a fly and mouse model can teach us. Learn Mem 21: 543–555 25227249 10.1101/lm.035956.114 PMC4175497

[emmm202114649-bib-0021] Sidhu H , Dansie LE , Hickmott PW , Ethell DW , Ethell IM (2014) Genetic removal of matrix metalloproteinase 9 rescues the symptoms of fragile X syndrome in a mouse model. J Neurosci 34: 9867–9879 25057190 10.1523/JNEUROSCI.1162-14.2014 PMC4107404

[emmm202114649-bib-0022] Sonenberg N (1994) Regulation of translation and cell growth by eIF‐4E. Biochimie 76: 839–846 7880900 10.1016/0300-9084(94)90185-6

[emmm202114649-bib-0023] Tabet R , Moutin E , Becker JAJ , Heintz D , Fouillen L , Flatter E , Krężel W , Alunni V , Koebel P , Dembélé D *et al* (2016a) Fragile X mental retardation protein (FMRP) controls diacylglycerol kinase activity in neurons. Proc Natl Acad Sci USA 113: E3619–3628 27233938 10.1073/pnas.1522631113 PMC4932937

[emmm202114649-bib-0024] Tabet R , Vitale N , Moine H (2016b) Fragile X syndrome: Are signaling lipids the missing culprits? Biochimie 130: 188–194 27597551 10.1016/j.biochi.2016.09.002

[emmm202114649-bib-0025] The Dutch‐Belgian Fragile X Consortium (1994) Fmr1 knockout mice: a model to study fragile X mental retardation. Cell 78: 23–33 8033209

[emmm202114649-bib-0036] de Vries BB , Robinson H , Stolte‐Dijkstra I , Tjon Pian Gi CV , Dijkstra PF , van Doorn J , Halley DJ , Oostra BA , Turner G , Niermeijer MF (1995) General overgrowth in the fragile X syndrome: variability in the phenotypic expression of the FMR1 gene mutation. J Med Genet 32: 764–769 8558551 10.1136/jmg.32.10.764 PMC1051696

[emmm202114649-bib-0026] Wang X , Flynn A , Waskiewicz AJ , Webb BL , Vries RG , Baines IA , Cooper JA , Proud CG (1998) The phosphorylation of eukaryotic initiation factor eIF4E in response to phorbol esters, cell stresses, and cytokines is mediated by distinct MAP kinase pathways. J Biol Chem 273: 9373–9377 9545260 10.1074/jbc.273.16.9373

[emmm202114649-bib-0027] Westmark CJ , Westmark PR , O'Riordan KJ , Ray BC , Hervey CM , Salamat MS , Abozeid SH , Stein KM , Stodola LA , Tranfaglia M *et al* (2011) Reversal of fragile X phenotypes by manipulation of AbetaPP/Abeta levels in Fmr1KO mice. PLoS One 6: e26549 22046307 10.1371/journal.pone.0026549 PMC3202540

[emmm202114649-bib-0028] Yamasue H , Aran A , Berry‐Kravis E (2019) Emerging pharmacological therapies in fragile X syndrome and autism. Curr Opin Neurol 32: 635–640 31045620 10.1097/WCO.0000000000000703

[emmm202114649-bib-0029] Zolotukhin S , Potter M , Zolotukhin I , Sakai Y , Loiler S , Fraites TJ , Chiodo VA , Phillipsberg T , Muzyczka N , Hauswirth WW *et al* (2002) Production and purification of serotype 1, 2, and 5 recombinant adeno‐associated viral vectors. Methods 28: 158–167 12413414 10.1016/s1046-2023(02)00220-7

