# Supplementary material for: AAV‐delivered diacylglycerol kinase DGKk achieves long‐term rescue of fragile X syndrome mouse model
Source: EMBO Mol Med. 2022 Apr 4;14(5):e14649. doi: 10.15252/emmm.202114649 (PMC9081908; doi:10.15252/emmm.202114649)
Supplement: Supplementary file 1 — Appendix [file EMMM-14-e14649-s008.docx]

**Appendix**

**Title: AAV-delivered diacylglycerol kinase DGKk achieves long-term rescue of fragile X syndrome mouse model**

**Authors:** Karima Habbas^1†^, Oktay Cakil^1†^, Boglarka Zambo^1†^, Ricardos Tabet^1‡^, Fabrice Riet^2^, Doulaye Dembele^1^, Jean-Louis Mandel^1^, Michaël Hocquemiller^3^, Ralph Laufer^3^, Françoise Piguet^4^, Hervé Moine^1*^

Correspondence to [moine@igbmc.fr](mailto:moine@igbmc.fr)

Content:

**Appendix Figure S1 to S11**

**Appendix** **Table S1 to S3**

**Appendix Figure S1 : ∆N-DGKk expression normalizes the level of various individual phosphatidic acid species altered in *Fmr1*-KO** **cortex.**

(**A-C**) Measure of individual PA species level by mass spectrometry in cortex (**A**), hippocampus (**B**) and rest of brain (**C**) of WT mice treated with saline solution (WT) and *Fmr1*-KO mice treated with saline (Fmr1-S), AAVPHP.eB-∆N-DGKk (Fmr1-PHP.eB), AAVRh10-∆N-DGKk (Fmr1-Rh10) 8 weeks after injections. n=8 individual animals.

Data information: Data are expressed as mean ± SEM. The variances do not vary significantly (Fisher-Snedecor variance test) for the four groups of an analysis. Hence, for each condition-tissue data, separate ANOVA tests were performed. An analysis is significant if the test p-value is less or equal to alpha=0.05. Post-hoc tests were done using the Student-Newman-Keuls method for significant analyses and the corrected p-value level used was alpha/3. **P* < 0.017, ***P* < 0.0034, ****P* < 0.00034 vs WT-S, #*P* < 0.017, ##*P* < 0.0034 vs Fmr1-S.

**Appendix Figure S2 : Measure of individual diacylglycerol species level.**

(**A-C**) Measure of individual DAG species level by mass spectrometry in cortex (**A**), hippocampus (**B**) and rest of brain (**C**) of WT mice treated with saline solution (WT) and *Fmr1*-KO mice treated with saline (Fmr1-S), AAVPHP.eB-∆N-DGKk (Fmr1-PHP.eB), AAVRh10-∆N-DGKk (Fmr1-Rh10) 8 weeks after injections. n=8 individual animals.

Data information: Data are expressed as mean ± SEM. The variances do not vary significantly (Fisher-Snedecor variance test) for the four groups of an analysis. Hence, for each condition-tissue data, separate ANOVA tests were performed. An analysis is significant if the test p-value is less or equal to alpha=0.05. Post-hoc tests were done using the Student-Newman-Keuls method for significant analyses and the corrected p-value level used was alpha/3. **P* < 0.017 vs WT-S, #*P* < 0.017, ####*P* < 0.000034 vs Fmr1-S.

**Appendix Figure S3 : Genotype and treatment does not affect global brain lipid composition**.

Mol % of total lipid for cholesterol esters (CE), diacylglycerol (DAG), phosphatidic acid (PA), phosphatidylcholine (PC), phosphatidylethanolamine (PE), phosphatidylglycerol (PG), phosphatidylinositol (PI), phosphatidylserine (PS), sphingomyelin (SM), triacylglycerol (TAG), n=8 individual animals, measured as in Fig EV4.

Data information: Data are expressed as mean ± SEM. Statistical analysis as in Fig. S2.

**Appendix Figure S4 :** **AAVRh10-∆N-DGKk rescues behavior alterations of *Fmr1*-KO mouse, 4 weeks after its administration.** Circadian activity analysis of locomotor (**A**) and rearing (**B**) activity) per hour over the 32h testing (upper panel) and total locomotor and rearing activity for the habituation, dark and light phases (mid panel) and for the first habituation hour, first dark hour and total duration (lower panel).

Data information: Data are expressed as mean ± SEM for upper panel and as median with interquartile range with minimum and maximum values for the other panels. Statistical analysis: one-way ANOVA and Tukey’s multiple comparisons test. n=24 mice per group. **P* < 0.05.

**Appendix Figure S5 :** **AAVRh10-∆N-DGKk rescues behavior alterations of *Fmr1*-KO mouse, 4 weeks after its administration.**

(**A**) Novel object recognition in 50 cm diameter arena (30cm height). Duration of objects exploration during the acquisition and retention trials.

(**B**) Novel object recognition in 30 cm diameter arena (30 cm height). Locomotor activity (distance) in the whole arena during the 15min habituation, acquisition and retention trials. Duration of objects exploration during the acquisition and retention trials and recognition index.

**(C)** Digging, marble burying and grooming duration tests. Data are expressed as median with interquartile range with minimum and maximum values.

Data information: Data are expressed as median with interquartile range with minimum and maximum values. Statistical analysis: one-way ANOVA and Tukey’s multiple comparisons test. n=24 mice per group. **P* < 0.05, ***P* < 0.01; one group t-test, @@@@ *P* < 0.0001 vs chance (50%).

**Appendix Figure S6 :** **AAVRh10-∆N-DGKk rescues behavior alterations of *Fmr1*-KO mouse, 4 weeks after its administration.**

Social recognition test. Number of entries and locomotor activity (total traveled distance in cm) in the two side compartments during habituation (up), social preference (middle) and social memory (bottom) sessions. Social preference was determined as percentage of exploration of a congener vs an object (middle right) and social memory as percentage of exploration of a novel vs familiar congener (bottom right).

Data information: Data are expressed as median with interquartile range with minimum and maximum values. Statistical analysis: one-way ANOVA and Tukey’s multiple comparisons test. n=24 mice per group. **P <* 0.05, ***P* < 0.01; one group t-test, @ *P* < 0.05, @@@@ *P* < 0.0001 vs chance (50%).

**Appendix Figure S7 :** **AAVRh10-∆N-DGKk rescues behavior alterations of *Fmr1*-KO mouse, 4 weeks after its administration.**

Nest building test. Scoring of nests at 2, 5 and 24h. 0-5 scale as described by Gaskill et al (2013): 0 = undisturbed nesting material; 1 = disturbed nesting material but no nest site; 2 = a flat nest without walls; 3 = a cup nest with a wall less than ½ the height of a dome that would cover a mouse; 4 = an incomplete dome with a wall ½ the height of a dome; 5 = a complete dome with walls taller than ½ the height of a dome, which may or may not fully enclose the nest.

Data information: Data are expressed as median with interquartile range with minimum and maximum values. Statistical analysis: χ2 test (n=12). **P* < 0.05.

**Appendix Figure S8 :** **Behavioral analyses of AAVRh10-∆N-DGKk treated *Fmr1*-KO mice (8 weeks after injection).**

(**A**,**B**) Circadian activity analysis of locomotor (**A**) and rearing (**B**) activity per hour over the 32h testing (upper panel) and total locomotor and rearing activity for the habituation, dark and light phases (mid panel) and for the first habituation hour, first dark hour and total duration (lower panel).

Data information: Data are expressed as mean ± SEM for upper panels of A and B, and as median with interquartile range with minimum and maximum values for other panels. Statistical analysis: one-way ANOVA with Tukey’s multiple comparisons test (n=12) **P* < 0.05, ***P* < 0.01.

**Appendix Figure S9 :** **Behavioral analyses of AAVRh10-∆N-DGKk treated *Fmr1*-KO mice (8 weeks after injection).**

(**A**) Elevated Plus Maze. Percentage of time spent in open arms and number of entries in open, closed and total (open+closed) arms.

(**B**) Novel object recognition in 50cm diameter arena (30cm height). Percentage of time spent in the center during the habituation, locomotor activity (distance) in the whole arena during the habituation, acquisition and retention trials. Duration of objects exploration during the acquisition and retention trials and recognition index.

(**C**) Novel object recognition in 30cm diameter arena (30cm height). Locomotor activity (distance) in the whole arena during the habituation and retention trials. Duration of objects exploration during the acquisition trials and recognition index.

Data information: Data are expressed as median with interquartile range with minimum and maximum values. Statistical analysis: one-way ANOVA with Tukey’s multiple comparisons test (n=12) **P* < 0.05, ***P* < 0.01, ****P* < 0.001, *****P* < 0.0001 and one group t-test @*P* < 0.05, @@*P* < 0.01, @@@*P* < 0.001 vs chance (50%).


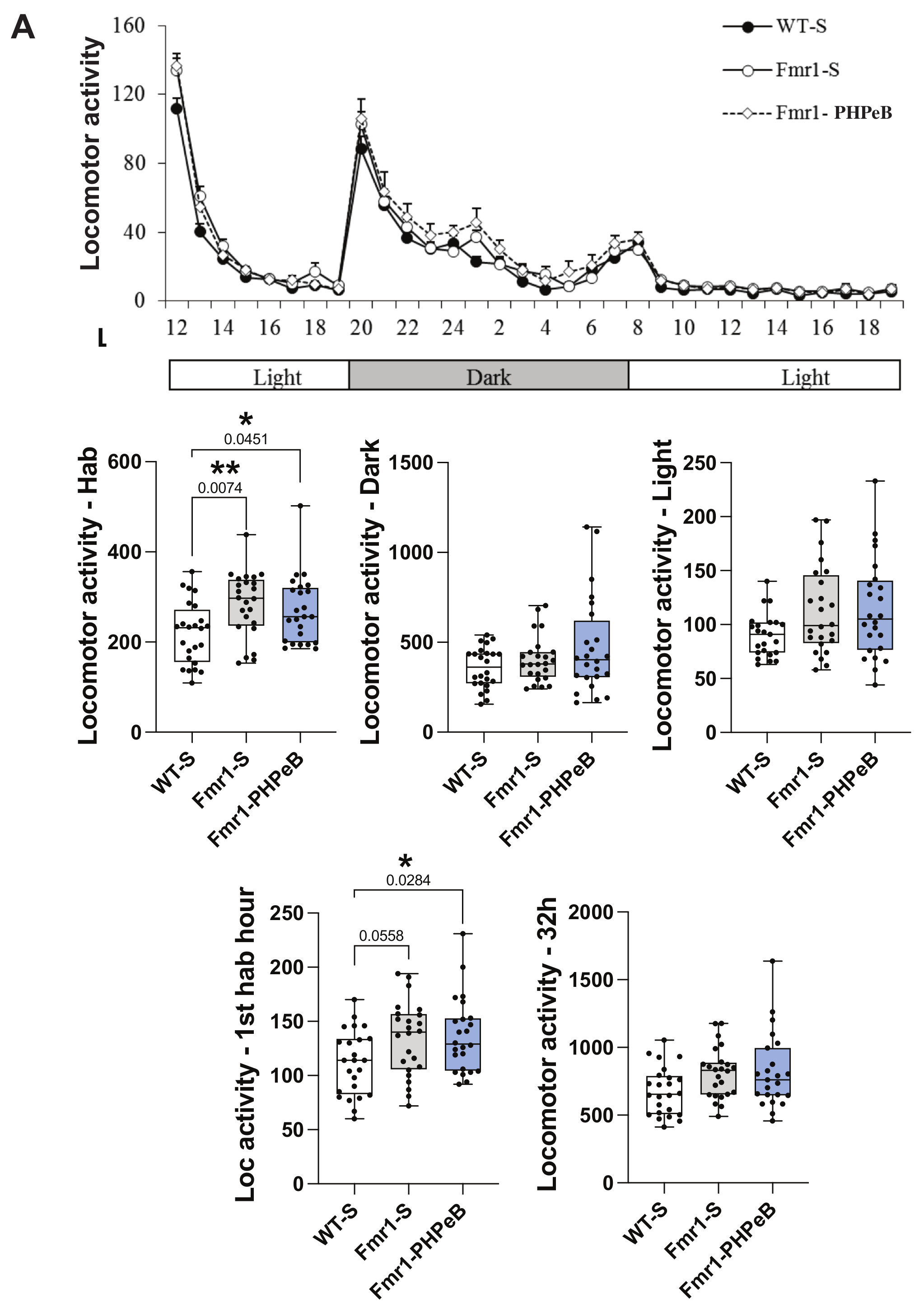


**
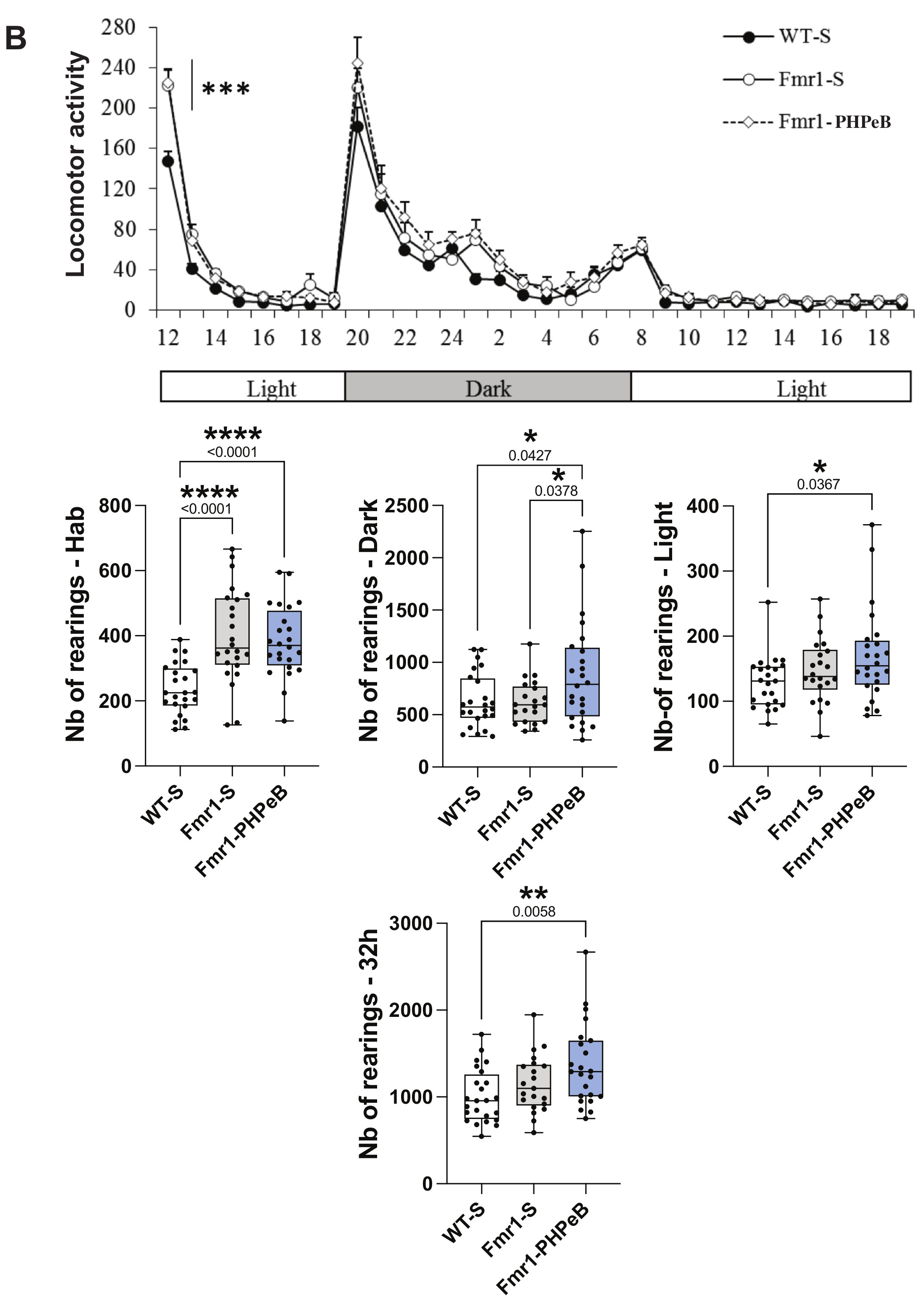
**

**
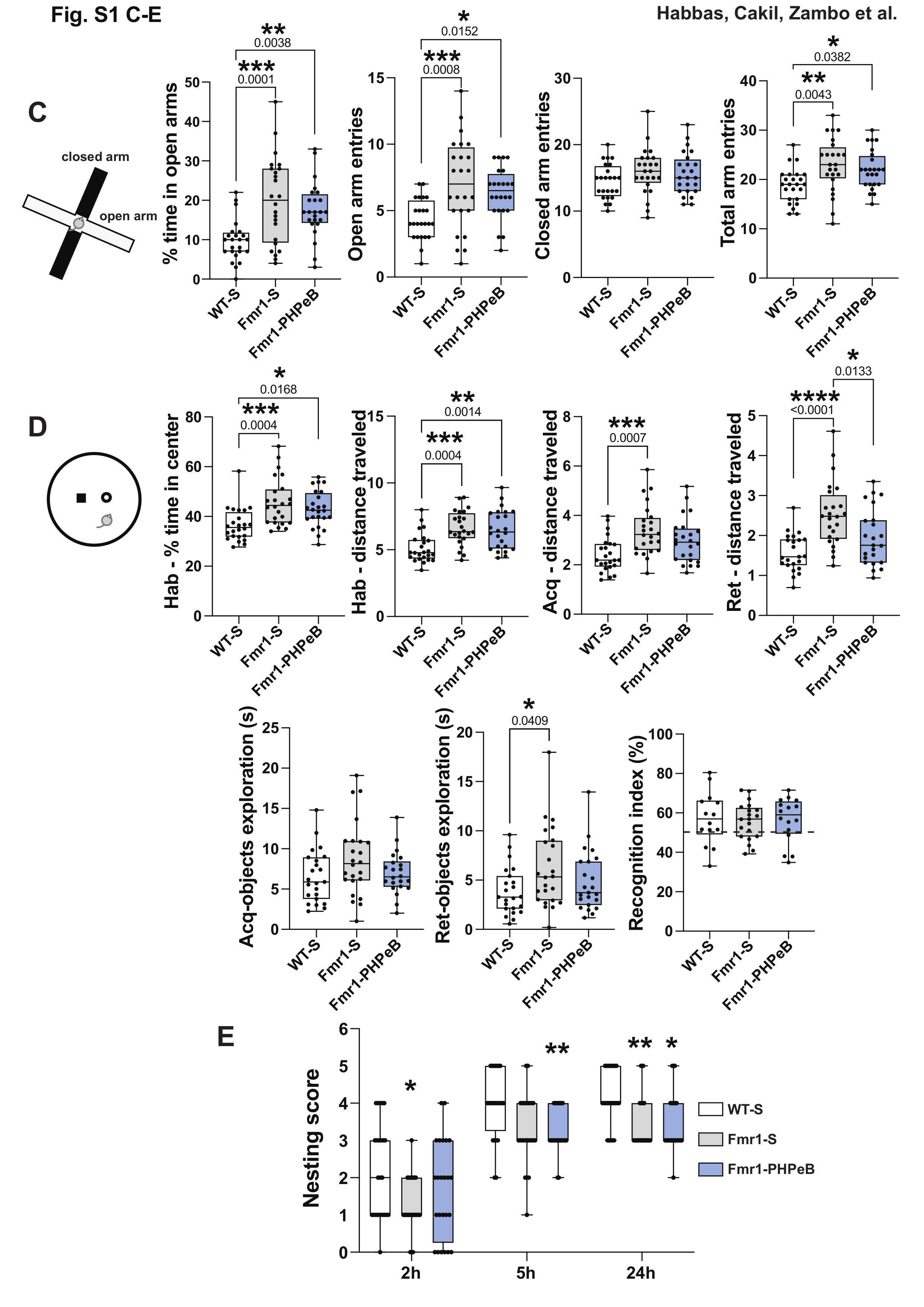
**

**
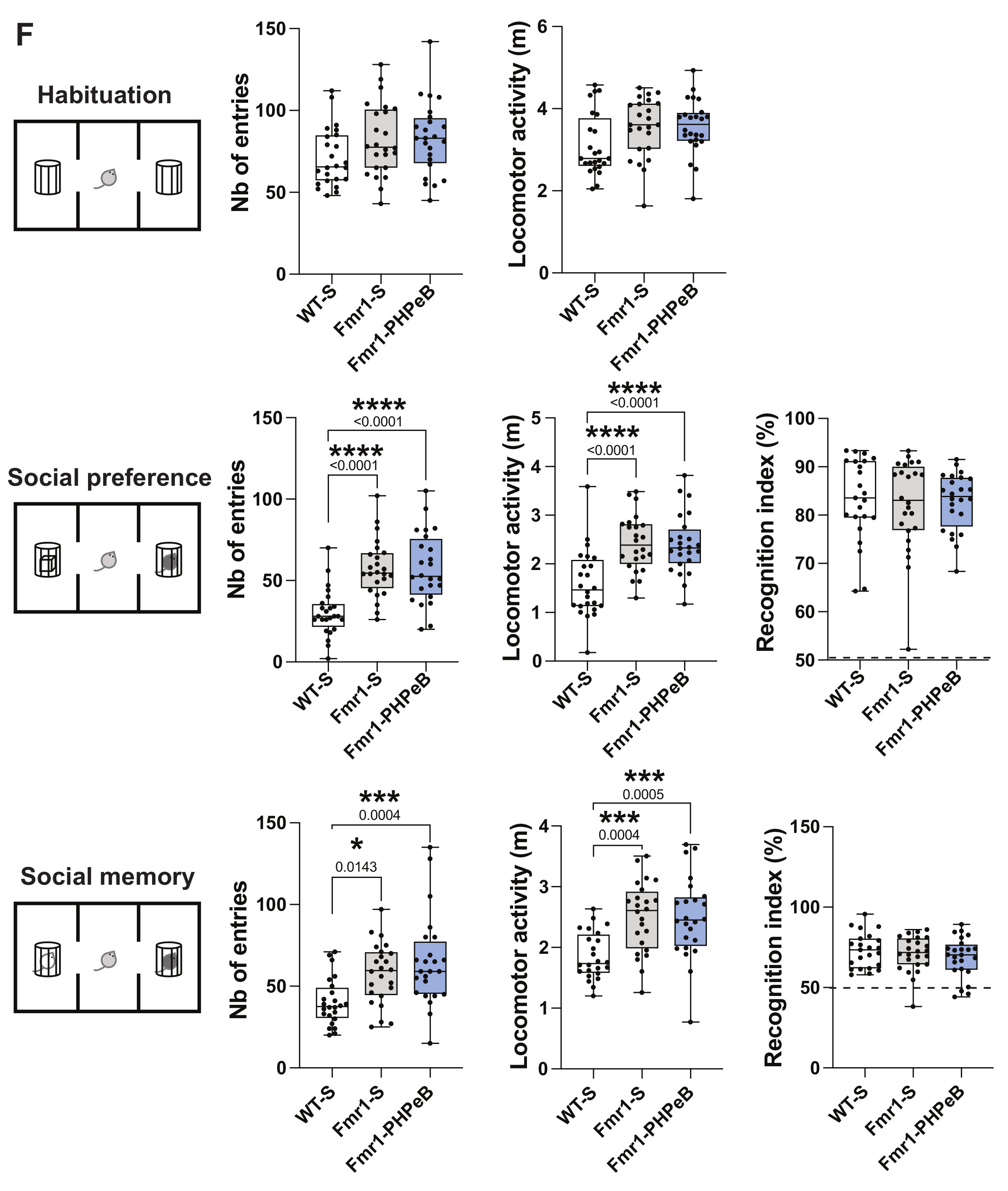
**

**Appendix Figure S10: Behavioral analyses of AAVPHP.eB-∆N-DGKk treated *Fmr1*-KO mice (4 weeks after injection).** (**A**,**B**) Circadian activity analysis of locomotor (A) and rearing (B) activity per hour over the 32h testing (upper panel) and total locomotor and rearing activity for the habituation, dark and light phases (mid panel) and for the first habituation hour, first dark hour and total duration (lower panel). (**C)** Elevated Plus Maze. Percentage of time spent in open arms and number of entries in open, closed and total (open+closed) arms. (**D**) Novel object recognition in 50cm diameter arena (30cm height). Percentage of time spent in the center during the habituation, locomotor activity (distance) in the whole arena during the habituation, acquisition and retention trials. Duration of objects exploration during the acquisition and retention trials and recognition index. (**E**) Nest building. Scoring of nests at 2, 5 and 24h as in Fig. EV4H. (**F**) Social recognition. Number of entries and locomotor activity in the two side compartments during habituation (up), social preference (middle), social memory (bottom) sessions. Social preference was determined as percentage of exploration of a congener vs an object (middle right) and social memory as percentage of exploration of a novel vs familiar congener (bottom right).

Data information: Data are expressed as mean ± SEM for upper panels of A and B, and for E and as median with interquartile range with minimum and maximum values for other panels. Statistical analyses: one-way ANOVA, Tukey’s multiple comparisons test and one group t-test (recognition index) or χ2 test (nesting). **P* < 0.05, ***P* < 0.01, ****P* < 0.001, *****P* < 0.0001.

**
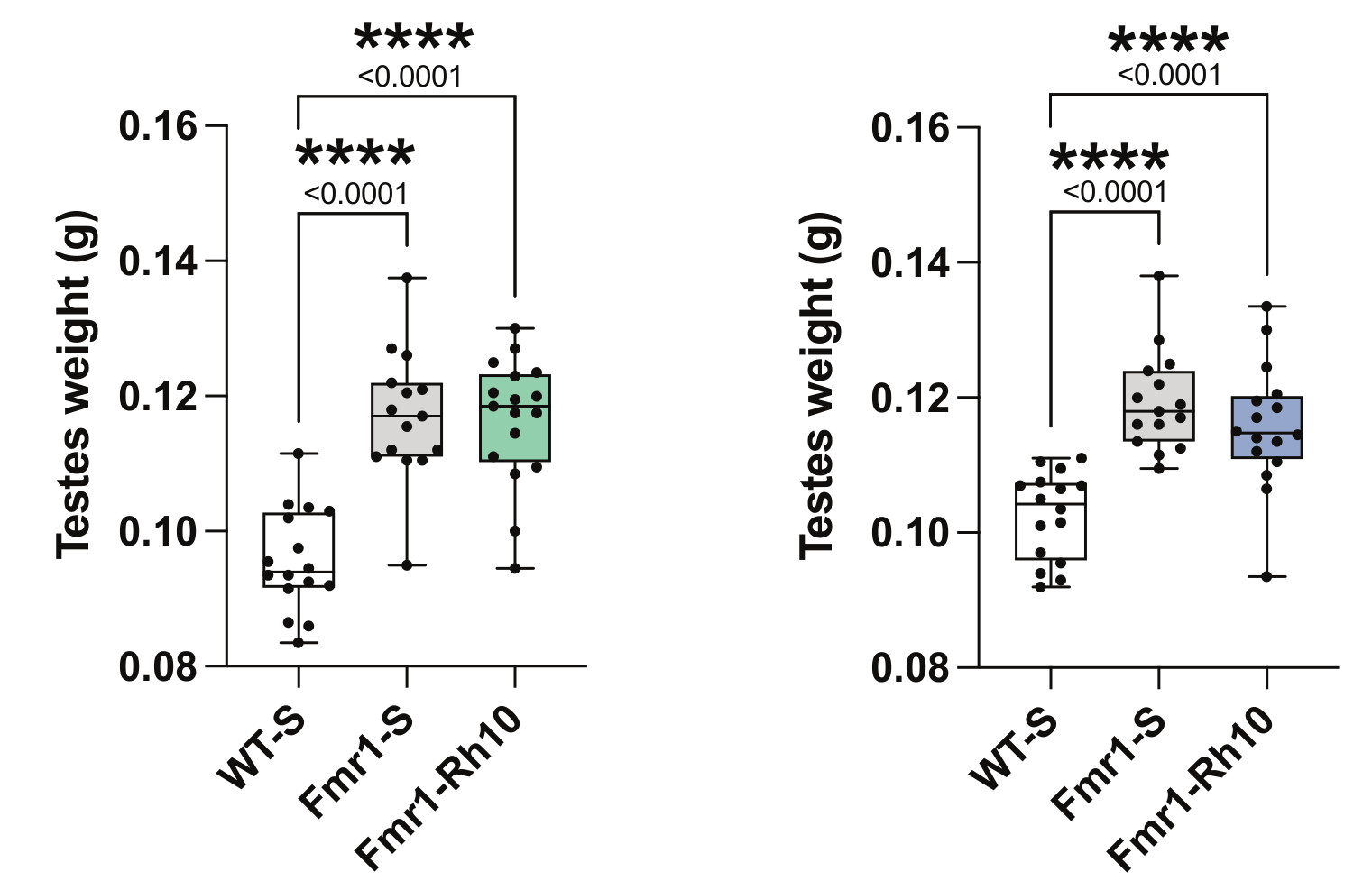
**

**Appendix Figure S11**: **Testes weight of** **AAVRh10-∆N-DGKk (left) and AAVPHP.eB-∆N-DGKk (right) treated *Fmr1*-KO mice (12 weeks after injection)**.

Data are means of the two testes of each animal and expressed as median with interquartile range with minimum and maximum values and analyzed using one-way ANOVA, Tukey’s multiple comparisons test *****P* < 0.0001.

**Appendix** **Table S1:**

**Clinical observations of Rh10-∆N-DGKk treated WT mice (n=10) using modified SHIRPA tests, 4 weeks after injections (age 9 w).**

| **Parameter measured** | changes compared with control WT-saline |
| --- | --- |
| Body weight (g) | 0 |
| Temperature (°C) | 0 |
| Body position | 0 |
| Tremor | 0 |
| Palpebral closure | 0 |
| Coat appearance | 0 |
| Whiskers | 0 |
| Lacrimation | 0 |
| Defecation | 0 |
| Transfer arousal | 0 |
| Locomotor activity | 0 |
| Gait | 0 |
| Tail elevation | 0 |
| Vision | 0 |
| Olfaction | 0 |
| Startle response | 0 |
| Touch escape | 0 |
| Positional passivity | 0 |
| Skin colour | 0 |
| Trunk curl | 0 |
| Limb grasping | 0 |
| Pinna reflex | 0 |
| Corneal reflex | 0 |
| Righting reflex | 0 |
| Contact righting reflex | 0 |
| Evidence of biting | 0 |
| Vocalization | 0 |

**Appendix** **Table S2:**

**Summary of the phenotypic comparisons** **between WT mice treated saline (WT-S), *Fmr1*-KO treated saline (KO-S) and *Fmr1*-KO treated AAV (KO-Rh10 or KO-PhPeB) 4 and 8 weeks after treatment.** Statistical analyses: one-way ANOVA, Tukey’s multiple comparisons test and one group t-test (recognition index) or χ2 test (nesting), n=24 for 4 weeks, n=12 for 8 weeks. **P* <0.05, ***P < 0.01*, ****P <* 0.001, *****P* < 0.0001 KO-S vs WT-S, or KO-AAV vs WT-S. #*P* <0.05, ##*P* < 0.01, ###*P* < 0.001, ####*P* < 0.0001 KO-AAV vs KO.

**Appendix** **Table S3:**

**Antibodies used and working concentrations.**

| Table S3. Antibodies used and working concentrations. | | | | | | |
| --- | --- | --- | --- | --- | --- | --- |
| Antibody | Producer | Ref | Host | Clonality | Dilution | Working concentration |
| Anti-Fmrp | IGBMC | NA | Mouse | 1C3 | 1:10,000 | ascite |
| Anti-HA.11 | Biolegend | 901513 | Mouse | 16B12 | 1:5,000 | 0.2 µg/mL |
| Anti-HA | Roche | **11867423001** | Rat | 3F10 | 1:150 | 0.7 µg/mL |
| Anti-hDGKk | ThermoFisher | PA5-25046 | Rabbit | polyclonal | 1:1000 | Not available |
| anti-p-EIF4e (Ser209) | Cell Signaling | 9741 | Rabbit | polyclonal | 1:1000 | Not available |
| anti-EIF4E | Cell Signaling | 9742 | Rabbit | polyclonal | 1:1000 | 0.11 µg/mL |
| anti-puromycin | Sigma-Aldrich | **MABE343** | Mouse | 12D10 | 1:2000 | 0.5 µg/mL |
| anti-p-mTOR (Ser-2448) | Cell Signaling | 2971 | Rabbit | polyclonal | 1:1000 | 60 µg/mL |
| anti-mTOR | Cell Signaling | 2972 | Rabbit | polyclonal | 1:1000 | 33 µg/mL |
| anti-GAPDH | Chemicon | MAB374 | Mouse | 6C5 | 1:10,000 | 0.1 µg/mL |
| Anti-rabbit IgG HRP-conjugated | Jackson ImmunoResearch | 111-035-003 | Goat | polyclonal | 1:10,000 | 80 ng/mL |
| Anti-mouse IgG HRP-conjugated | Jackson ImmunoResearch | 115-035-146 | Goat | polyclonal | 1:10,000 | 80 ng/ml |
| anti-MAP2 | Millipore | AB5622 | Rabbit | polyclonal | 1:1000 | 8 µg/mL |
| anti-NeuN | Chemicon | MAB377 | Mouse | A60 | 1:500 | 2 µg/mL |
| rabbit anti-GFAP | Synaptic system | 173002 | Rabbit | polyclonal | 1:500 | Not available |
| anti-rabbit Alexa Fluor 594 or 488 | Invitrogen Corporation, Carlsbad, CA, USA | A-11012 and A-11008 | Goat | polyclonal | 1:1.000 | 2 µg/mL |
| Anti-mouse Alexa Fluor 488 or 596 | Invitrogen Corporation, Carlsbad, CA, USA | **A-21203 and A-21202** | Donkey | polyclonal | 1:1.000 | 2 µg/mL |
