# Supplementary material for: AAV‐delivered diacylglycerol kinase DGKk achieves long‐term rescue of fragile X syndrome mouse model
Source: EMBO Mol Med. 2022 Apr 4;14(5):e14649. doi: 10.15252/emmm.202114649 (PMC9081908; doi:10.15252/emmm.202114649)
Supplement: Supplementary file 2 — Expanded View Figures PDF [file EMMM-14-e14649-s005.pdf]

# Expanded View Figures

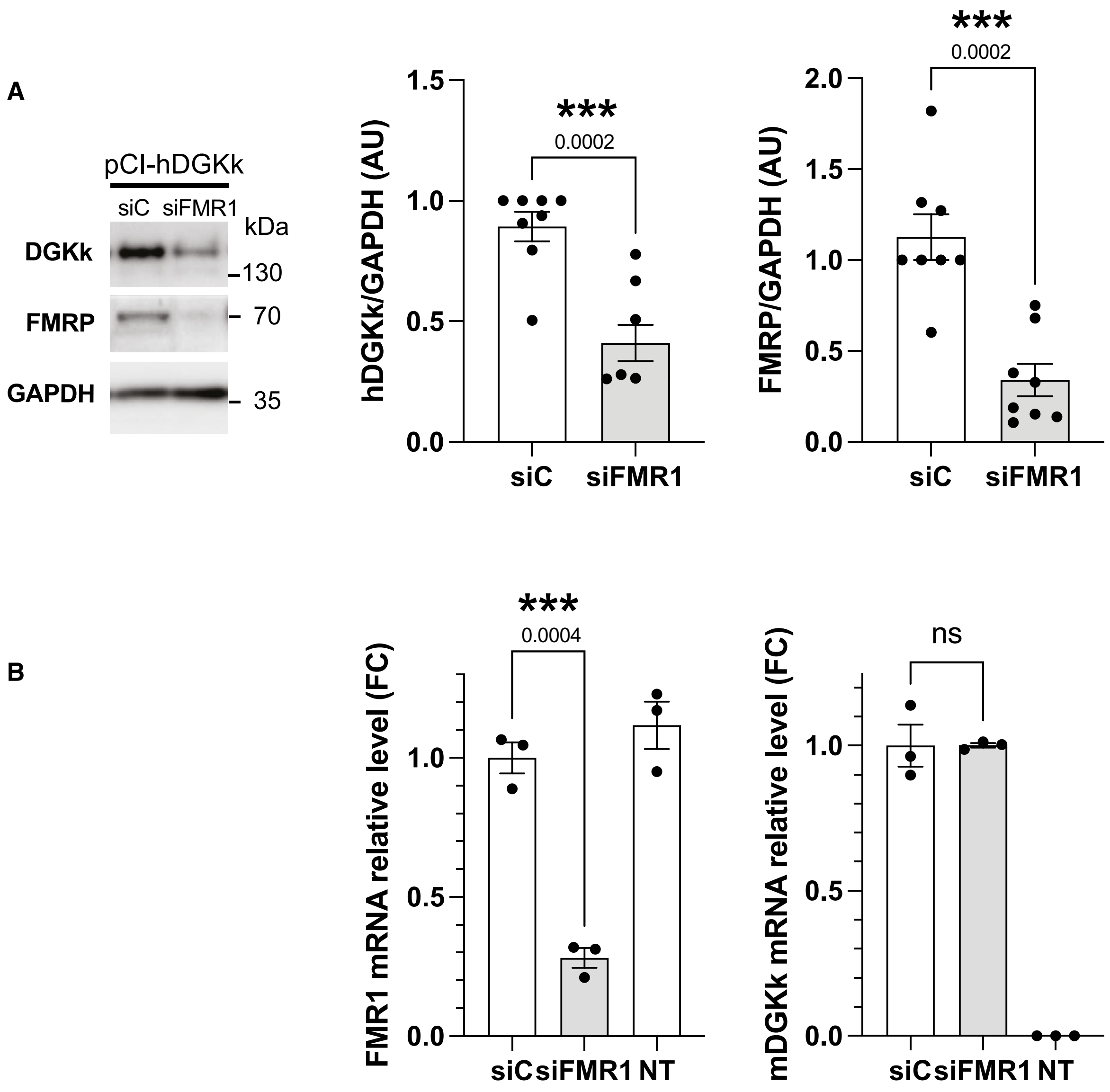


**Figure EV1. Influence of FMRP protein on human DGKk protein expression and on mouse DGKk mRNA level.**

A Immunoblots and quantification of lysates from Hela cells transfected with plasmid pCI-hDGKk encoding human DGKk and pre-transfected 24 h before with siRNA control (siC) or against FMRP (siFMR1). GAPDH was used as a loading control. For quantification, DGKk and FMRP signals were normalized against GAPDH signal and presented relative to the signal for siC-treated cells. Each point represents data from an individual culture, and all values are shown as mean ± SEM ***$P < 0.001$, calculated by unpaired Student *t*-test.

B Quantification of mouse DGKk mRNA by qRT-PCR in RNA extracts of Cos-1 cells transfected with plasmid pCI-mDGKκ-HA and pre-transfected with siRNA control (siC) or siRNA against FMRP (siFMR1) or mock transfected (NT). Each point represents data from an individual culture, and all values are means of fold change ± SEM, determined using ΔΔCt method with Actb as normalizer, $n = 3$ biological replicates. *P* values were calculated using one-way ANOVA with Tukey's multiple comparison test. ***$P < 0.001$; ns, $P > 0.05$.

Source data are available online for this figure.


© 2022 The Authors

**Figure EV2. ΔN-DGKk expression modulates mTOR and EIF4E signaling without significant toxicity.**

A Immunoblots and quantification of lysates from Cos-1 cells transfected with plasmid pCI control or pCI-HA-ΔN-DGKk, with the indicated amount of plasmid (μg) and after 24 h incubated with puromycin (20 μg/ml for 30 min) to measure basal rates of protein synthesis. GAPDH was used as a loading control. Densitogram of puromycin incorporation is presented as change relative to pCI (0.2 μg) condition.

B Cells transfected with 0.5 μg pCI, pCI-HA-FL-mDGKk, or pCI-HA-ΔN-mDGKk plasmids were serum starved for 24 h and then incubated with 10% FCS for the indicated time period. Cells were then collected and immunoblotted with HA (DGKk), p-mTOR, mTOR, p-EIF4E, or EIF4E antibodies. Blots were normalized for GAPDH and then phospho/non-phospho ratio was calculated. Upper panel: mDGKk expression. Middle panel: m-TOR phosphorylation at Ser-2448. Bottom panel: p-eIF4E phosphorylation at Ser-209. Significance was determined by 2-way ANOVA compared to pCI control ($n = 3$).

C–F Quantification of caspase 3/7 activity (C), release of lactate dehydrogenase (LDH) (D), percentage of NeunN (E), and GFAP positive cells (F), in WT and *Fmr1*-KO cortical neurons untreated (NT) or transduced at 8 DIV for 8 days with indicated titers of AAV (viral genome copies) AAVRh10-ΔN-DGKk or AAVRh10-FMRP by immunofluorescence high-throughput cell imaging. Positive control wells were treated with apoptotic inducer staurosporine (STS) at 0.1 and 1 μM for 6 h.

Data information: Data are mean ± SEM of individual cultures and analyzed using one-way ANOVA and Tukey's multiple comparisons test. *$P < 0.05$, ****$P < 0.0001$.
Source data are available online for this figure.

© 2022 The Authors

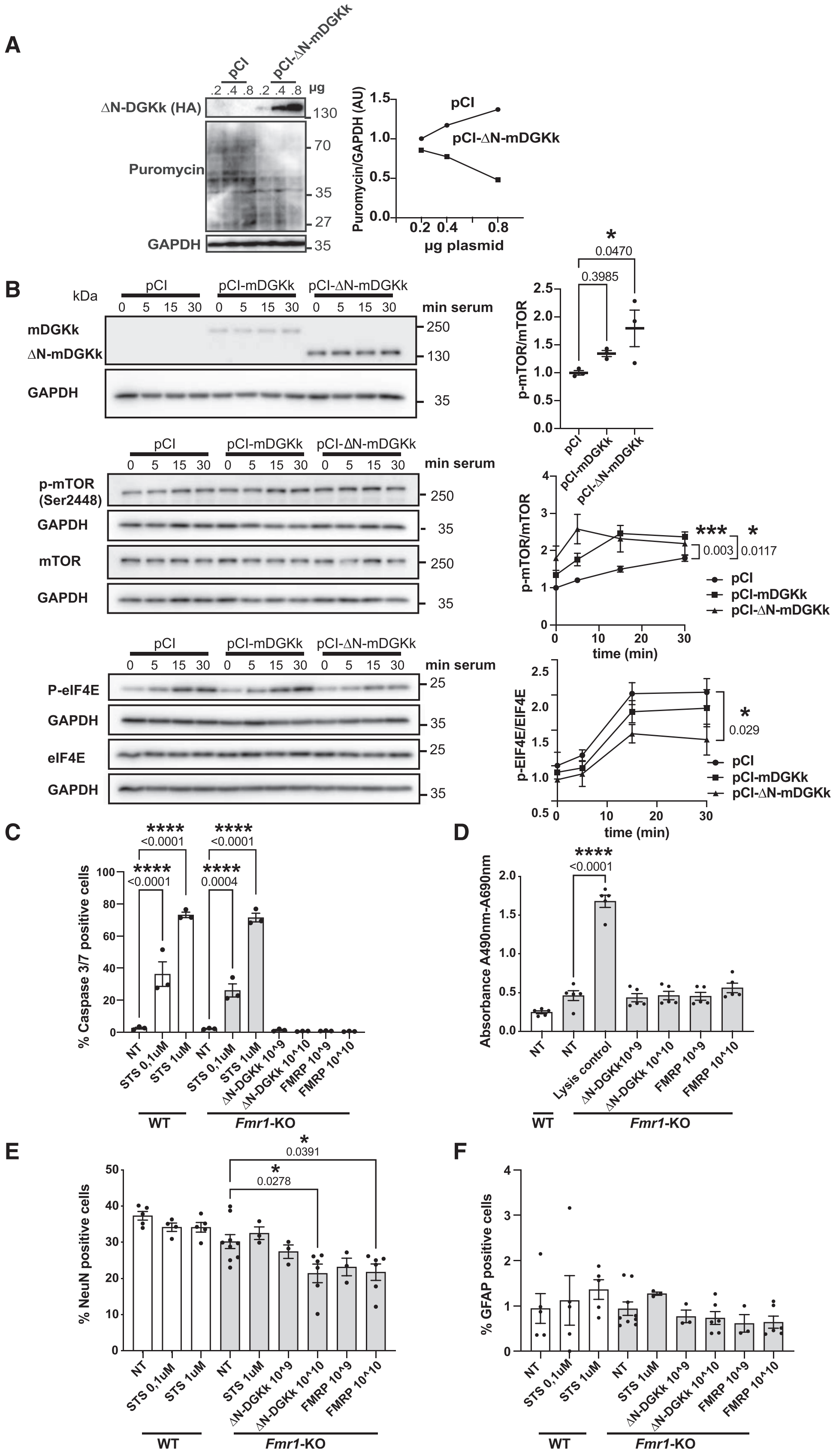


**Figure EV2.**

 © 2022 The Authors

**Figure EV3. ΔN-DGKk expression in brain with AAV vectors is stable over time and normalizes abnormal phosphatidic acid level in *Fmr1*-KO cortex.**

A Representative coronal brain sections processed for detection of ΔN-DGKk (HA) at 8 and 12 weeks post-injections using immunohistochemistry on *Fmr1*-KO mice treated with indicated treatment, counterstained with eosin hematoxylin. Three regions, a, b, c, are shown with their corresponding position on brain sagittal map. Scale bar 2 mm. Image of brain 8W b is a reuse of Fig 3D Rh10.

B Immunoblots and quantification of ΔN-DGKk protein in Fmr1-WT brain lysates from cortex (c), hippocampus (h), and rest (r) areas as in Fig 3C. GAPDH was used as a loading control. AU, arbitrary units.

C Measure of total phosphatidic acid (PA) level by mass spectrometry in hippocampus and rest of brain of WT mice treated with saline solution (WT) and *Fmr1*-KO mice treated with saline (Fmr1-S), AAVPHP.eB-ΔN-DGKk (Fmr1-PHP.eB), AAVRh10-ΔN-DGKk (Fmr1-Rh10) 8 weeks after injections. Data are expressed as mol % of total lipids and analyzed using one-way ANOVA and Tukey's multiple comparisons test, $n = 8$ individual animals, except for WT and Fmr1-S $n = 11$ and represented as median with interquartile range with minimum and maximum values.

D Total diacylglycerol (DAG) level in cortex measured as in C).

Source data are available online for this figure.

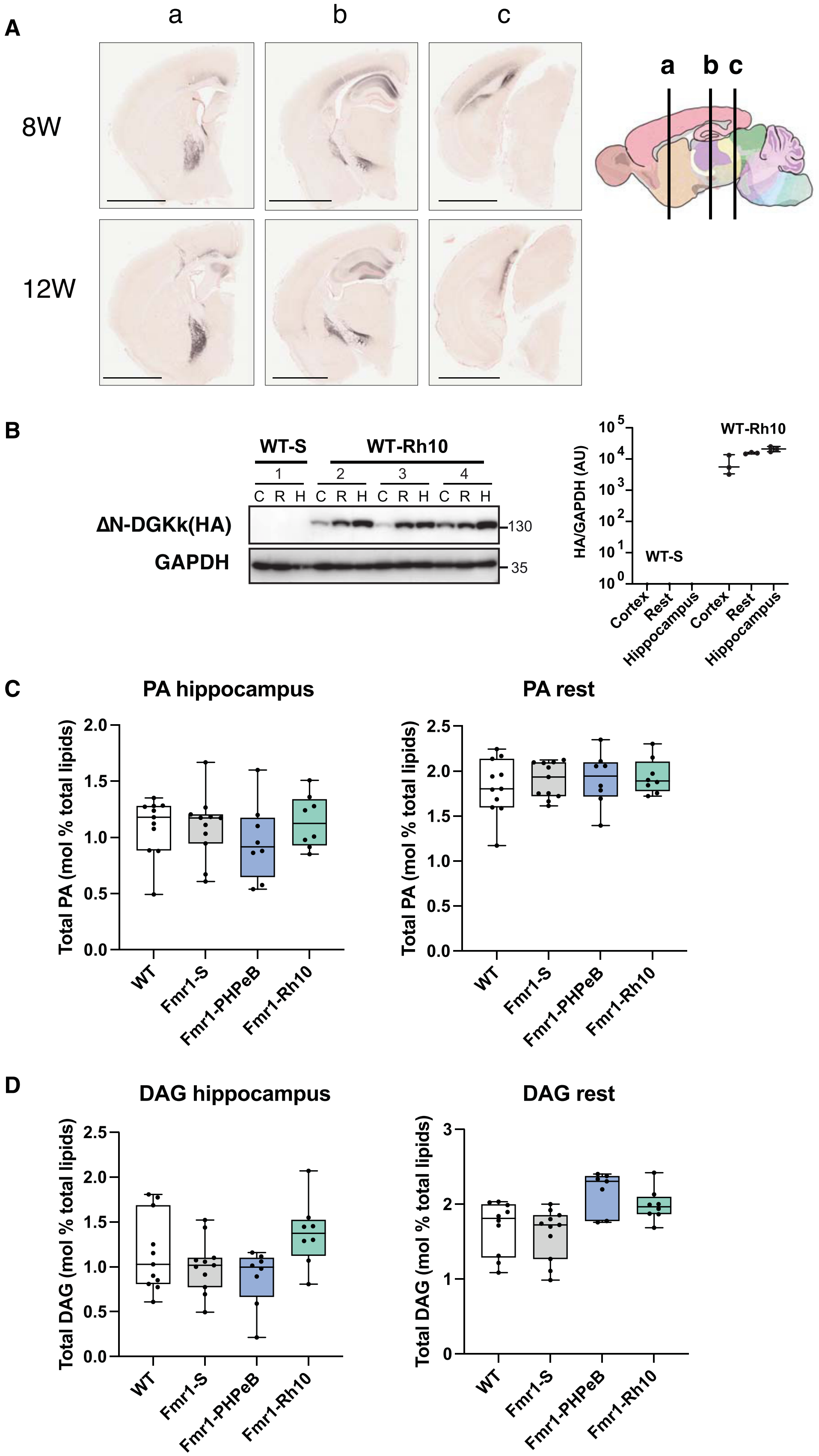


**Figure EV3.**

 © 2022 The Authors

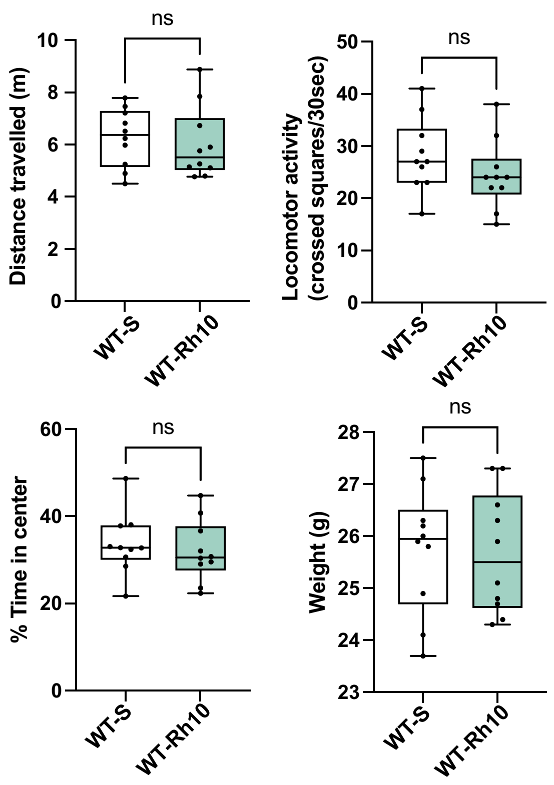


**Figure EV4. AAVRh10-ΔN-DGKk does not lead to visible behavior effect in *Fmr1*-WT mice 4 weeks after its administration.**

Locomotor activity (distance in m and crossed squares per 30 s) in the whole arena and percentage of time spent in the center during 15 min habituation, weight of vehicle-treated WT mice (WT-S) compared to AAVRh10-ΔN-DGKk-treated WT mice (WT-Rh10).

Data information: Data are expressed as median with interquartile range with minimum and maximum values. Statistical analysis: unpaired *t*-test ($n$ = 10 mice per group), ns not significant.
Source data are available online for this figure.

© 2022 The Authors 

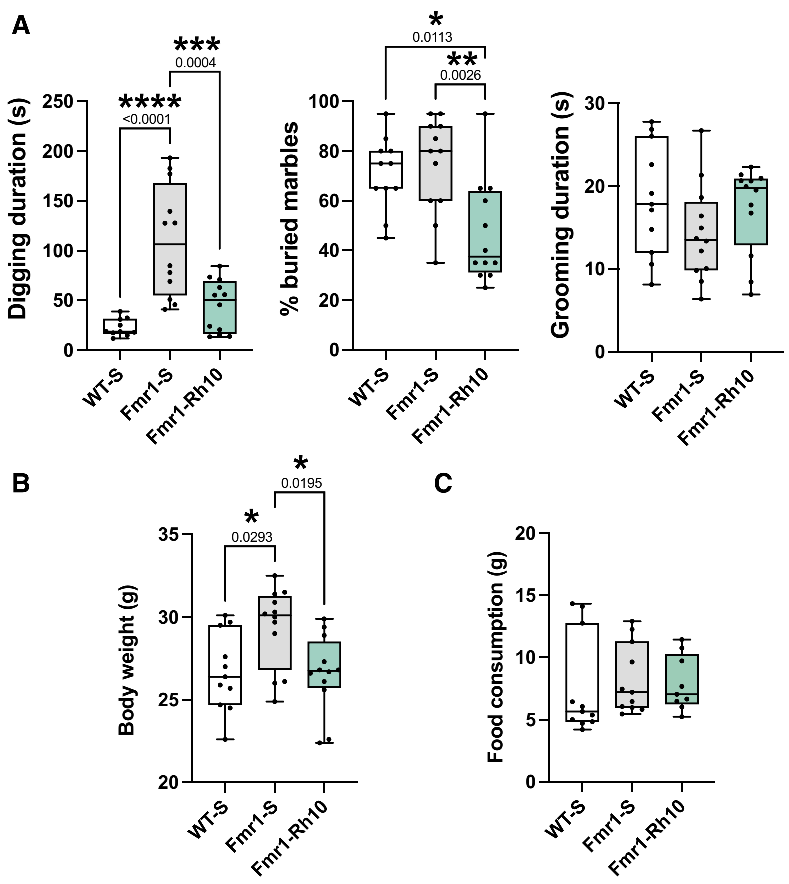


**Figure EV5. Behavioral analyses of AAVRh10-ΔN-DGKk- treated *Fmr1*-KO mice (8 weeks after injection).**

- A Digging, marble burying, and grooming duration tests.
- B Body weight of mice (g).
- C Food consumption (g).

Data information: Data are expressed as median with interquartile range with minimum and maximum values for other panels. Statistical analysis: one-way ANOVA with Tukey's multiple comparisons test ($n$ = 12) *$P$ < 0.05, **$P$ < 0.01, ***$P$ < 0.001, ****$P$ < 0.0001.
Source data are available online for this figure.



© 2022 The Authors
