# Supplementary material for: AAV‐delivered diacylglycerol kinase DGKk achieves long‐term rescue of fragile X syndrome mouse model
Source: EMBO Mol Med. 2022 Apr 4;14(5):e14649. doi: 10.15252/emmm.202114649 (PMC9081908; doi:10.15252/emmm.202114649)
Supplement: Supplementary file 3 — Source Data for Expanded View and Appendix [file EMMM-14-e14649-s002.zip › Source data for EV and Appendix/Source data Fig EV2/Fig EV2B uncropped.pptx]

## Slide 1
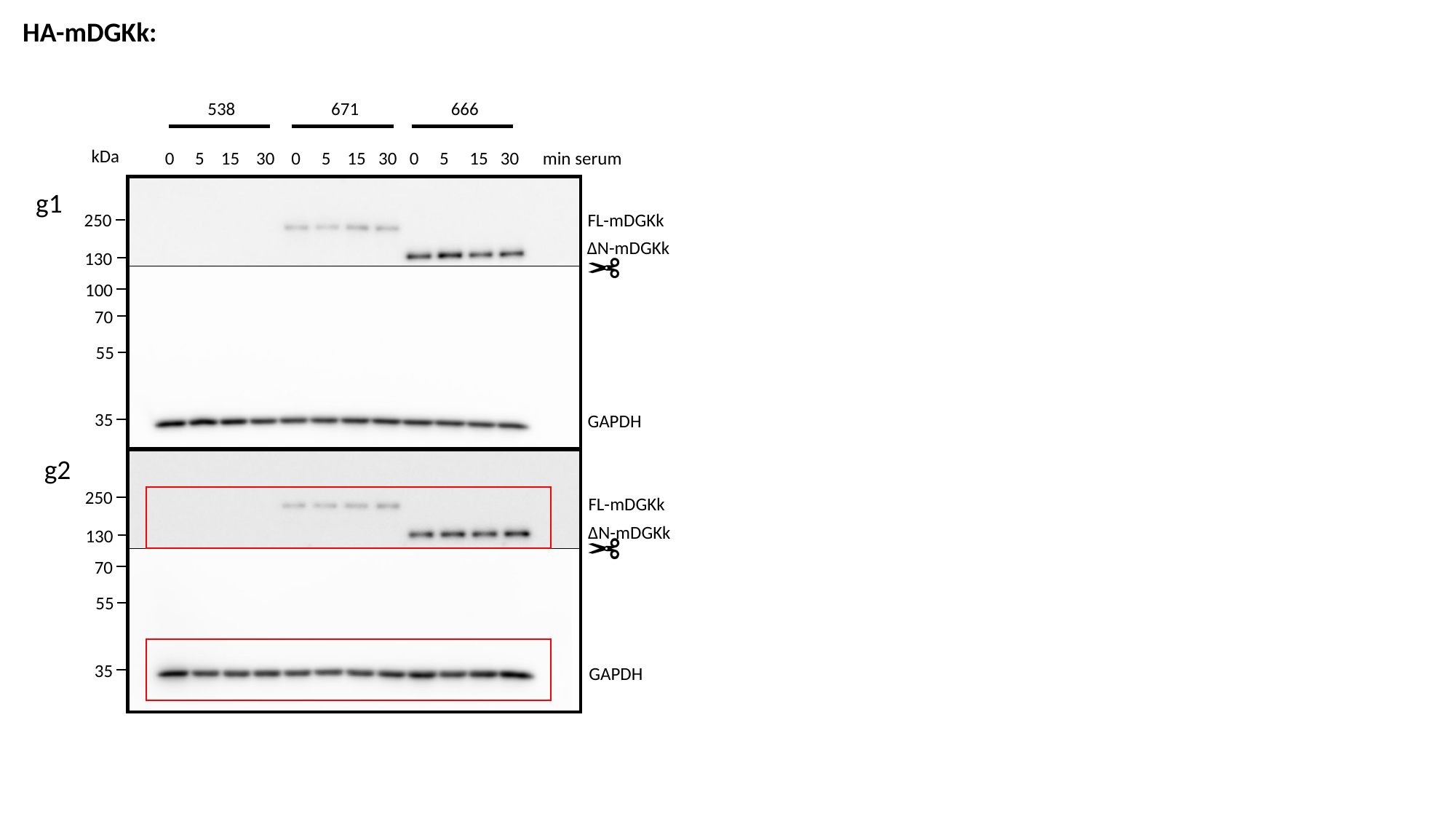

HA-mDGKk:
538
666
671
kDa
min serum
0 5 15 30 0 5 15 30 0 5 15 30
g1
250
FL-mDGKk
ΔN-mDGKk
130
100
70
55
35
GAPDH
g2
250
FL-mDGKk
ΔN-mDGKk
130
70
55
35
GAPDH

## Slide 2
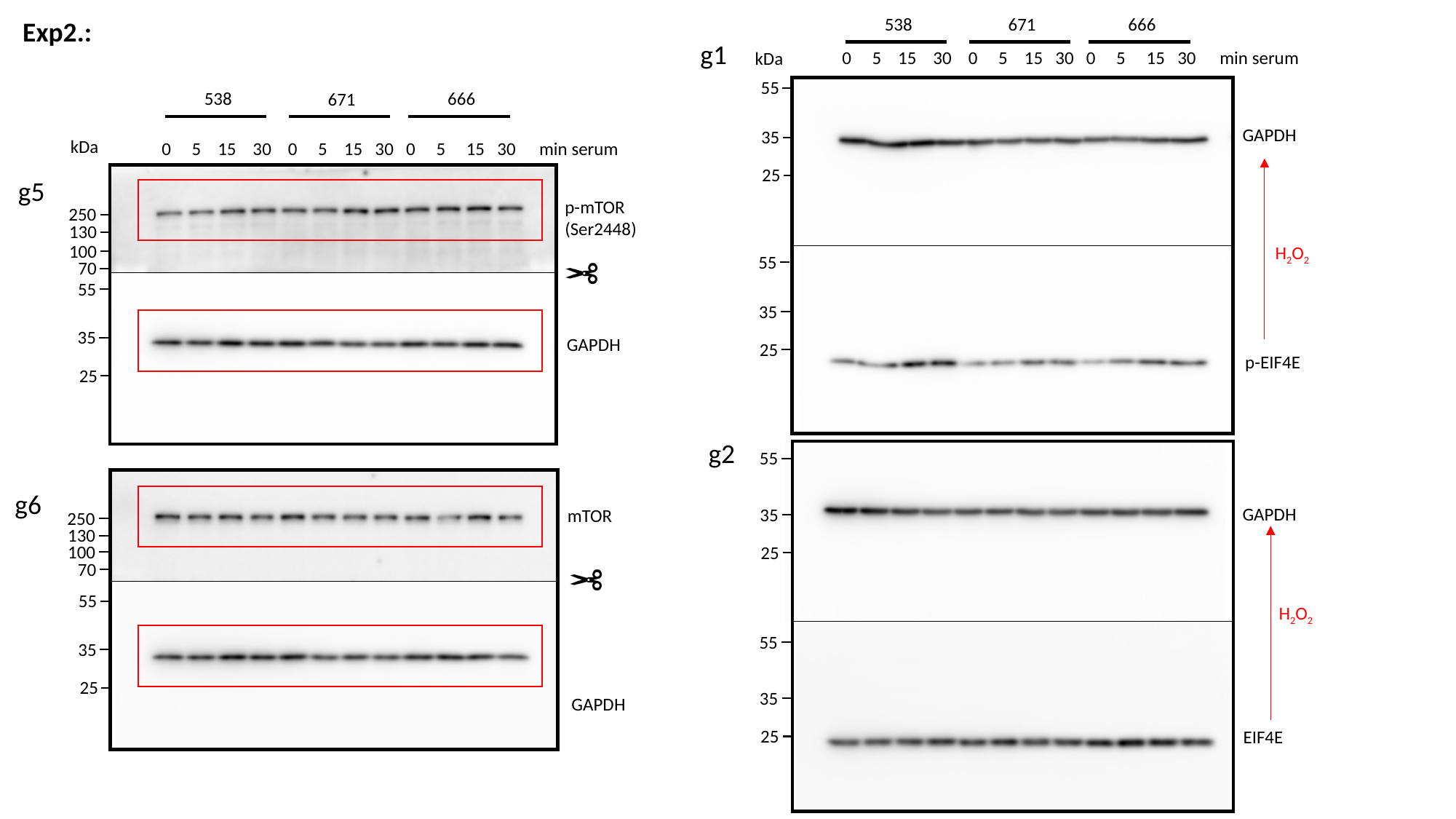

538
666
671
Exp2.:
g1
min serum
0 5 15 30 0 5 15 30 0 5 15 30
kDa
55
538
666
671
GAPDH
35
kDa
min serum
0 5 15 30 0 5 15 30 0 5 15 30
25
g5
p-mTOR (Ser2448)
250
130
100
H2O2
55
70
55
35
35
GAPDH
25
p-EIF4E
25
g2
55
g6
GAPDH
35
mTOR
250
130
100
25
70
55
H2O2
55
35
25
35
GAPDH
25
EIF4E

## Slide 3
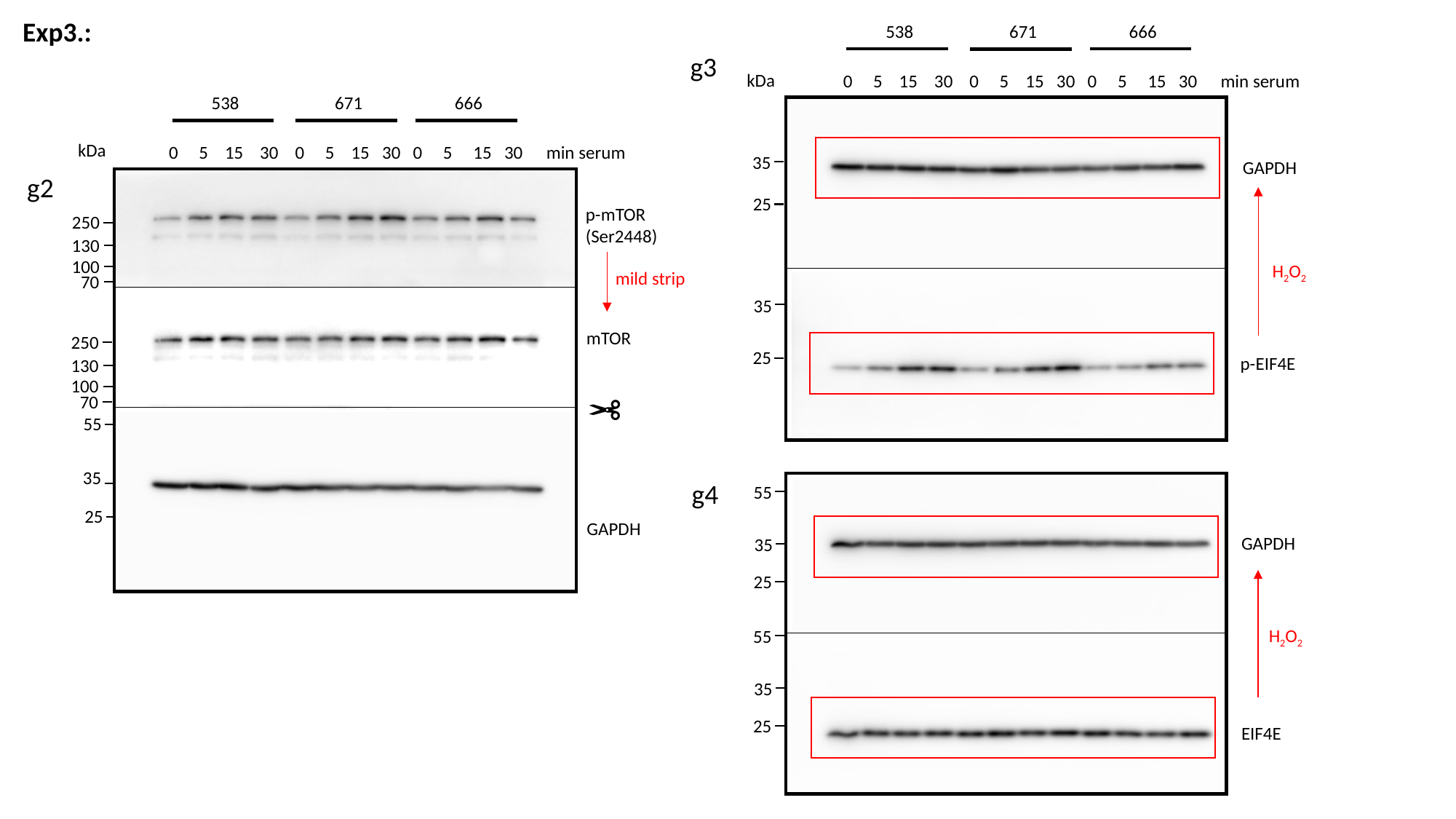

Exp3.:
538
666
671
g3
kDa
min serum
0 5 15 30 0 5 15 30 0 5 15 30
538
666
671
kDa
min serum
0 5 15 30 0 5 15 30 0 5 15 30
35
GAPDH
g2
25
p-mTOR (Ser2448)
250
130
100
H2O2
mild strip
70
35
mTOR
250
25
p-EIF4E
130
100
70
55
35
g4
55
25
GAPDH
GAPDH
35
25
H2O2
55
35
25
EIF4E

## Slide 4
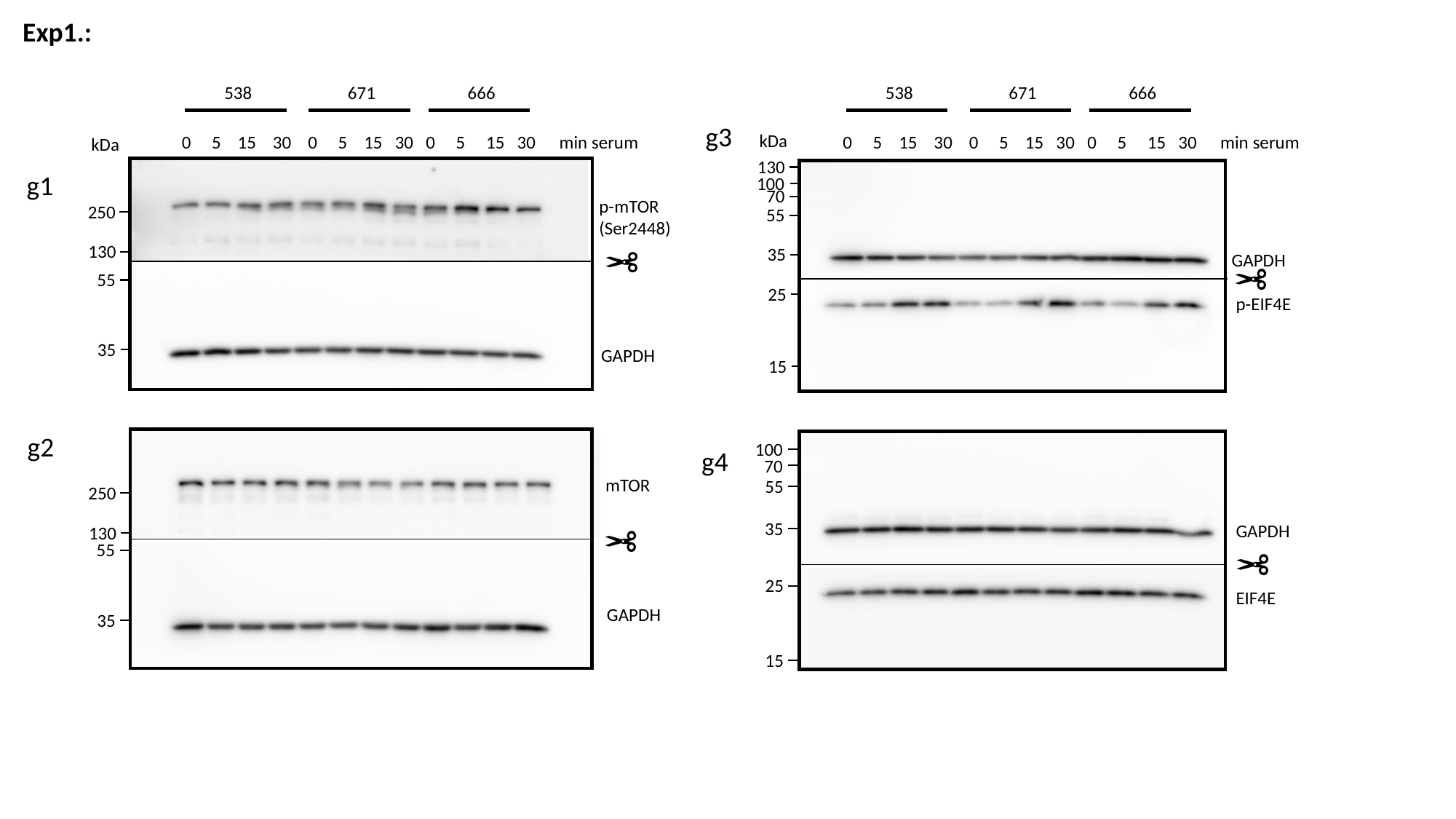

Exp1.:
538
666
671
538
666
671
g3
kDa
min serum
0 5 15 30 0 5 15 30 0 5 15 30
min serum
0 5 15 30 0 5 15 30 0 5 15 30
kDa
130
g1
100
70
p-mTOR (Ser2448)
250
55
130
35
GAPDH
55
25
p-EIF4E
35
GAPDH
15
g2
100
g4
70
mTOR
55
250
35
GAPDH
130
55
25
EIF4E
GAPDH
35
15
