# Supplementary figures and images for: AAV‐delivered diacylglycerol kinase DGKk achieves long‐term rescue of fragile X syndrome mouse model
Source: EMBO Mol Med. 2022 Apr 4;14(5):e14649. doi: 10.15252/emmm.202114649 (PMC9081908; doi:10.15252/emmm.202114649)

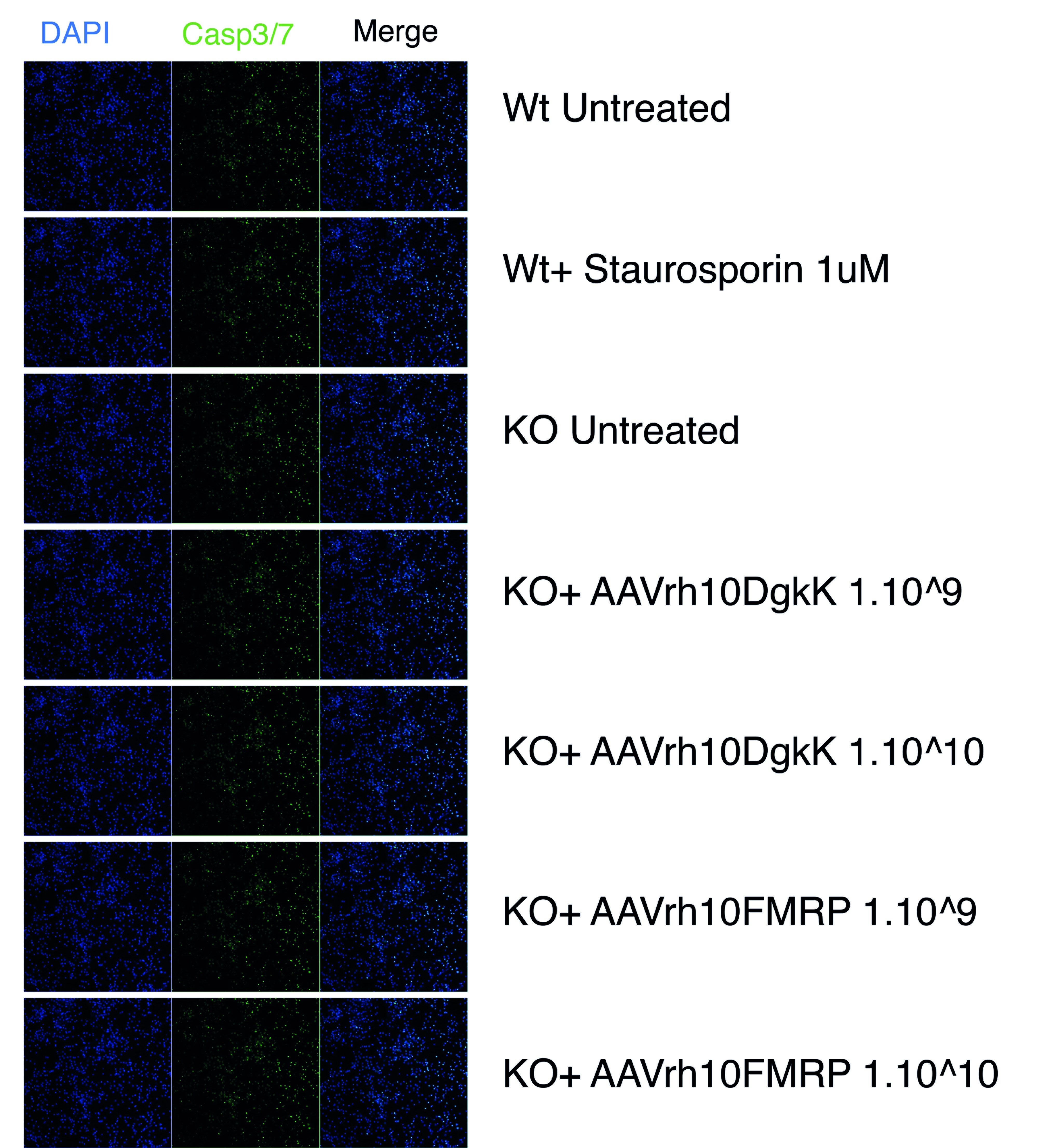

Supplement: Supplementary file 3 — Source Data for Expanded View and Appendix [file EMMM-14-e14649-s002.zip › Source data for EV and Appendix/Source data Fig EV2/Source data Fig EV2 CEF_Casp3-7.jpg]

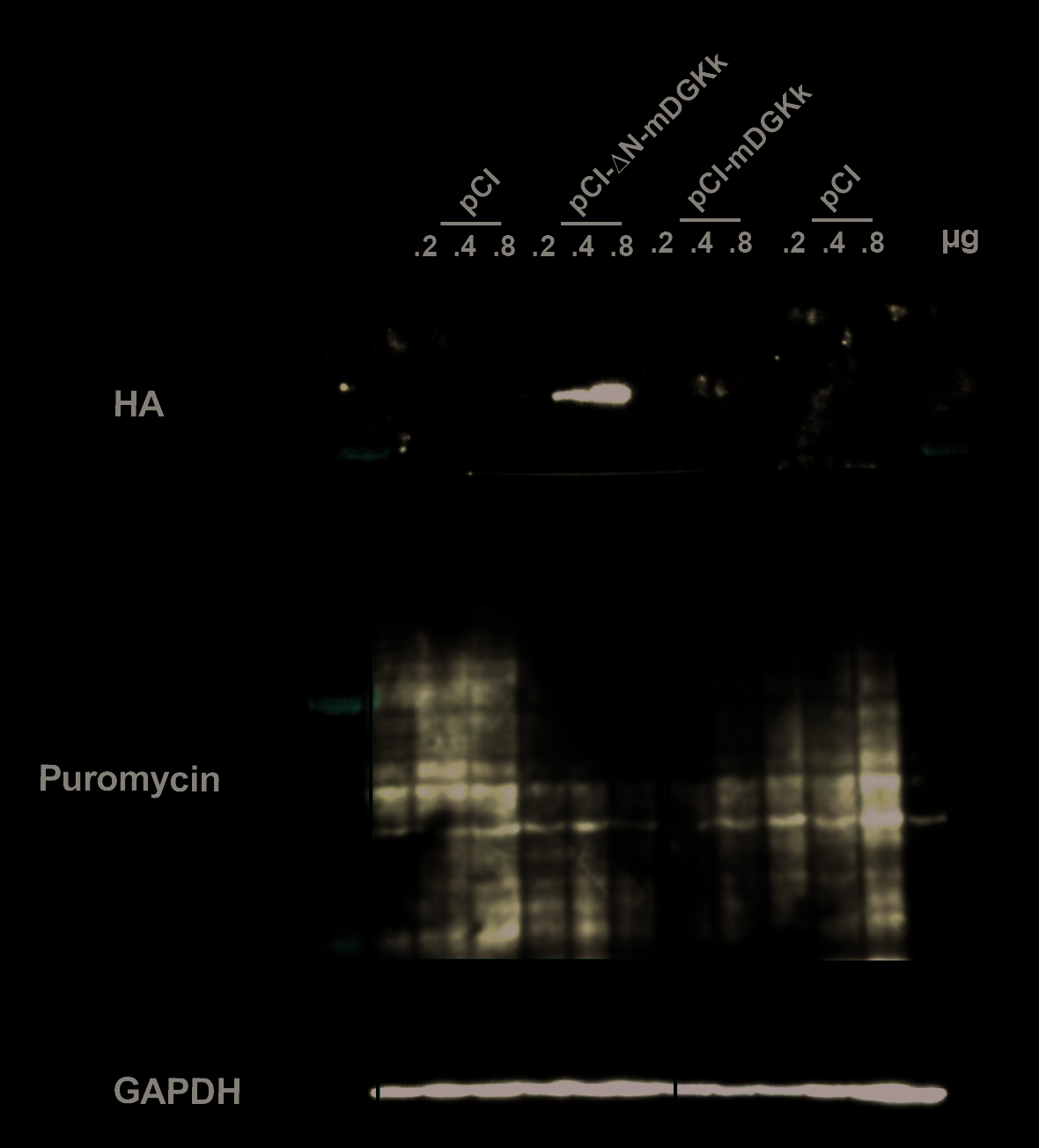

Supplement: Supplementary file 3 — Source Data for Expanded View and Appendix [file EMMM-14-e14649-s002.zip › Source data for EV and Appendix/Source data Fig EV2/Fig EV2A dosage uncropped.tif]

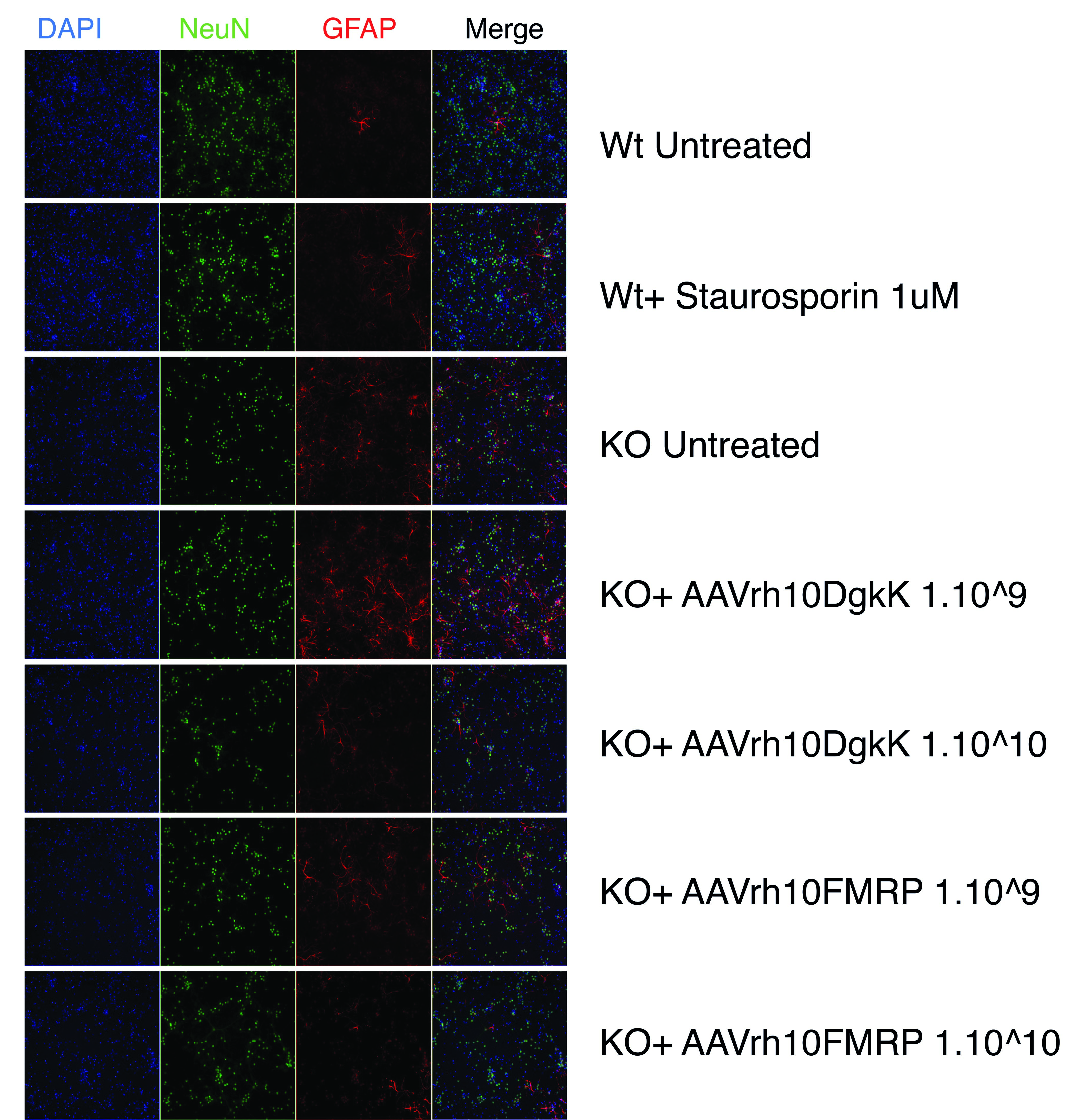

Supplement: Supplementary file 3 — Source Data for Expanded View and Appendix [file EMMM-14-e14649-s002.zip › Source data for EV and Appendix/Source data Fig EV2/Source data Fig EV2 CEF_Neun-GFAP.jpg]

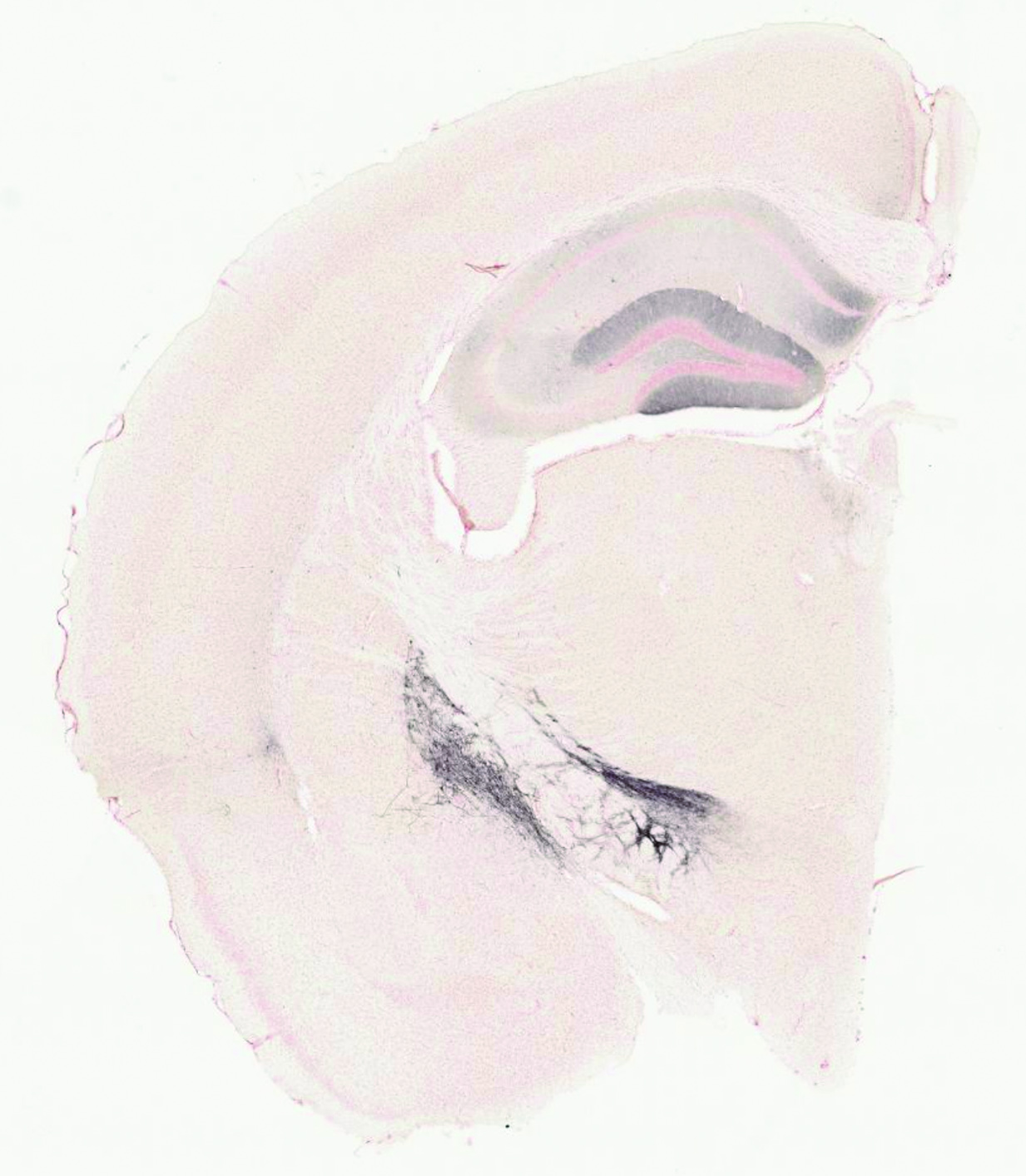

Supplement: Supplementary file 3 — Source Data for Expanded View and Appendix [file EMMM-14-e14649-s002.zip › Source data for EV and Appendix/Source data Fig EV3/Source data Fig EV3A/KO 266 Rh10 12w - hip_15.jpg]

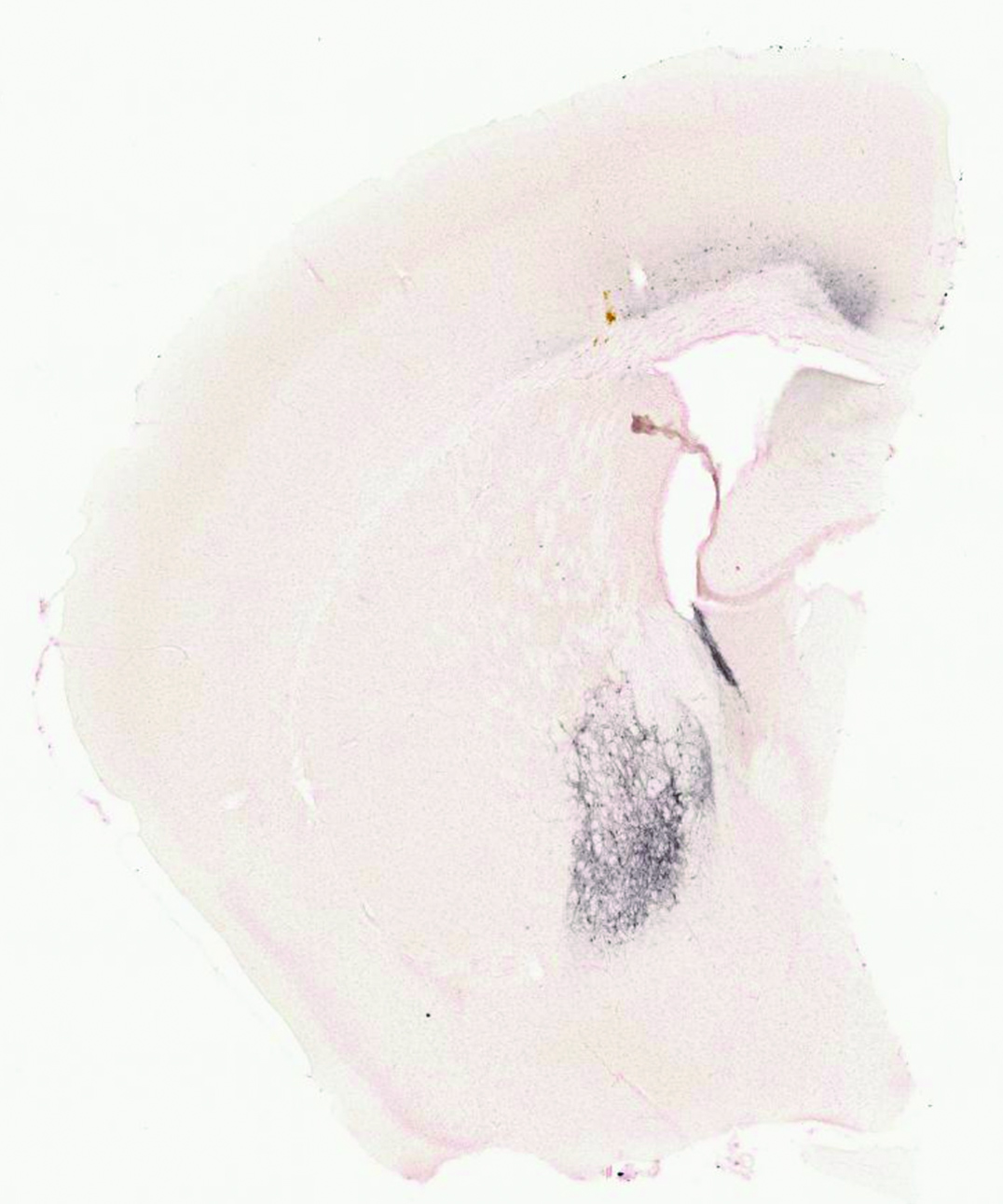

Supplement: Supplementary file 3 — Source Data for Expanded View and Appendix [file EMMM-14-e14649-s002.zip › Source data for EV and Appendix/Source data Fig EV3/Source data Fig EV3A/KO 7 Rh10 8w - str_12.jpg]

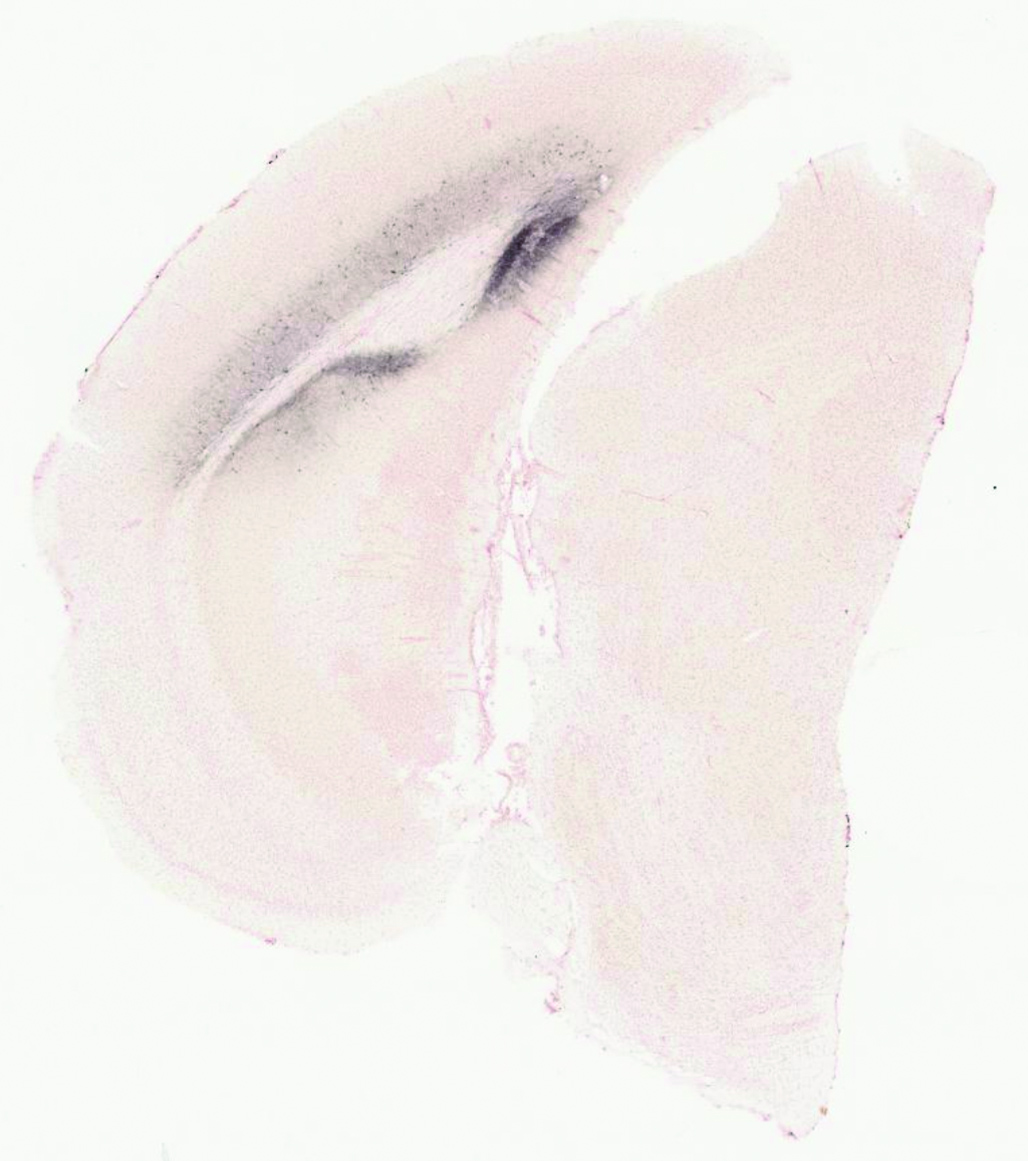

Supplement: Supplementary file 3 — Source Data for Expanded View and Appendix [file EMMM-14-e14649-s002.zip › Source data for EV and Appendix/Source data Fig EV3/Source data Fig EV3A/KO 7 Rh10 8w - pfc_21.jpg]

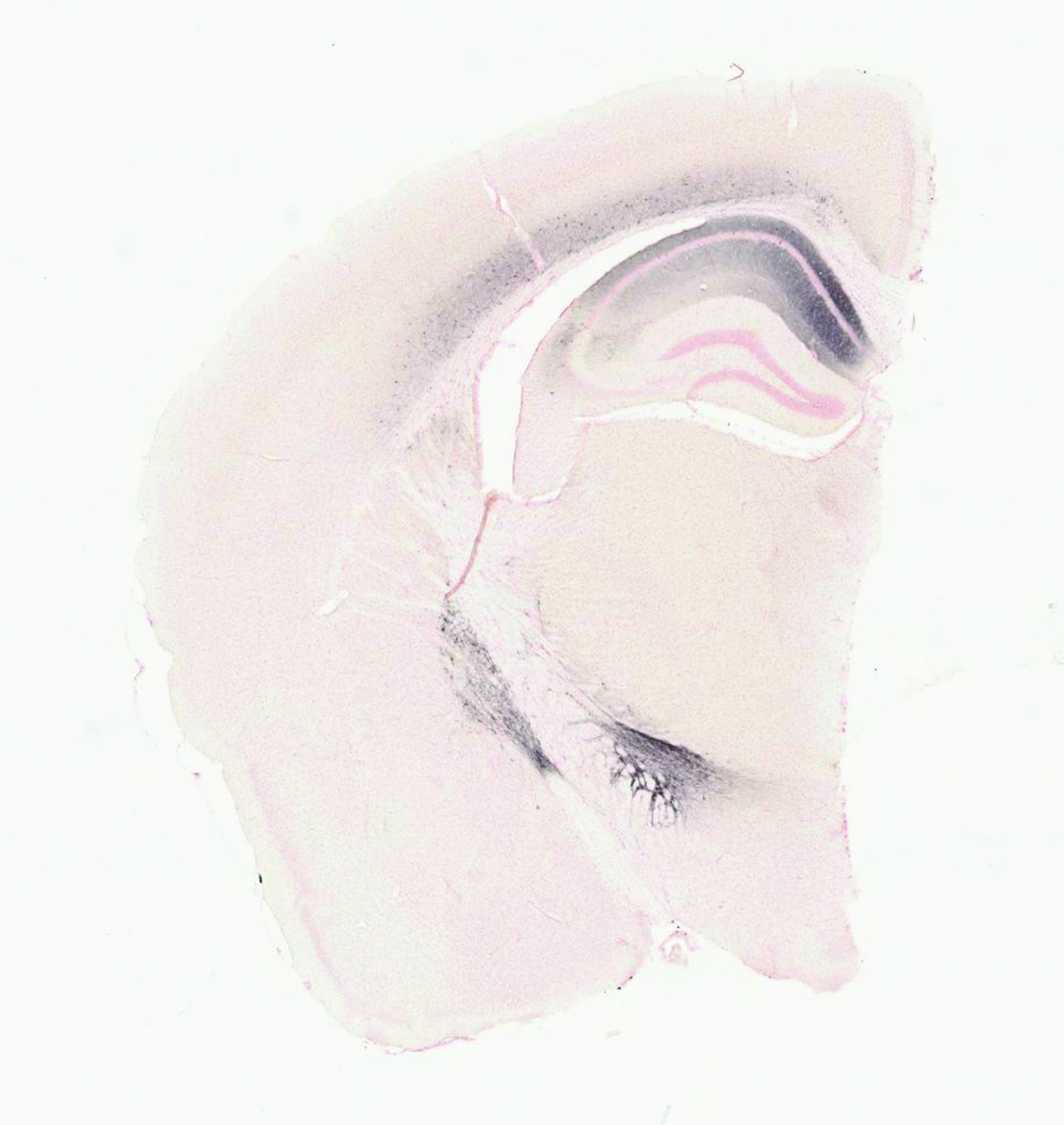

Supplement: Supplementary file 3 — Source Data for Expanded View and Appendix [file EMMM-14-e14649-s002.zip › Source data for EV and Appendix/Source data Fig EV3/Source data Fig EV3A/KO 7 Rh10 8w - hip_15.jpg]

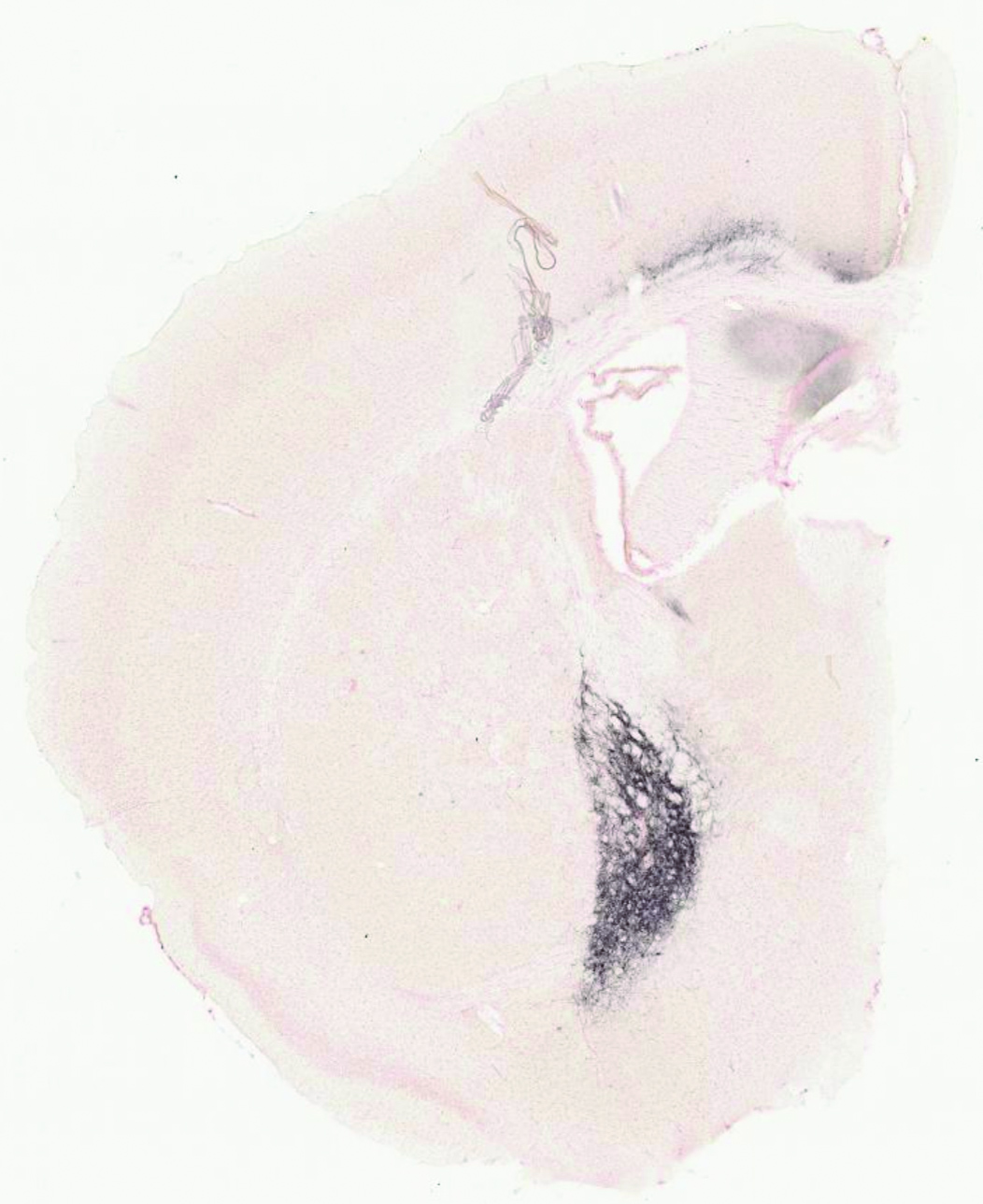

Supplement: Supplementary file 3 — Source Data for Expanded View and Appendix [file EMMM-14-e14649-s002.zip › Source data for EV and Appendix/Source data Fig EV3/Source data Fig EV3A/KO 266 Rh10 12w - str_12.jpg]

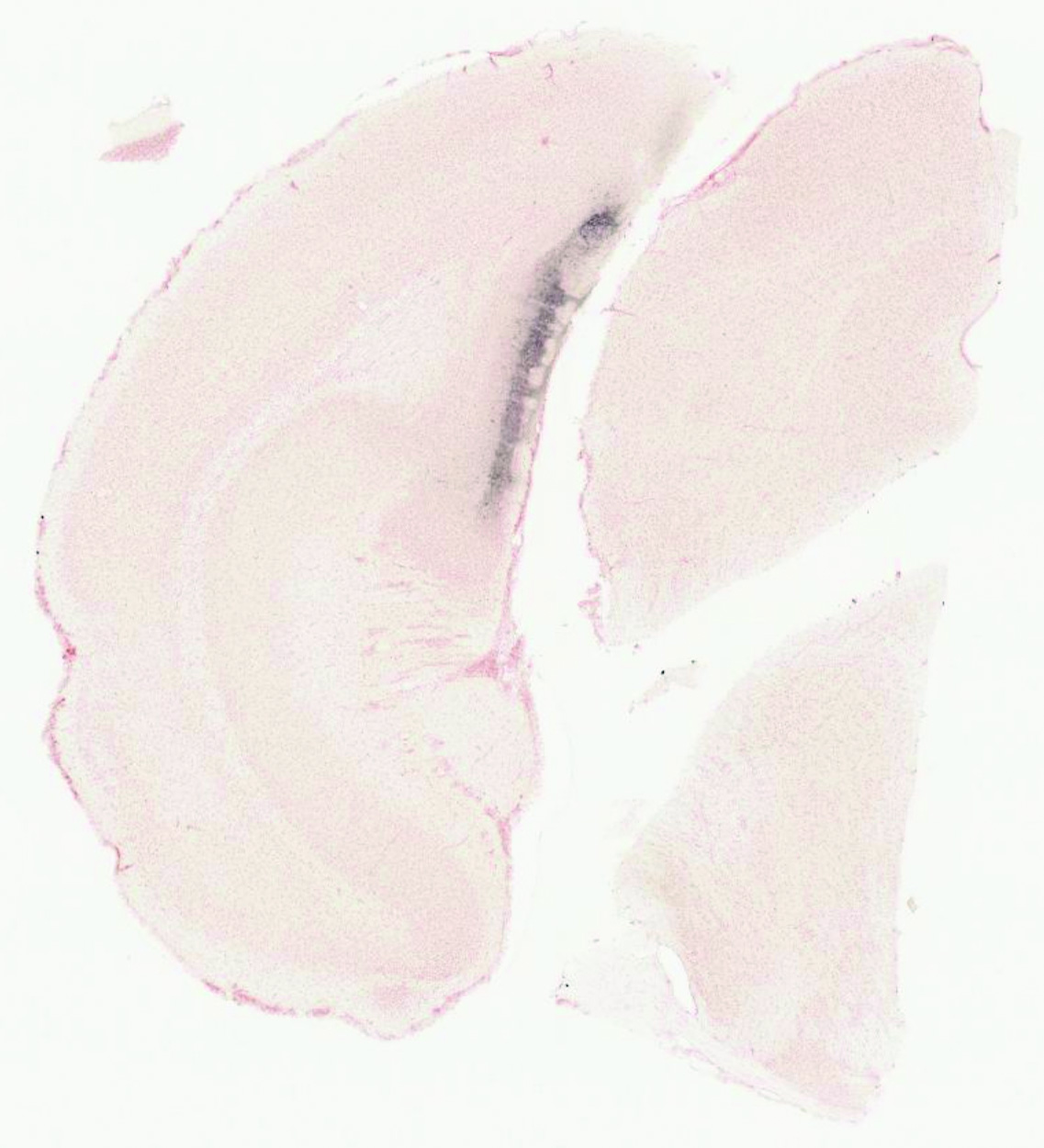

Supplement: Supplementary file 3 — Source Data for Expanded View and Appendix [file EMMM-14-e14649-s002.zip › Source data for EV and Appendix/Source data Fig EV3/Source data Fig EV3A/KO 266 Rh10 12w - pfc_21.jpg]

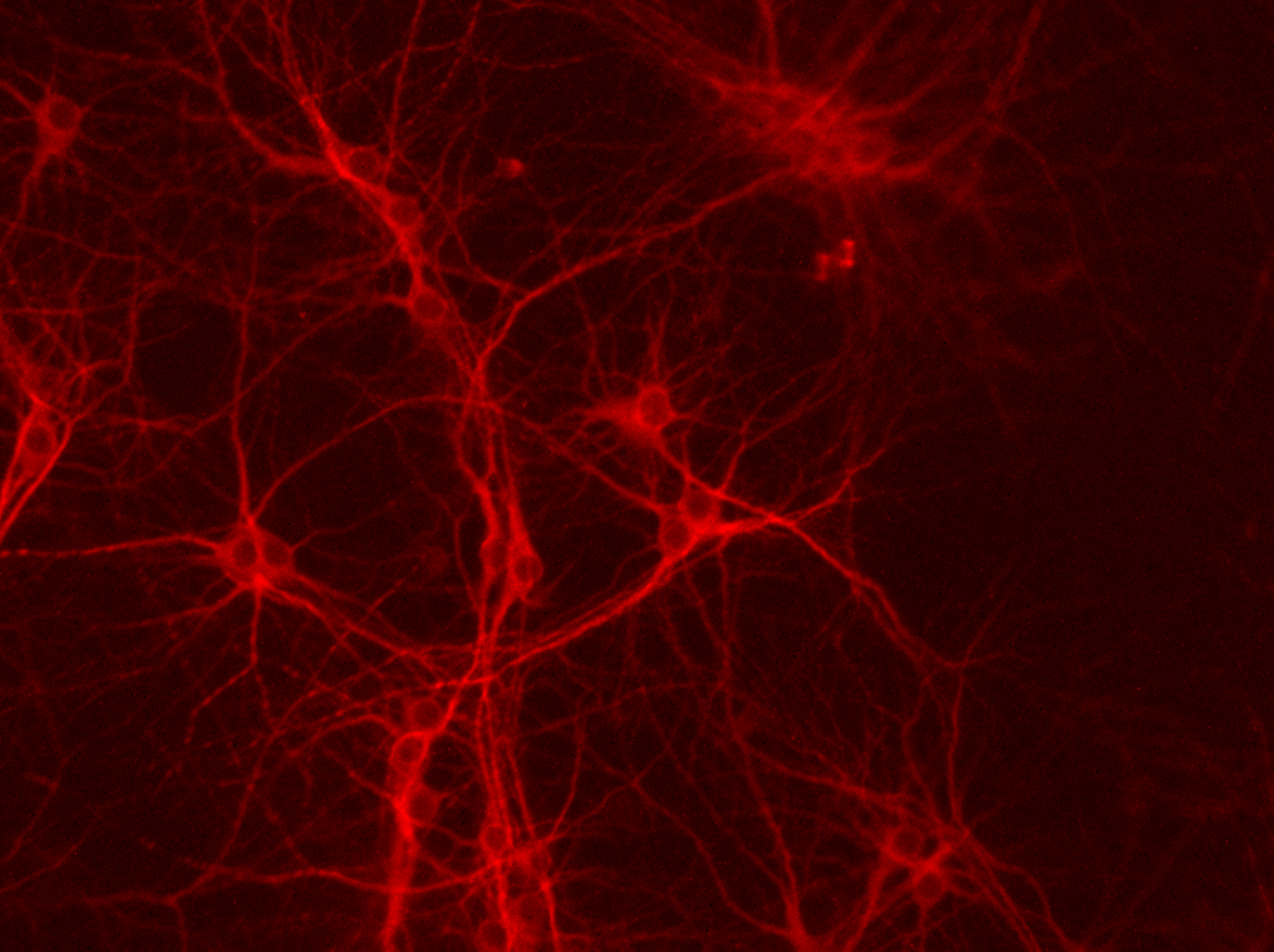

Supplement: Supplementary file 5 — Source Data for Figure 2 [file EMMM-14-e14649-s004.zip › Source_data_Fig2/Source_data_Fig2C/1_E19_KO_AAVRh10GFP-antiMAP2_Red.tif]

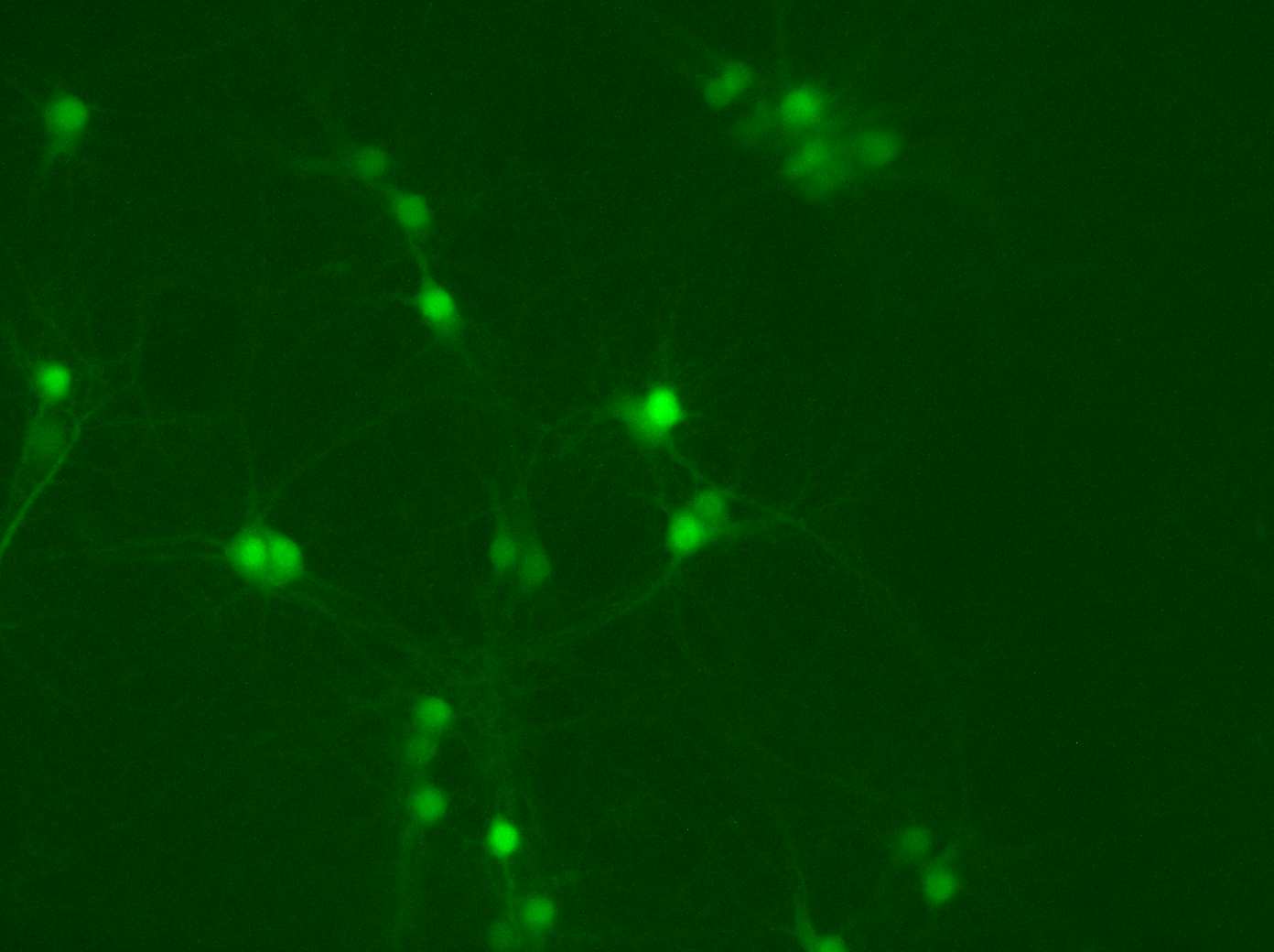

Supplement: Supplementary file 5 — Source Data for Figure 2 [file EMMM-14-e14649-s004.zip › Source_data_Fig2/Source_data_Fig2C/1_E19_KO_AAVRh10GFP_Green.tif]

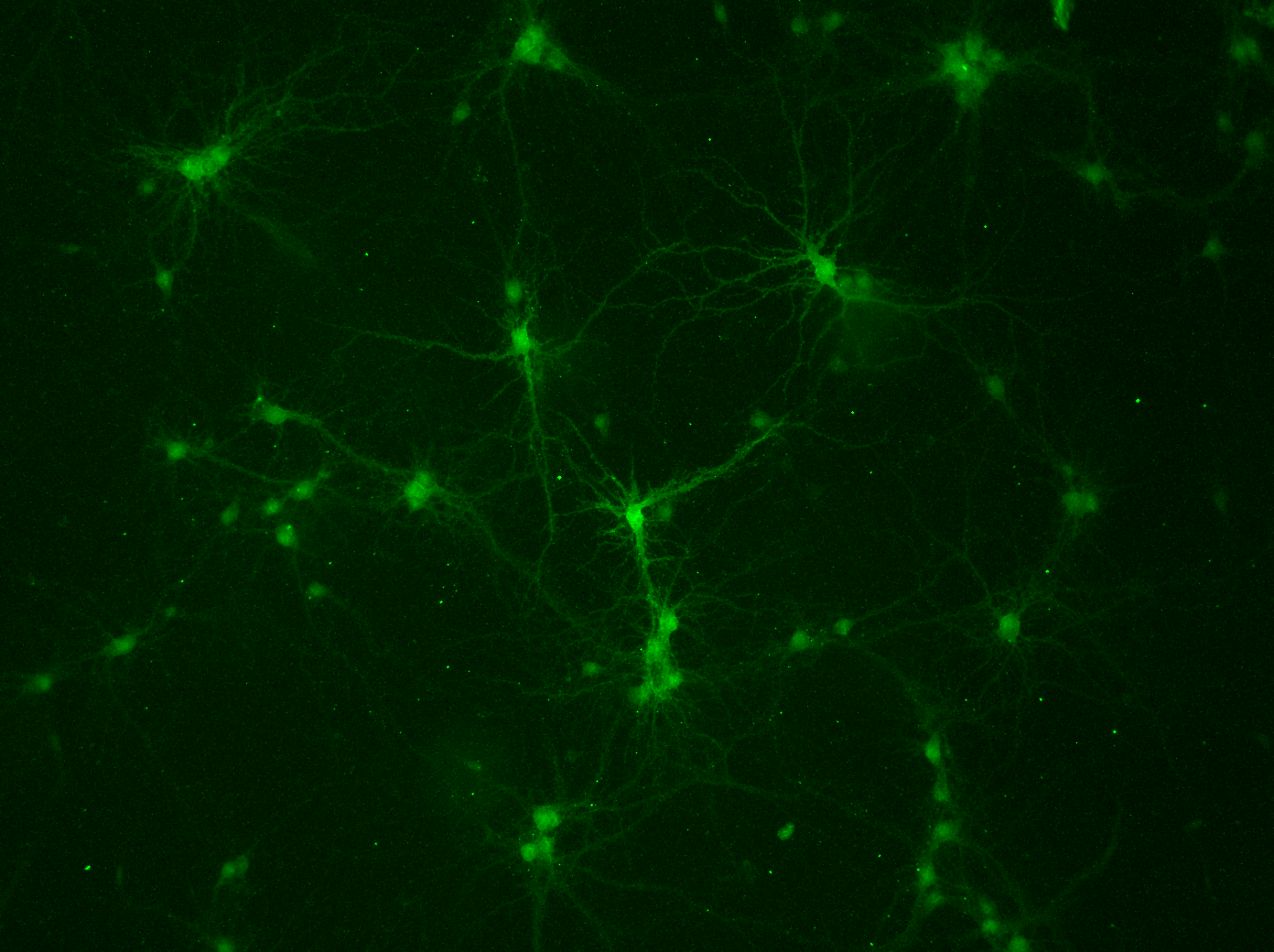

Supplement: Supplementary file 5 — Source Data for Figure 2 [file EMMM-14-e14649-s004.zip › Source_data_Fig2/Source_data_Fig2C/2_E19_KO_AAVrh10ΓêåNDgkk-antiHA_Green.tif]

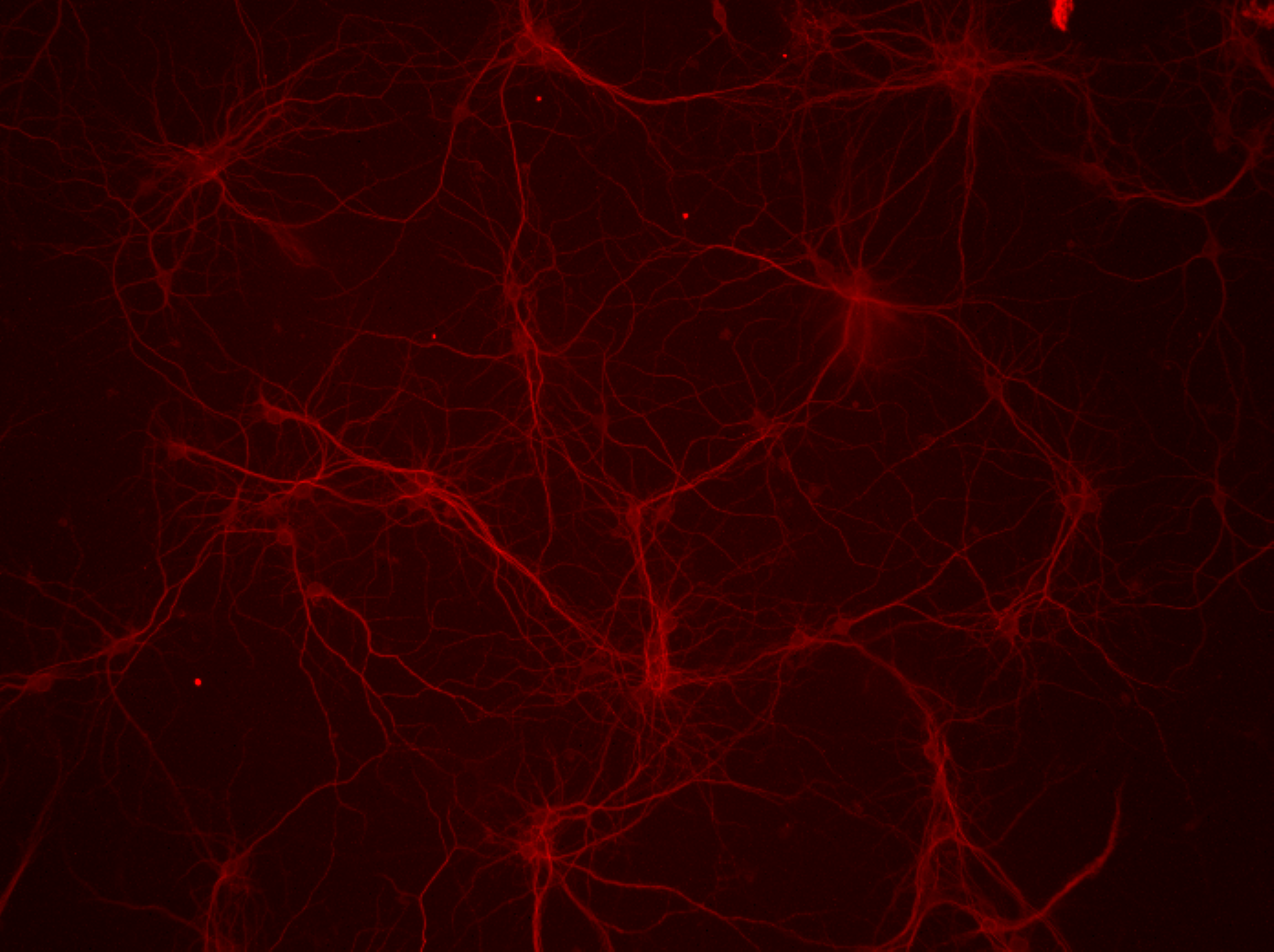

Supplement: Supplementary file 5 — Source Data for Figure 2 [file EMMM-14-e14649-s004.zip › Source_data_Fig2/Source_data_Fig2C/2_E19_KO_AAVRh10ΓêåNDgkk-antiMAP2_Red.tif]

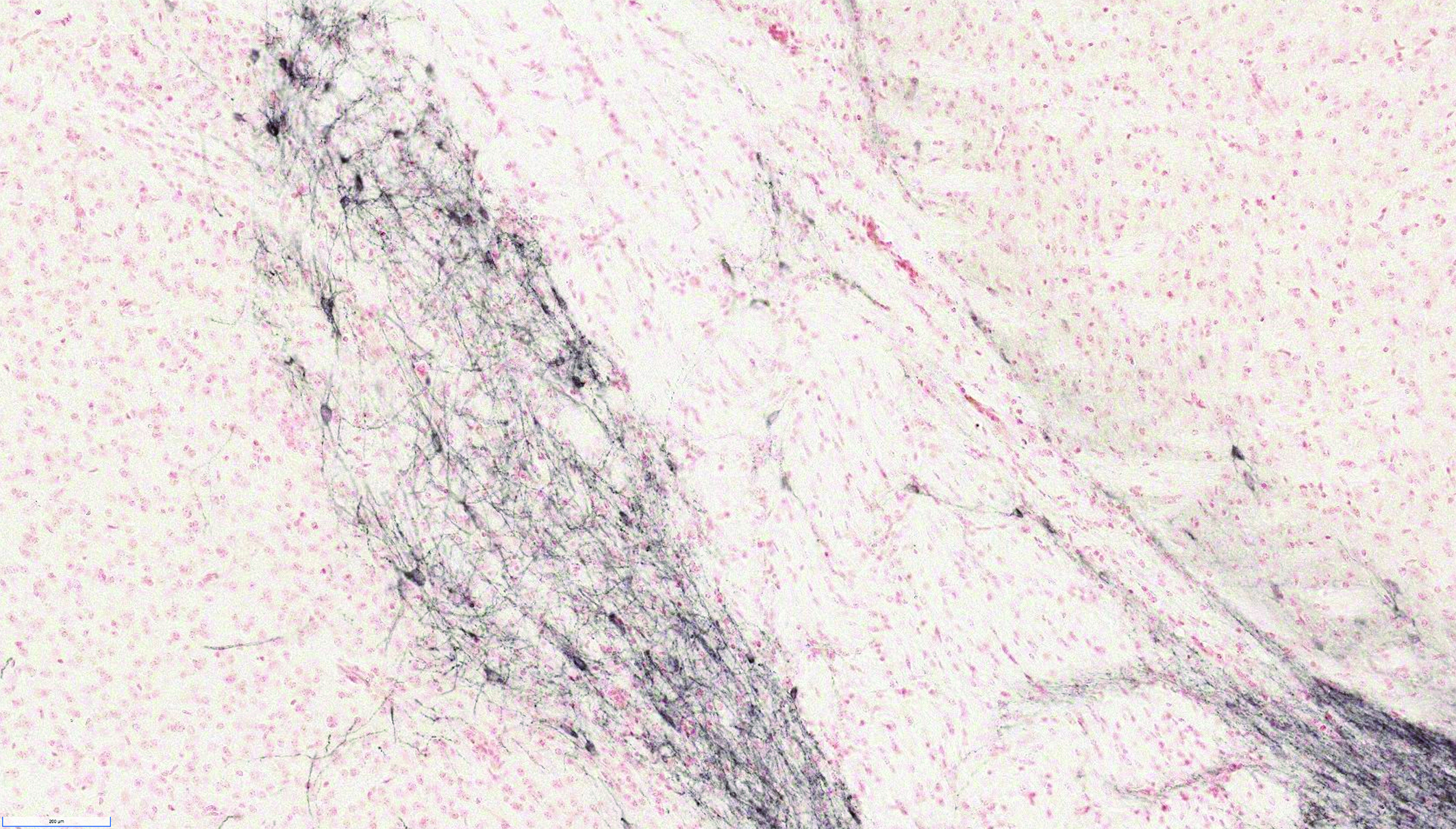

Supplement: Supplementary file 6 — Source Data for Figure 3 [file EMMM-14-e14649-s001.zip › Source data Fig3/Source data Fig3D/Zoom_str-KO 7 PHPeB 4w - hip_15.jpg]

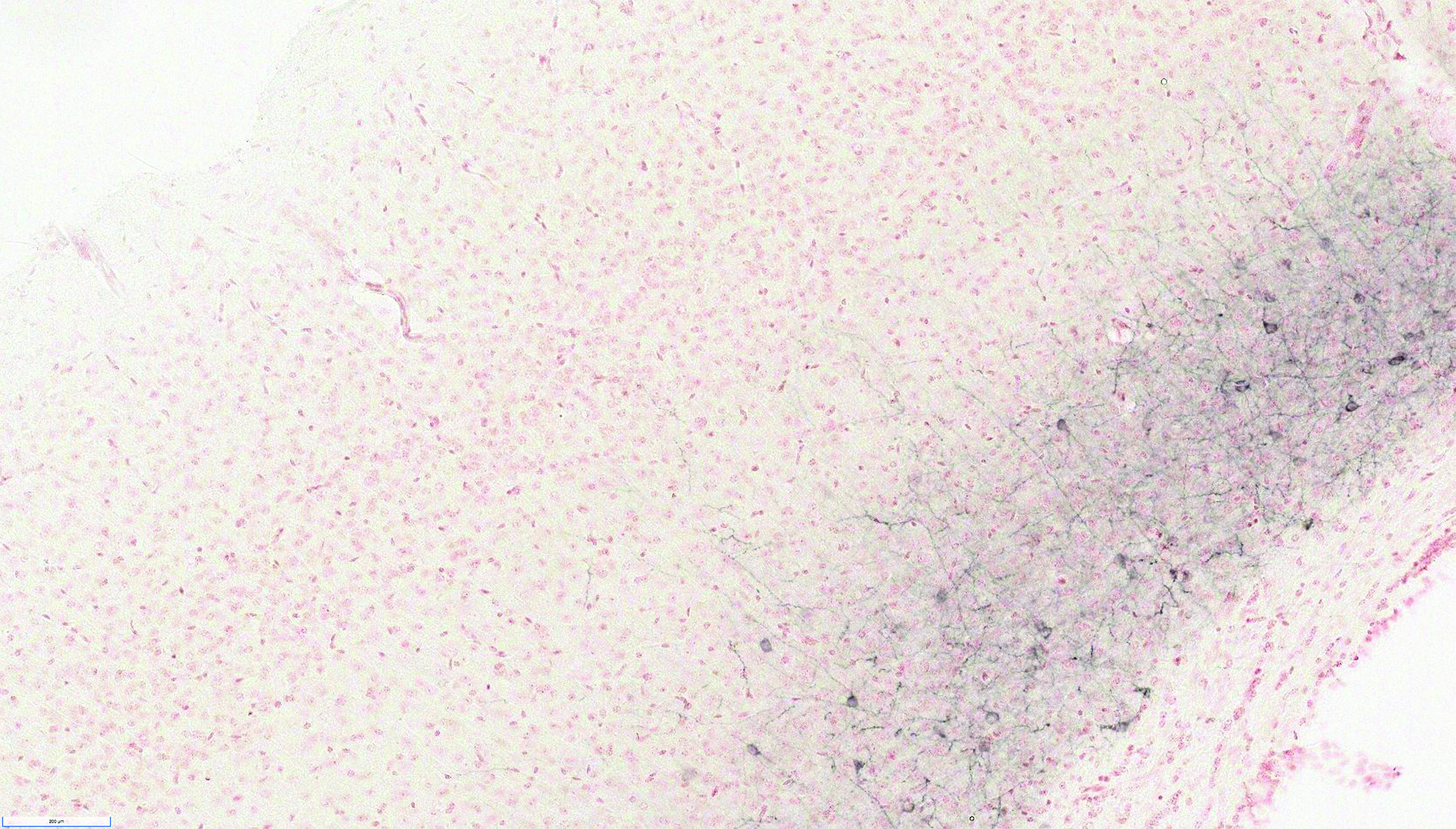

Supplement: Supplementary file 6 — Source Data for Figure 3 [file EMMM-14-e14649-s001.zip › Source data Fig3/Source data Fig3D/Zoom_cor-KO 7 PHPeB 4w - hip_15.jpg]

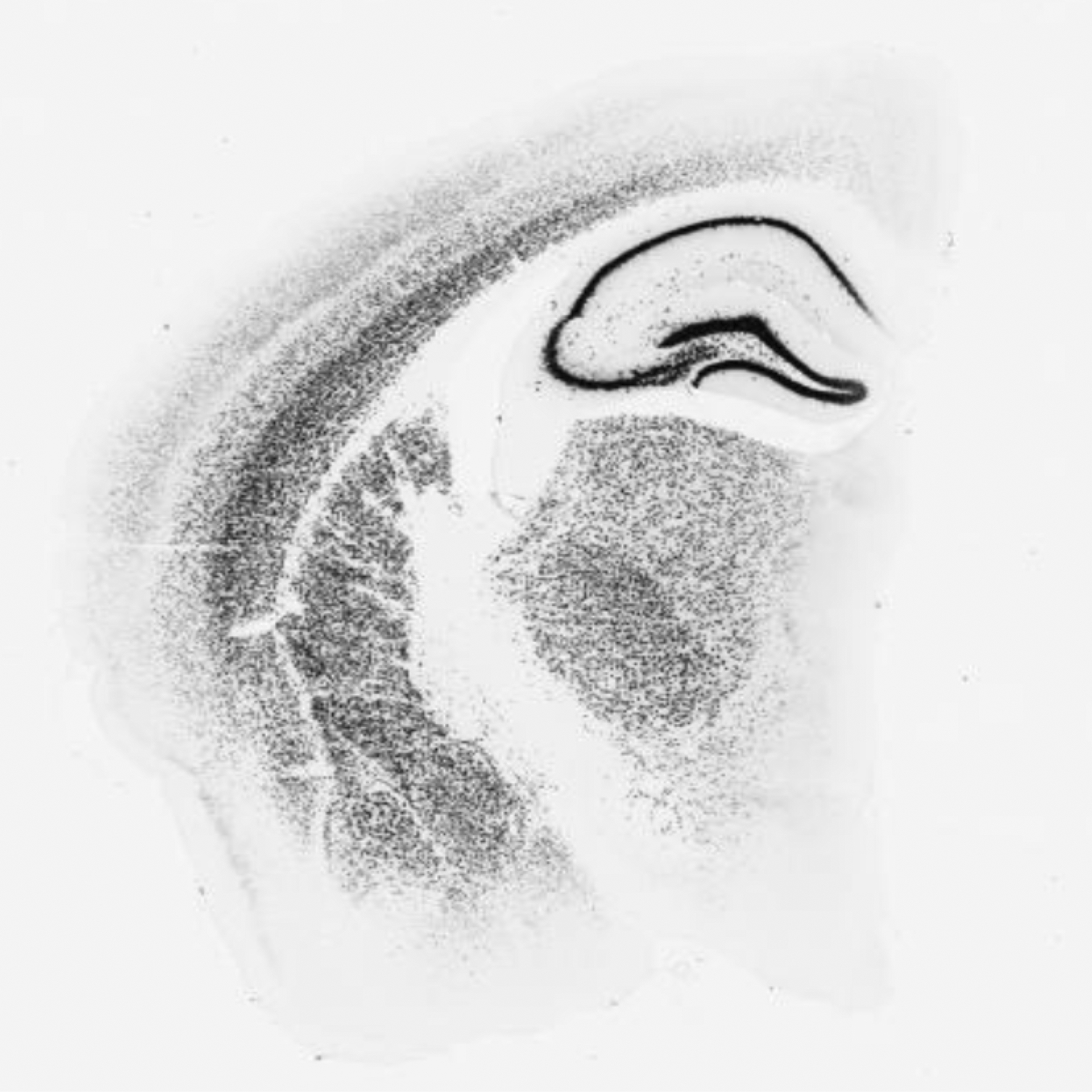

Supplement: Supplementary file 6 — Source Data for Figure 3 [file EMMM-14-e14649-s001.zip › Source data Fig3/Source data Fig3D/KO 200 PHPeB 4w - hip_NeuN_15.jpg]

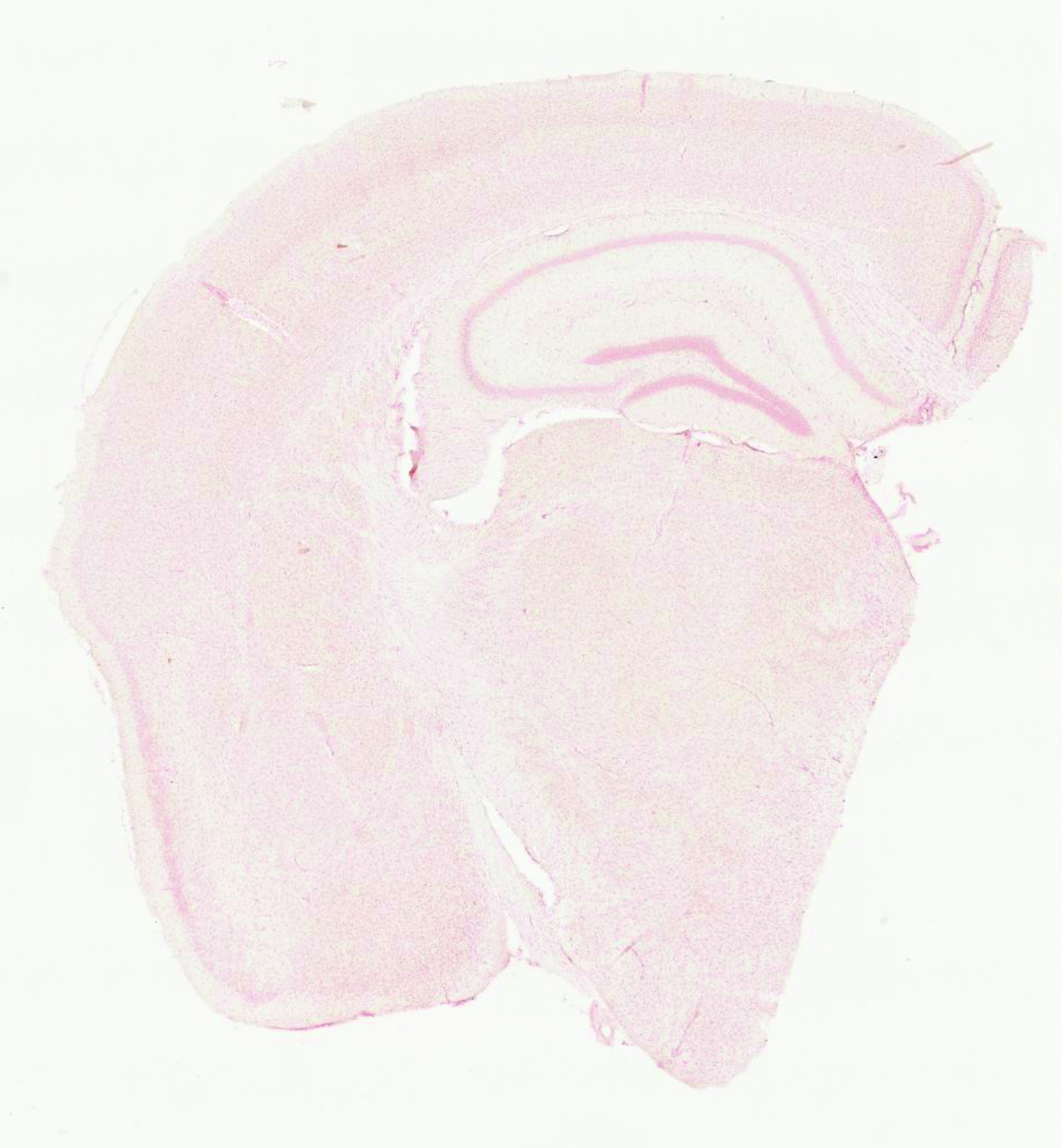

Supplement: Supplementary file 6 — Source Data for Figure 3 [file EMMM-14-e14649-s001.zip › Source data Fig3/Source data Fig3D/KO 200 PHPeB 4w - hip_HA_15.jpg]

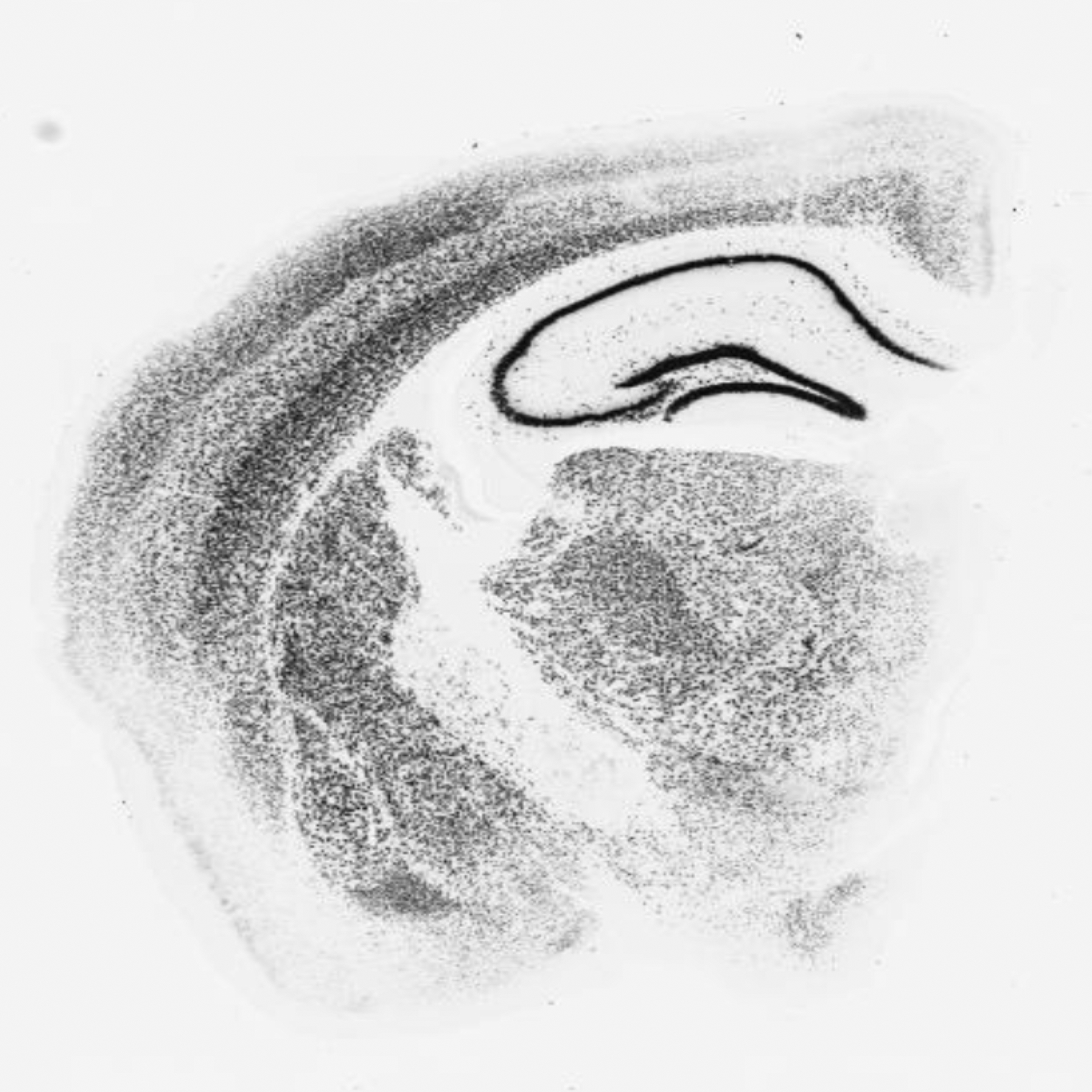

Supplement: Supplementary file 6 — Source Data for Figure 3 [file EMMM-14-e14649-s001.zip › Source data Fig3/Source data Fig3D/KO 412 vehicle 4w - hip_NeuN_15.jpg]

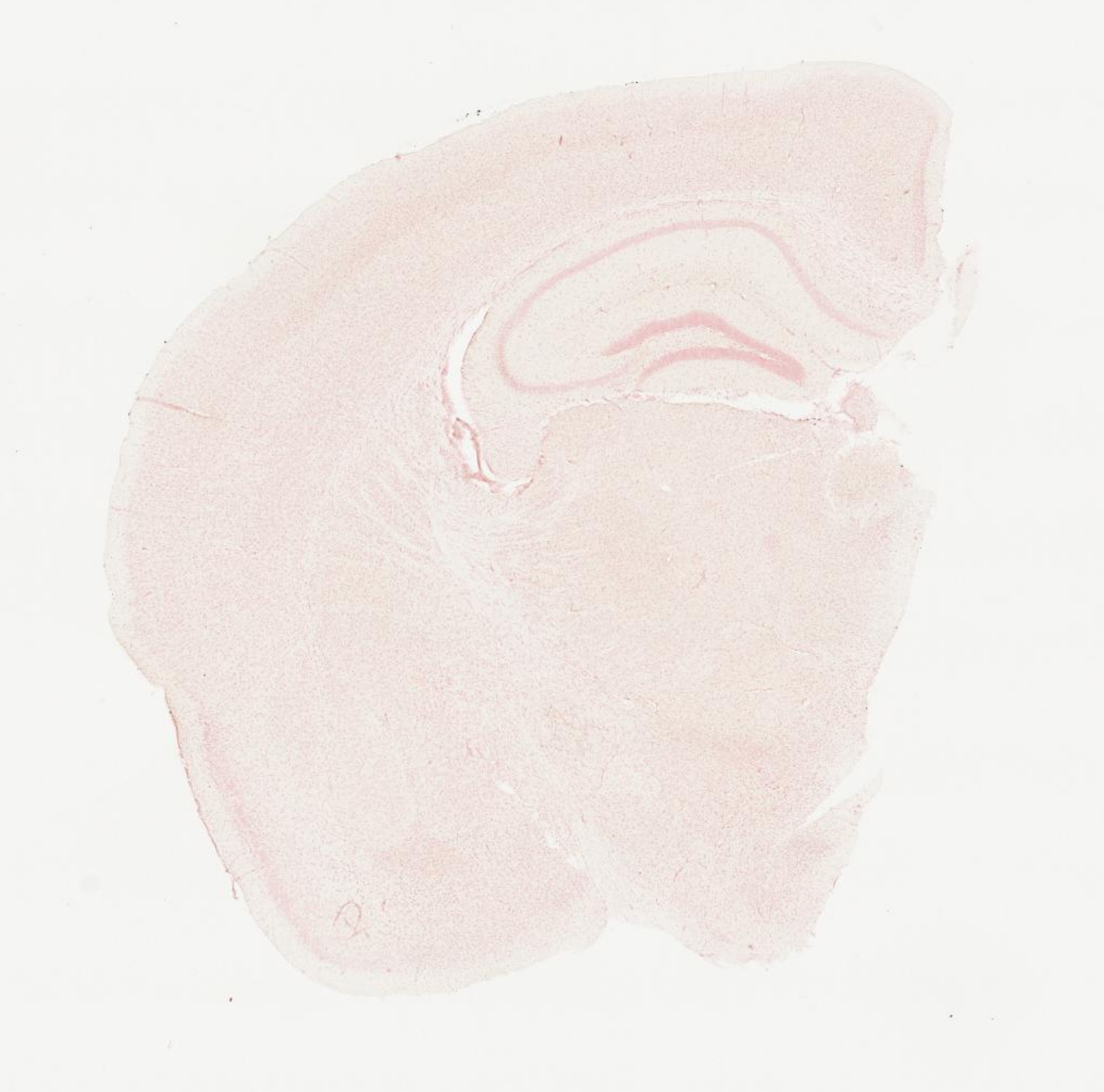

Supplement: Supplementary file 6 — Source Data for Figure 3 [file EMMM-14-e14649-s001.zip › Source data Fig3/Source data Fig3D/KO 412 vehicle 4w - hip_HA_15.jpg]

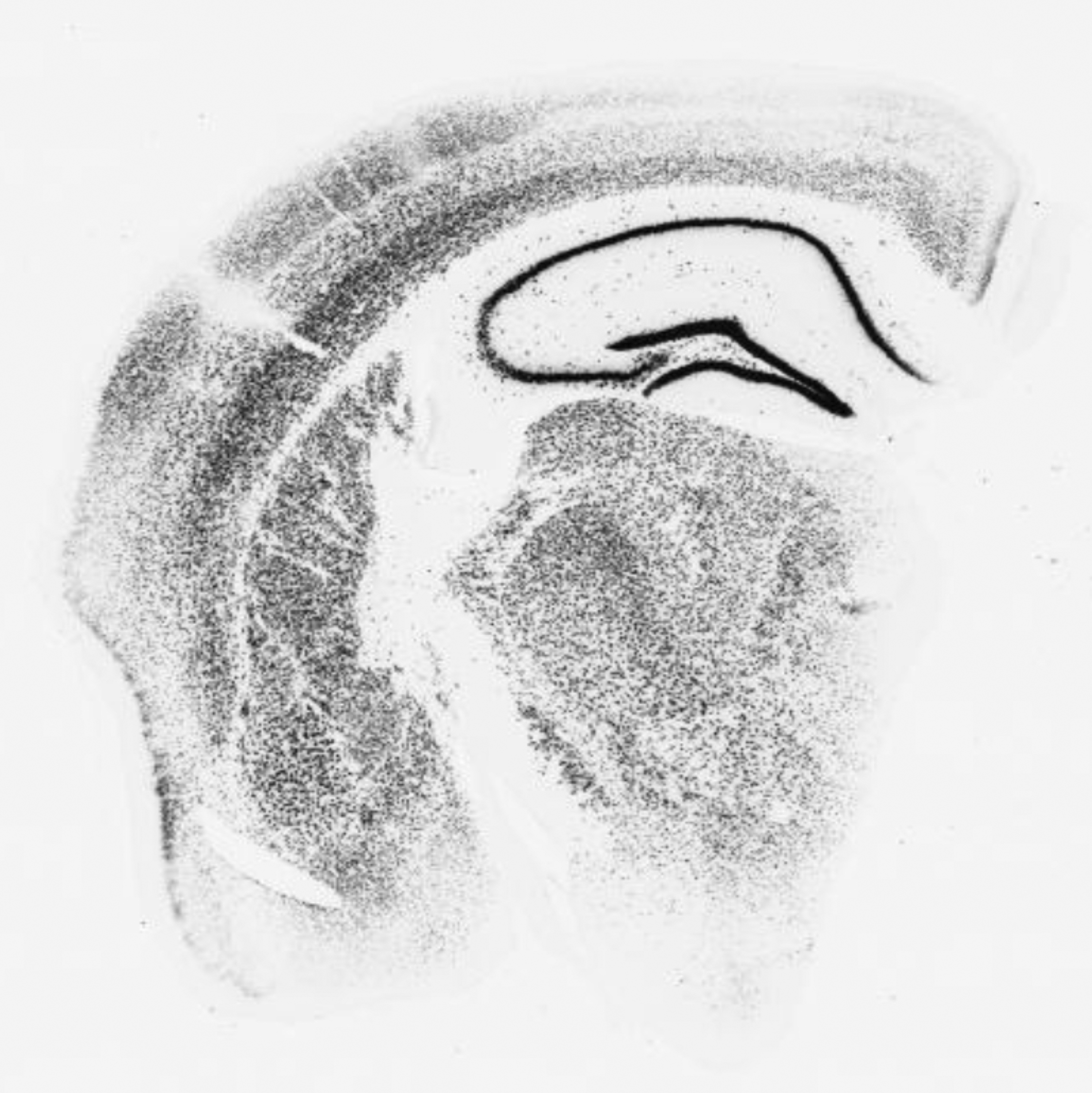

Supplement: Supplementary file 6 — Source Data for Figure 3 [file EMMM-14-e14649-s001.zip › Source data Fig3/Source data Fig3D/KO 7 Rh10 4w - hip_NeuN_15.jpg]

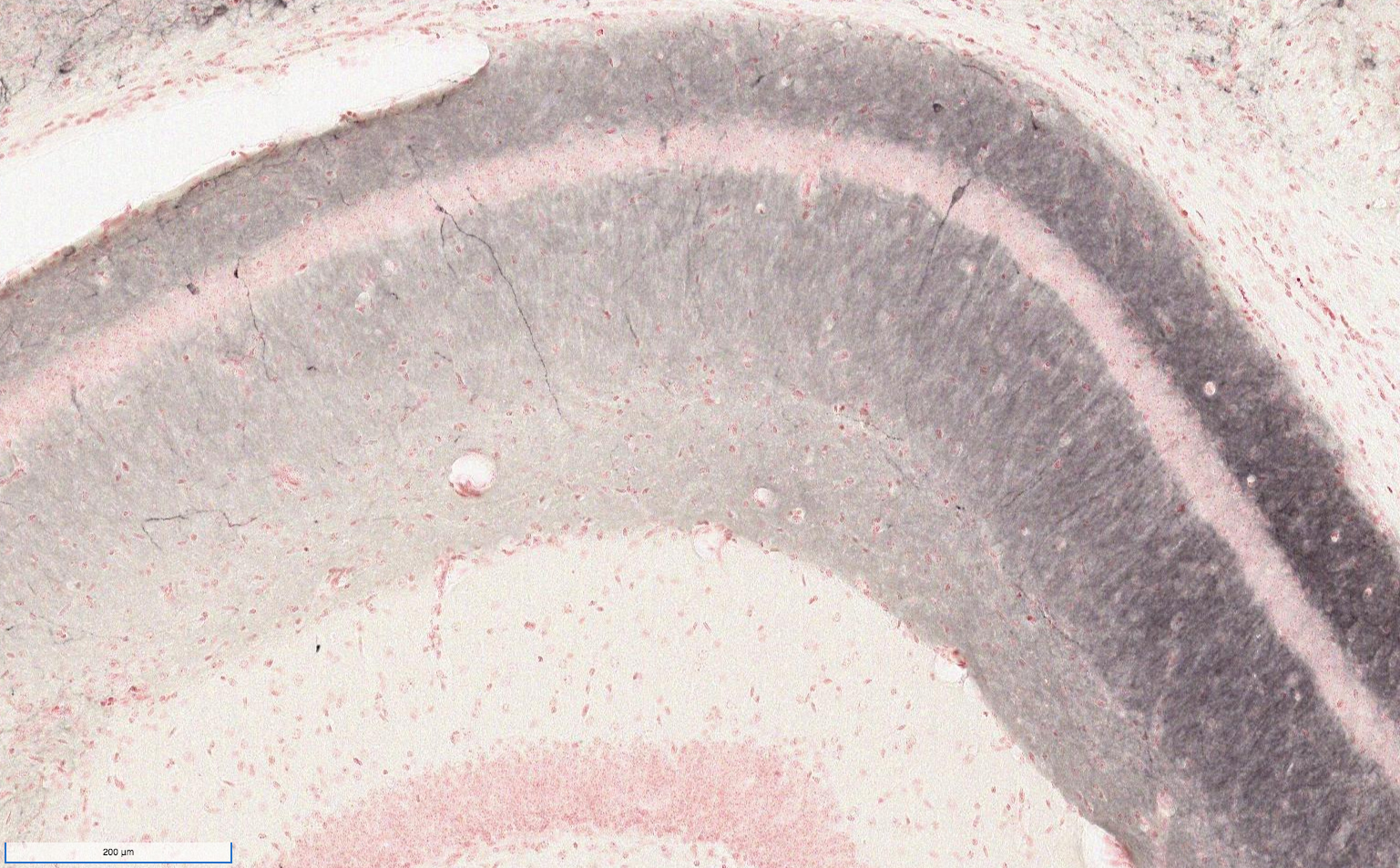

Supplement: Supplementary file 6 — Source Data for Figure 3 [file EMMM-14-e14649-s001.zip › Source data Fig3/Source data Fig3D/Zoom_KHB-1_HA_7K+_15.jpg]

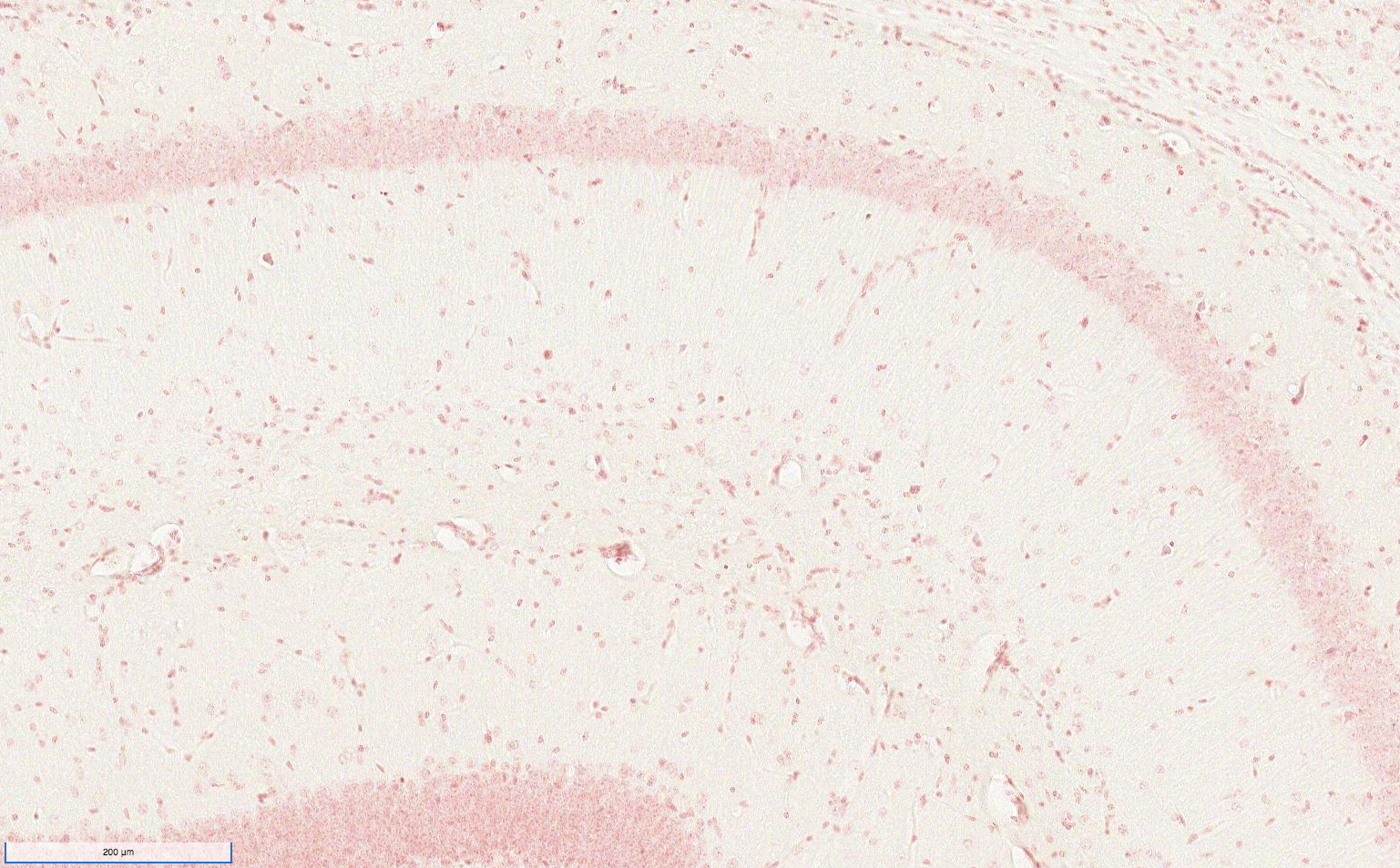

Supplement: Supplementary file 6 — Source Data for Figure 3 [file EMMM-14-e14649-s001.zip › Source data Fig3/Source data Fig3D/Zoom_KHB-1_HA_200K+_15.jpg]

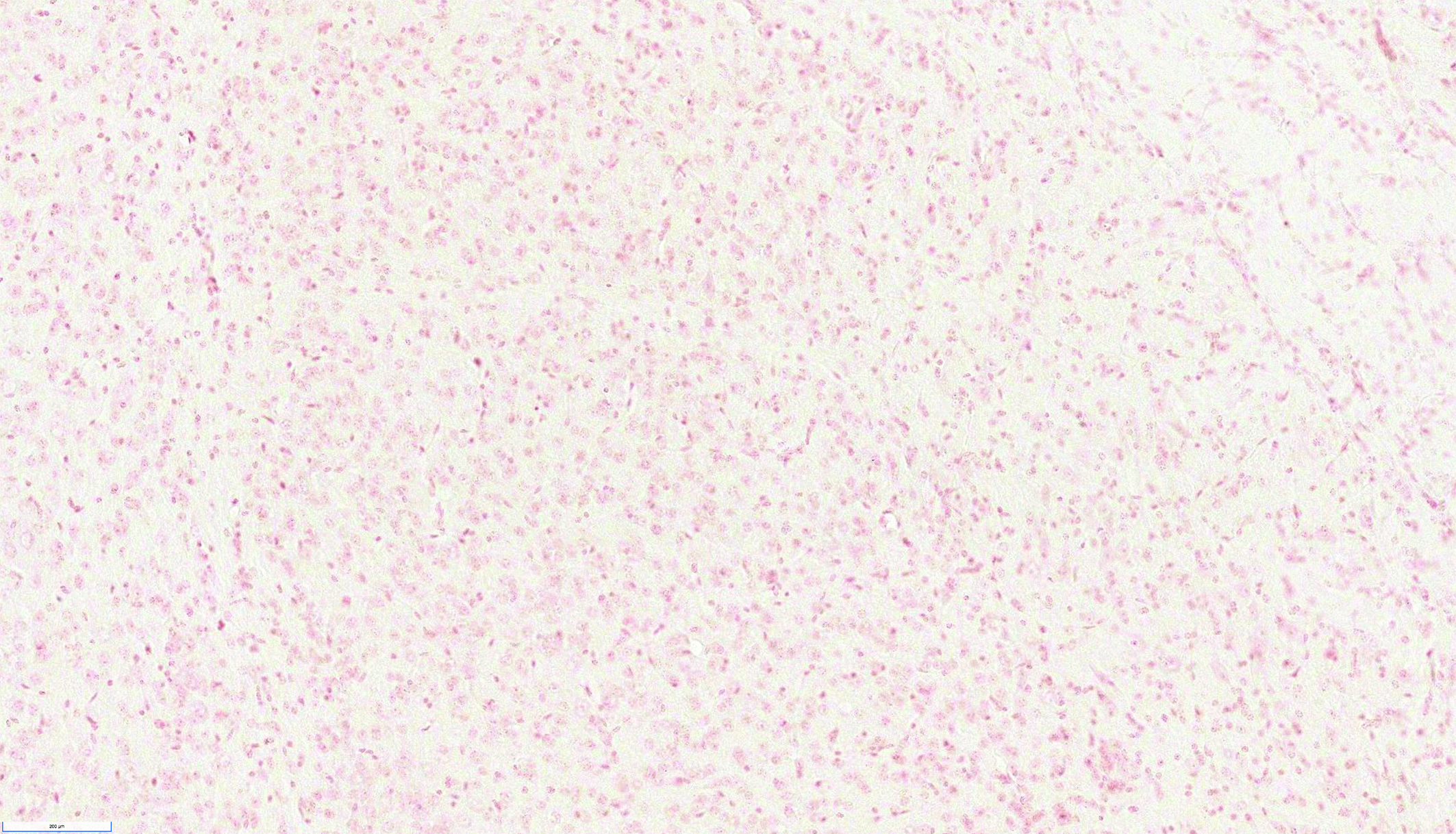

Supplement: Supplementary file 6 — Source Data for Figure 3 [file EMMM-14-e14649-s001.zip › Source data Fig3/Source data Fig3D/Zoom_str-KO 412 vehicle 4w - hip_15.jpg]

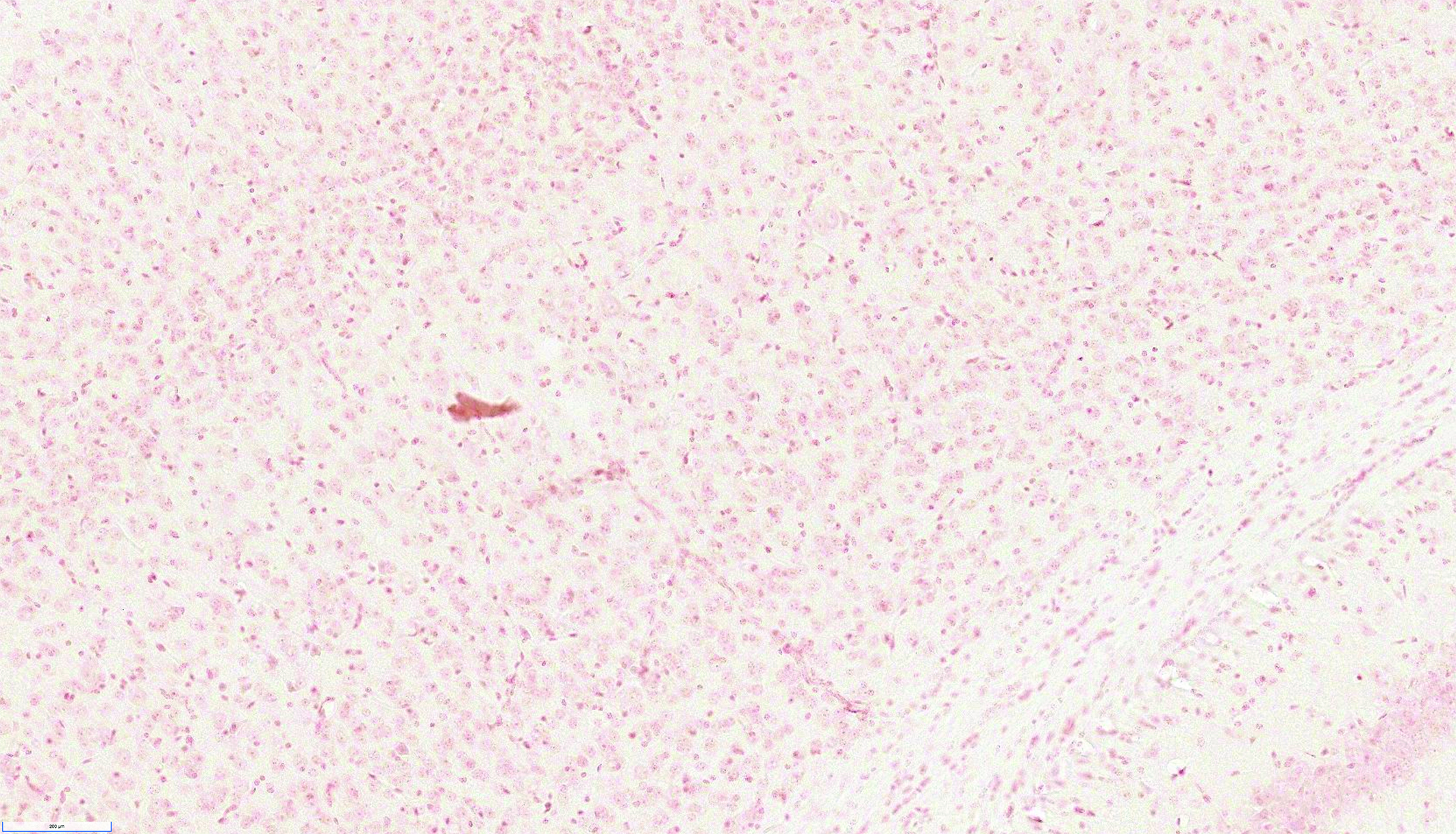

Supplement: Supplementary file 6 — Source Data for Figure 3 [file EMMM-14-e14649-s001.zip › Source data Fig3/Source data Fig3D/Zoom_cor-KO 200 PHPeB 4w - hip_15.jpg]

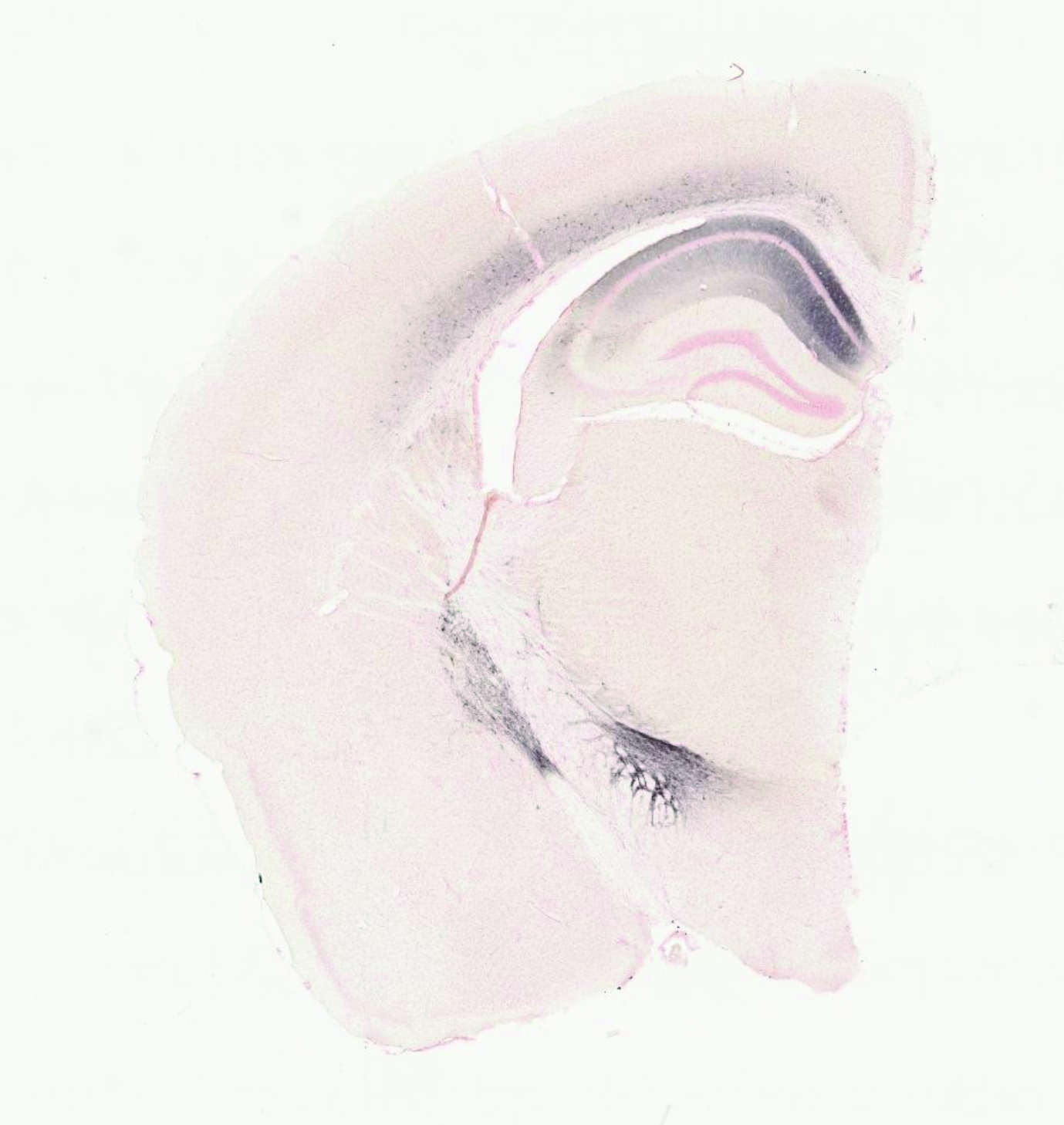

Supplement: Supplementary file 6 — Source Data for Figure 3 [file EMMM-14-e14649-s001.zip › Source data Fig3/Source data Fig3D/KO 7 Rh10 4w - hip_HA_15.jpg]

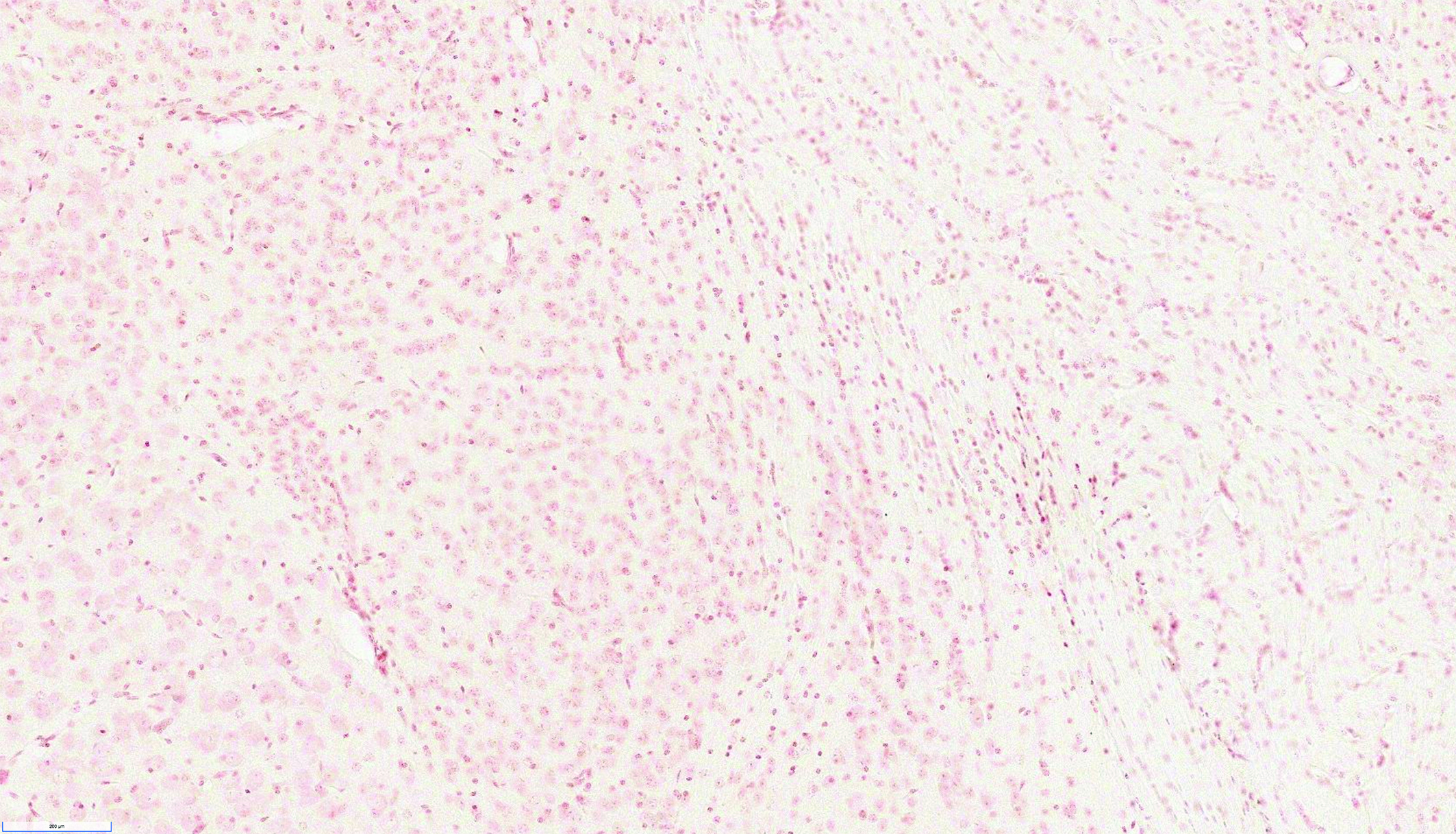

Supplement: Supplementary file 6 — Source Data for Figure 3 [file EMMM-14-e14649-s001.zip › Source data Fig3/Source data Fig3D/Zoom_str-KO 200 PHPeB 4w - hip_15.jpg]

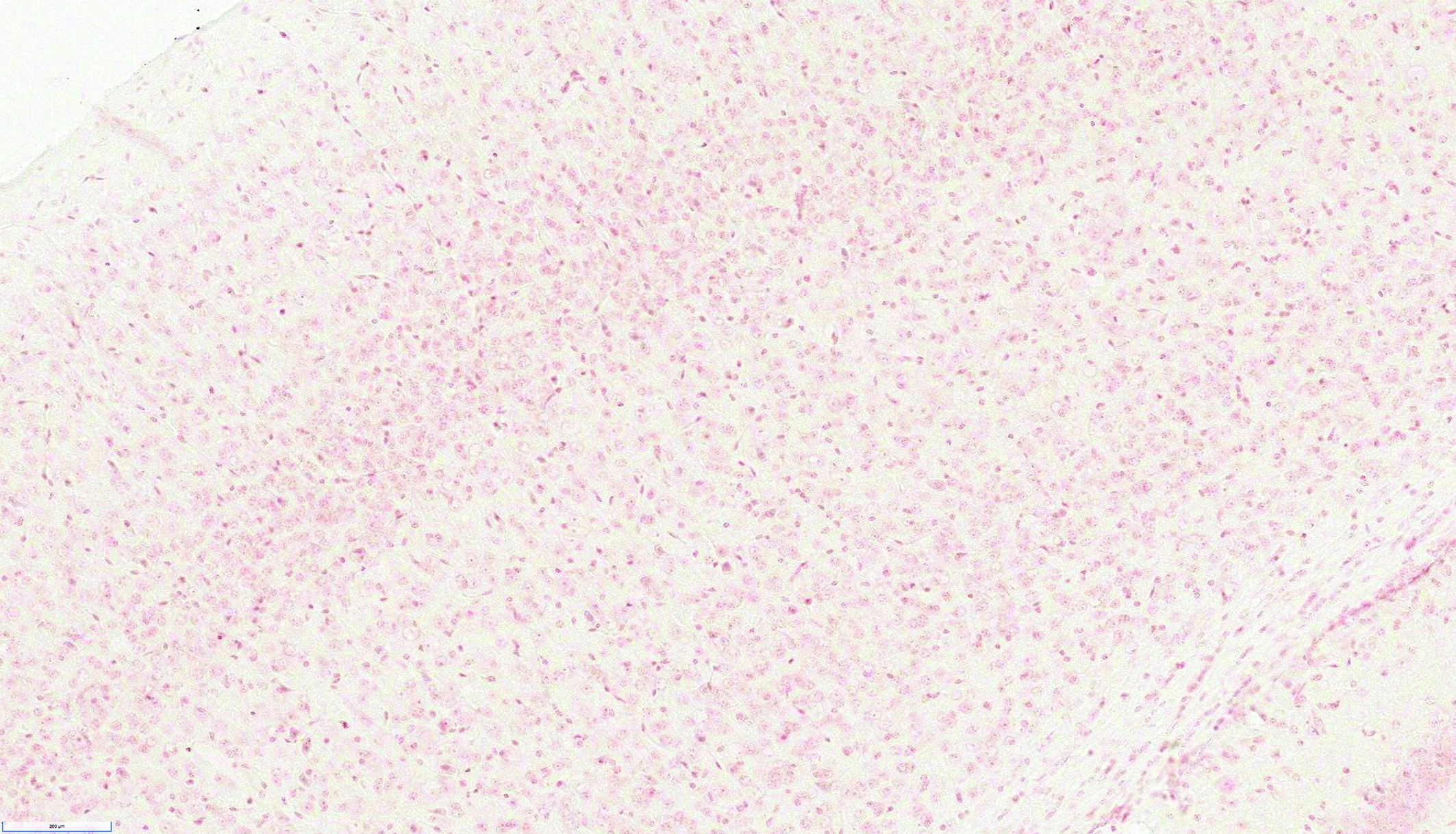

Supplement: Supplementary file 6 — Source Data for Figure 3 [file EMMM-14-e14649-s001.zip › Source data Fig3/Source data Fig3D/Zoom_cor-KO 412 vehicle 4w - hip_15.jpg]

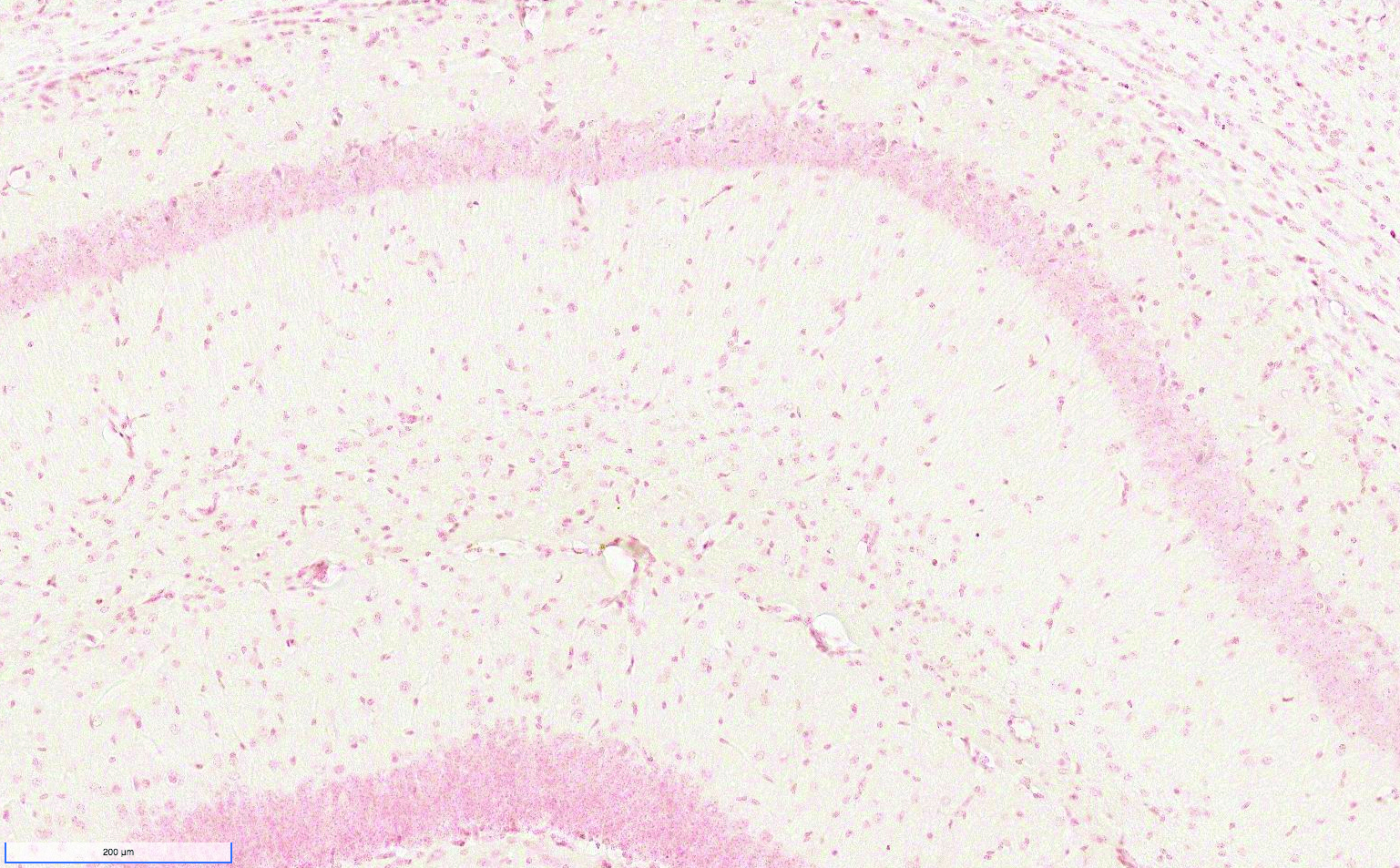

Supplement: Supplementary file 6 — Source Data for Figure 3 [file EMMM-14-e14649-s001.zip › Source data Fig3/Source data Fig3D/Zoom_KHB-1_HA_412K-_15.jpg]
