# Supplementary material for: AAV‐delivered diacylglycerol kinase DGKk achieves long‐term rescue of fragile X syndrome mouse model
Source: EMBO Mol Med. 2022 Apr 4;14(5):e14649. doi: 10.15252/emmm.202114649 (PMC9081908; doi:10.15252/emmm.202114649)
Supplement: Supplementary file 8 [file EMMM-14-e14649-s003.pdf]

EMBO Molecular Medicine

AAV-delivered diacylglycerol kinase DGKk achieves long-term rescue of fragile X syndrome mouse model

karima Habbas, Oktay CAKIL, Boglarka ZAMBO, Ricardos Tabet, Fabrice RIET, Doulaye Dembele, Jean-Louis Mandel, Michaël Hocquemiller, Ralph Laufer, Françoise Piguet, and Hervé MOINE
DOI: 10.15252/emmm.202114649

Corresponding author(s): Hervé MOINE (moine@igbmc.fr)

Review Timeline:

Submission Date:	3rd Jun 21
Editorial Decision:	20th Jul 21
Author Appeal:	5th Aug 21
Editorial Response:	9th Aug 21
Revision Received:	23rd Dec 21
Editorial Decision:	28th Jan 22
Revision Received:	10th Mar 22
Accepted:	11th Mar 22

Editor: Jingyi Hou

Transaction Report:

(Note: With the exception of the correction of typographical or spelling errors that could be a source of ambiguity, letters and reports are not edited. Depending on transfer agreements, referee reports obtained elsewhere may or may not be included in this compilation. Referee reports are anonymous unless the Referee chooses to sign their reports.)

20th Jul 2021

Decision on your manuscript EMM-2021-14649

Dear Dr. MOINE,

Thank you for submitting your work to EMBO Molecular Medicine. We have now received feedback from the three referees who agreed to evaluate your manuscript. As you will see below, the referees raise substantial concerns about your work, which unfortunately preclude its publication in EMBO Molecular Medicine.

While Referee #3 is overall more supportive, Referees #1 and 2 shared overlapping concerns about the missing critical controls, the lack of comparison with the full-length DGKk, and the limited mechanistic insights provided. Referee #1 was also concerned about the technical quality and the medical impact, and Referee #2 expressed additional concerns regarding the overall novelty. In particular, during our pre-decision cross-commenting process (in which the referees are given a chance to make additional comments, including on each other's reports), both Referees #1 and #2 explicitly indicated that they do not support publication of the manuscript in EMBO Molecular Medicine. Considering the substantial points raised and the overall low level of support provided by the reviewers, I am afraid I see little choice but to return the manuscript to you at this point with the decision that we cannot offer to publish it.

I am very sorry that the review of your work did not result in a more favorable outcome on this occasion, but I hope that you will not be discouraged from sending your work to EMBO Molecular Medicine in the future. In any case, thank you for the opportunity to examine this work.

Sincerely,
Jingyi

Jingyi Hou
Editor
EMBO Molecular Medicine

***** Reviewer's comments *****

Referee #1 (Comments on Novelty/Model System for Author):

Some experiments done in mammalian cells lines (e.g. HeLa) would strengthen the paper if done in neurons. Some western blots are of poor quality, which raises question about quantification. The truncation mutant is not compared to full-length, thus reducing the potential value of the medical impact.

Referee #1 (Remarks for Author):

The manuscript by Habbas et al. is a continuation of the 2016 PANAS paper by the same group which showed DGKk's link to Fmrp in FXS. The main point of the current manuscript is that a truncation mutant abolishing Fmrp control over DGKk is a potential novel therapeutic avenue. While this is an interesting approach, I feel there are certain aspects missing to support the claims of the authors.

Major points:

1) A strong confound in the paper is the lack of experimental investigation regarding how ΔN-DGKk compares to full-length DGKk in terms of translation (polysomes, binding to Fmrp), which where done in the 2016 PNAS paper for the full-length protein. Moreover little is shown about the basic biology of DGKk (e.g. subcellular localisation, function) and the focus is only on its link to Fmrp and PA acid. This is concerning, as a truncation mutant may engender off-target effects. I feel a lot of work is required here.

2) Along these lines, treatment with the truncation mutant should be compared to the full-length protein especially in the behavioural assays (4 and 8 weeks), which I can appreciate is a colossal task, yet crucial for proving the value of this therapeutic approach.

3) Fig. 1A: Too small number of postmortem brains. I am surprised that 4 random brains show this result. Was this shown in

other studies of similar tissue? This small number of patients is not convincing. Also, GAPDH is a continuous line, which makes quantification almost impossible. The GAPDH issue is more clear in Figure 2A. These blots need to be repeated and clearly quantified.

Minor comments:
Some of the titles of the results are not descriptive e.g. In vivo correction of phosphatidic acid level in adult mice using multiple routes of administration

I am always sad to see the term "protein translation" in papers. mRNAs are translated and proteins are synthesized.

Referee #2 (Comments on Novelty/Model System for Author):

In this manuscript, Habbas et al. aimed to restore fragile X syndrome (FXS)-associated molecular and cognitive deficits by correcting Diacylglycerol Kinase kappa (DGKk)-dependent functions in the brain. The authors have previously shown that DGKk mRNA is a critical substrate of FMRP (fragile X mental retardation protein) (Tabet et al., 2016, PNAS). In this study, they showed that FMRP regulates DGKk mRNA translation by targeting its N terminal coding region. Truncating the N-terminal region relieved FMRP control of translation on DGKk mRNA. Through series of biochemical experiments, authors showed that ectopically expressing truncated DGKk (ΔN-DGKk) via AVV restored the reduced DGKk protein level in various brain regions of Fmr1 KO mouse brain. Immunostaining data suggest that DGKk levels were restored in brain reasons such as cortex, hippocampus and striatum. The normalization of DGKk in the Fmr1 KO mouse brain led to restoration of phosphatidic acid (PA) in the cortex. Finally, the authors confirmed that the restoration of DGKk significantly improved key behavioral abnormalities associated with FXS.

While the topic of this manuscript is interesting, building on their previous paper in 2016, this current does not provide too much new mechanistic insight. In addition, the study suffers from some major issues that prevent a concrete conclusion to their observations. The lack of key controls further diminishes the confidence about their data. In summary, the study needs to be significantly revised to justify their conclusion. The critiques are as below.

Major:
1. This authors claimed that the truncated DGKk is being regulated independent of FMRP and can be more beneficial. However, the authors' previous publication in 2016 has shown that overexpressing full-length DGKk is able to restore key phenotypes in FXS. It is therefore unclear how much better the truncated DGKk could be without side-by-side comparison with the full-length DGKk. In addition to that, the full-length DGKk is an important positive control for their rescue experiments, and should be included. The pCI only is not a sufficient control. Only with the full-length DGKk, the use of truncated DGKk can be justified.
2. In Fig-4 and the supplemental figures, the authors need to include WT-Rh10 group in all their behavioral assays. Without this critical control, we won't know whether any effects on behavior are FMRP-dependent or not. The authors should include WT-Rh10 and performed two-way ANOVA to detect the genotype (WT vs FXS) and treatment (S vs Rh10) interaction.
3. The conversion of Phosphatidic acid (PA) from DAG is mediated by DGKk. It is shown in Fig. 3E that the PA level is significantly reduced the Fmr1 KO mouse cortex. With DGKk and PA being restored in the cortex in Fmr1-Rh10 group, shouldn't DAG be reduced in Fmr1-Rh10 group (Fig. 3F)? Did the authors consider if alternative metabolic pathways of PA synthesis (such as PA production via Phospholipase D and lysophosphatidic acid acyltransferase pathway) are also affected in Fmr1 KO mice brain?
4. Fmr1-Rh10 mice showed a restoration of body weight compared to the control Fmr1 mice. Has the ΔN-DGKk reinstatement resulted in reduced food intake? What could be the possible mechanism that can explain restored body weight in Fmr1-Rh10 mice?

Minor:
1. Some data were not analyzed with appropriate statistical methods. For example, in Fig 1C-1D, the authors should use one-way ANOVA instead of Student's t-test.
2. Some western blots are over-exposed (Figs. 1C, 1D). The authors should select different representative blots.
3. Some references are not complete and should be corrected.

Referee #2 (Remarks for Author):

In this manuscript, Habbas et al. aimed to restore fragile X syndrome (FXS)-associated molecular and cognitive deficits by correcting Diacylglycerol Kinase kappa (DGKk)-dependent functions in the brain. The authors have previously shown that DGKk mRNA is a critical substrate of FMRP (fragile X mental retardation protein) (Tabet et al., 2016, PNAS). In this study, they showed that FMRP regulates DGKk mRNA translation by targeting its N terminal coding region. Truncating the N-terminal region relieved FMRP control of translation on DGKk mRNA. Through series of biochemical experiments, authors showed that ectopically expressing truncated DGKk (ΔN-DGKk) via AVV restored the reduced DGKk protein level in various brain regions of Fmr1 KO mouse brain. Immunostaining data suggest that DGKk levels were restored in brain reasons such as cortex,

hippocampus and striatum. The normalization of DGKk in the Fmr1 KO mouse brain led to restoration of phosphatidic acid (PA) in the cortex. Finally, the authors confirmed that the restoration of DGKk significantly improved key behavioral abnormalities associated with FXS.

While the topic of this manuscript is interesting, building on their previous paper in 2016, this current does not provide too much new mechanistic insight. In addition, the study suffers from some major issues that prevent a concrete conclusion to their observations. The lack of key controls further diminishes the confidence about their data. In summary, the study needs to be significantly revised to justify their conclusion. The critiques are as below.

Major:
1. This authors claimed that the truncated DGKk is being regulated independent of FMRP and can be more beneficial. However, the authors' previous publication in 2016 has shown that overexpressing full-length DGKk is able to restore key phenotypes in FXS. It is therefore unclear how much better the truncated DGKk could be without side-by-side comparison with the full-length DGKk. In addition to that, the full-length DGKk is an important positive control for their rescue experiments, and should be included. The pCI only is not a sufficient control. Only with the full-length DGKk, the use of truncated DGKk can be justified.
2. In Fig-4 and the supplemental figures, the authors need to include WT-Rh10 group in all their behavioral assays. Without this critical control, we won't know whether any effects on behavior are FMRP-dependent or not. The authors should include WT-Rh10 and performed two-way ANOVA to detect the genotype (WT vs FXS) and treatment (S vs Rh10) interaction.
3. The conversion of Phosphatidic acid (PA) from DAG is mediated by DGKk. It is shown in Fig. 3E that the PA level is significantly reduced the Fmr1 KO mouse cortex. With DGKk and PA being restored in the cortex in Fmr1-Rh10 group, shouldn't DAG be reduced in Fmr1-Rh10 group (Fig. 3F)? Did the authors consider if alternative metabolic pathways of PA synthesis (such as PA production via Phospholipase D and lysophosphatidic acid acyltransferase pathway) are also affected in Fmr1 KO mice brain?
4. Fmr1-Rh10 mice showed a restoration of body weight compared to the control Fmr1 mice. Has the ΔN-DGKk reinstatement resulted in reduced food intake? What could be the possible mechanism that can explain restored body weight in Fmr1-Rh10 mice?

Minor:
1. Some data were not analyzed with appropriate statistical methods. For example, in Fig 1C-1D, the authors should use one-way ANOVA instead of Student's t-test.
2. Some western blots are over-exposed (Figs. 1C, 1D). The authors should select different representative blots.
3. Some references are not complete and should be corrected.

Referee #3 (Comments on Novelty/Model System for Author):

The manuscript is very well and clearly written, and the tables and figures are clearly presented. The findings are very exciting and relevant, and are suited for the scope of EMBO.

Referee #3 (Remarks for Author):

In the study of Habbas et al. "AAV-delivered diacylglycerol kinase DGKk achieves long-term rescue of fragile X syndrome mouse model", the authors show that diacylglycerol kinase kappa (DGKk), when modified as to become FMRP-independent and delivered into the brain of mice using adeno-associated viral vectors, corrects brain diacylglycerol and phosphatidic acid homeostasis. It also corrects main behaviors of the Fmr1 KO mouse. Possibly, this preclinical research can lead to a gene therapy to treat FXS.

The manuscript is very well and clearly written, and the tables and figures are clearly presented. The findings are very exciting and relevant, and are suited for the scope of EMBO.
Please find below my minor comments regarding the paper:

1. Line 101: Why only in cerebellum? What about the other brain areas?

2. Line 110: The authors talk about different species but only tested in mouse and human. Is it tested in other species?

3. Figure 2C: Regarding P-eIF4E, why are the bands in E1 and E3 WT so much lower than E2? You would expect more uniformity?

4. Line 159: Why did you decide to start injecting in 5-week old mice? Why not younger or older?

5. Line 168: Did you also look for expression of ΔN-DGKk in cerebellum?

6. Line 172 and sup Fig. 3B: What is the reason that other brain regions did not show a difference? Please elaborate.

7. Line 184-185: The authors refer to sup Fig. 7J which is not present?

8. Line 193: Normally Fmr1 KO mice show increased anxiety why the authors find the opposite. What is the reason of this?

9. It is not clear what type of Fmr1 KO mouse the authors used. This needs to be specified in materials and methods.

10. Line 223 and Fig. 3C: In the last phase of the social interaction test, the mice should show increased interaction with the novel mouse compared to the previously encountered mouse. It is not clear if that is the case here, and the authors say the KO mouse did not recognize the previously encountered mouse. Please specify the differences and behavior better.

11. Sup Fig. 4G: This should be a main figure due to the differences found in nesting. Though it does not look statistical significant, regarding the error bars. Please explain this further.

As a service to authors, EMBO provides authors with the possibility to transfer a manuscript that one journal cannot offer to publish to another EMBO publication. The full manuscript and if applicable, reviewers reports are automatically sent to the receiving journal to allow for fast handling and a prompt decision on your manuscript. For more details of this service, and to transfer your manuscript to another EMBO title please click on Link Not Available

Please do not share this URL as it will give anyone who clicks it access to your account.

Thank you for your response.

We thank the reviewers for their comments.

We acknowledge that a number of points can be improved in our manuscript and we certainly would do our best to perform the requested corrections. There are however a number of points put forward by reviewers #1 and #2 (notably the lack of comparison with the full-length DGKk, the limited improvements from our previous study and the insufficient mechanistic insights), and on which your editorial rejection decision is mostly based, which we believe are inaccurate due to a possible misinterpretation of the scope of our study.

Our present study is a direct follow up of our initial work (Tabet et al. PNAS 2016), where we demonstrated that biolistic delivery of a N-terminally truncated DGKk transgene in FMR1-KO hippocampal slice cultures can correct the abnormal morphology of neuronal dendritic spines, representing the first demonstration "in vitro" of the functional link between DGKk and FMRP. In our current study, we designed and optimized our transgene cassette expressing ΔN-DGKk for an AAV based approach to enable gene therapy for FXS. We are expressing the same DGKk protein lacking the N-term part in current and past studies. We demonstrated in the current study that this ΔN-DGKk, which is stably expressed, does not require FMRP for its translation and is suitable to be expressed in FXS condition where FMRP expression is severely reduced. Full length protein cannot be detected in absence of FMRP and cannot be used for gene therapy development (also the size of the full-length protein exceeds the limit for packaging it into AAV). In the current study, we demonstrated efficacy "in vivo" of our AAV construct ΔN-DGKk using the most common Fmr1-KO model and we also showed that overexpression of ΔN-DGKk was well tolerated with no overall toxicity on survival, bodyweight or defects measured by any of the behavioral assays done.

The detailed mechanism of how FMRP controls DGKk mRNA translation is certainly a key fundamental question (and we are indeed pursuing it), but here our goal was to establish preclinical proof of concept of a gene therapy approach for Fragile X. The current lack of disease-modifying FXS drugs and the difficulty to use FMRP itself for gene therapy (i.e. 1.5 fold FMRP overexpression causes abnormal behaviors in mouse) urges to test alternative strategies.

The main finding of our study is the demonstration that a modified form of the DGKk enzyme delivered with AAV Rh10 to FXS adolescent mice fully rescues their core behavioral phenotypes. This is to our knowledge the most advanced demonstration of the feasibility of a gene therapy approach for FXS. The range of behavioral tests performed here in a single study is probably one the largest reported for the FXS model, enabling an exhaustive view of the correction range. None of the reviewers questioned the efficacy of the treatment, which is the prime result of our study. Furthermore, the use of an FDA-approved vector for use in children and the ability to administer the treatment at a postdevelopmental age are two essential elements that give to the study a high translatability aspect.

Therefore, based on the fact that we think the goal and impact of our study was misunderstood, we wish to make an appeal on the present decision to reject our study.

Would you be willing to accept this appeal, please find here below our point by point response to the reviewer's questions and comments.

9th Aug 2021

Dear Hervé,

Thank you for your message asking us to reconsider our decision regarding your manuscript EMM-2021-14649. I have carefully read your manuscript and referees' report once again and have discussed your appeal and preliminary point-by-point responses with my colleagues. I have also sought external advice on the study from an expert in the field. Based on the rebuttal letter you provided, we think your responses to the referees' criticism sound reasonable. As such, we would welcome the submission of a revised version for further consideration.

In light of the points raised in the initial reviews and the external advisor's comments, the following issues should be addressed:

- Efforts need to be made to compare the truncated DGKk and the full-length version in an in vitro context. However, an in vivo comparison is not required for the acceptance of the manuscript.
- Referee #2 raised significant concerns about the lack of control experiments in wild-type animals. While we do not require a complete recapitulation of the results in WT animals, some control experiments in WT animals along those lines are needed.
- Referee #1's is concerned about the small number of FXS human brains. The limitations in this regard need to be discussed in writing.
- All other concerns regarding the statistics and technical issues need to be addressed.

Please note that EMBO Molecular Medicine strongly supports a single round of revision. As acceptance or rejection of the manuscript will depend on another round of review, your responses should be as complete as possible.

EMBO Molecular Medicine has a "scooping protection" policy, whereby similar findings published by others during review or revision are not a criterion for rejection. Should you decide to submit a revised version, I do ask you to get in touch after three months if you have not completed it to update us on the status.

We are aware that many laboratories cannot function at full efficiency during the current COVID-19/SARS-CoV-2 pandemic and have therefore extended our "scooping protection policy" to cover the period required for a full revision to address the experimental issues. Please let me know should you need additional time, and also if you see a paper with related content published elsewhere.

Please read below for important editorial formatting and consult our author's guidelines to properly format your revised article for EMBO Molecular Medicine.

I look forward to receiving your revised manuscript.

Use this link to login to the manuscript system and submit your revision: https://embomolmed.msubmit.net/cgi-bin/main.plex

Kind regards,
Jingyi

Jingyi Hou
Editor
EMBO Molecular Medicine

I have included below the comments from the external arbitrating advisor:

"Overall, I agree with the authors in their response to the reviews.

1) While I agree that this work is an extension of their prior more biochemical studies, that extension into FMR1 KO mice with AAV delivery 8 weeks after birth leading to phenotypic corrections represents a significant undertaking and is an important advance for the field. It would seem to me to be a perfect fit for EMBO Mol. Med.

2) I agree that some work (likely in in-vitro systems) comparing the truncated DGKk to the full length DGKk would help alleviate some of Reviewer #1 and #2 concerns, but that should not be required in vivo.

3) The request for some data in control animals with the truncated AAV is a relevant request and I think should have been done, especially if they are proposing this as a preclinical proof of principle. The safety of this approach would be a concern in that setting. However, I do not think a complete recapitulation of their results in WT with the virus is required.

4) Getting samples from even 4 FXS human brains is quite an accomplishment and I think their data there is worthy of inclusion. Acknowledging that it is only 4 brains and the complications of looking at protein expression on autopsy tissues as limitations in the discussion is reasonable.

In sum, while I agree that the paper does require some substantial revisions and additional experiments, I think that overall the results represent a significant enough advance to justify re-consideration in EMBO Mol. Med."

*****

When submitting your revised manuscript, please carefully review the instructions that follow below. We perform an initial quality control of all revised manuscripts before re-review; failure to include requested items will delay the evaluation of your revision.

We require:

1) A .docx formatted version of the manuscript text (including legends for main figures, EV figures and tables). Please make sure that the changes are highlighted to be clearly visible.

2) Individual production quality figure files as .eps, .tif, .jpg (one file per figure). For guidance, download the 'Figure Guide PDF' (https://www.embopress.org/page/journal/17574684/authorguide#figureformat).

3) A .docx formatted letter INCLUDING the reviewers' reports and your detailed point-by-point responses to their comments. As part of the EMBO Press transparent editorial process, the point-by-point response is part of the Review Process File (RPF), which will be published alongside your paper.

4) A complete author checklist, which you can download from our author guidelines (https://www.embopress.org/page/journal/17574684/authorguide#submissionofrevisions). Please insert information in the checklist that is also reflected in the manuscript. The completed author checklist will also be part of the RPF.

5) Please note that all corresponding authors are required to supply an ORCID ID for their name upon submission of a revised manuscript.

6) It is mandatory to include a 'Data Availability' section after the Materials and Methods. Before submitting your revision, primary datasets produced in this study need to be deposited in an appropriate public database, and the accession numbers and database listed under 'Data Availability'. Please remember to provide a reviewer password if the datasets are not yet public (see https://www.embopress.org/page/journal/17574684/authorguide#dataavailability).

In case you have no data that requires deposition in a public database, please state so in this section. Note that the Data Availability Section is restricted to new primary data that are part of this study.

7) For data quantification: please specify the name of the statistical test used to generate error bars and P values, the number (n) of independent experiments (specify technical or biological replicates) underlying each data point and the test used to calculate p-values in each figure legend. The figure legends should contain a basic description of n, P and the test applied. Graphs must include a description of the bars and the error bars (s.d., s.e.m.).

8) We would also encourage you to include the source data for figure panels that show essential data. Numerical data should be provided as individual .xls or .csv files (including a tab describing the data). For blots or microscopy, uncropped images should be submitted (using a zip archive if multiple images need to be supplied for one panel). Additional information on source data and instruction on how to label the files are available at

.

9) Our journal encourages inclusion of *data citations in the reference list* to directly cite datasets that were re-used and obtained from public databases. Data citations in the article text are distinct from normal bibliographical citations and should directly link to the database records from which the data can be accessed. In the main text, data citations are formatted as follows: "Data ref: Smith et al, 2001" or "Data ref: NCBI Sequence Read Archive PRJNA342805, 2017". In the Reference list, data citations must be labeled with "[DATASET]". A data reference must provide the database name, accession number/identifiers and a resolvable link to the landing page from which the data can be accessed at the end of the reference. Further instructions are available at .

10) We replaced Supplementary Information with Expanded View (EV) Figures and Tables that are collapsible/expandable online. A maximum of 5 EV Figures can be typeset. EV Figures should be cited as 'Figure EV1, Figure EV2" etc... in the text and their respective legends should be included in the main text after the legends of regular figures.

- For the figures that you do NOT wish to display as Expanded View figures, they should be bundled together with their legends in a single PDF file called *Appendix*, which should start with a short Table of Content. Appendix figures should be referred to in the main text as: "Appendix Figure S1, Appendix Figure S2" etc.

- Additional Tables/Datasets should be labeled and referred to as Table EV1, Dataset EV1, etc. Legends have to be provided in a separate tab in case of .xls files. Alternatively, the legend can be supplied as a separate text file (README) and zipped together with the Table/Dataset file.

See detailed instructions here:

.

11) The paper explained: EMBO Molecular Medicine articles are accompanied by a summary of the articles to emphasize the major findings in the paper and their medical implications for the non-specialist reader. Please provide a draft summary of your article highlighting

- the medical issue you are addressing,

- the results obtained and

- their clinical impact.

This may be edited to ensure that readers understand the significance and context of the research. Please refer to any of our published articles for an example.

12) For more information: There is space at the end of each article to list relevant web links for further consultation by our readers. Could you identify some relevant ones and provide such information as well? Some examples are patient associations, relevant databases, OMIM/proteins/genes links, author's websites, etc...

13) Author contributions: the contribution of every author must be detailed in a separate section (before the acknowledgments).

14) A Conflict of Interest statement should be provided in the main text.

15) Every published paper now includes a 'Synopsis' to further enhance discoverability. Synopses are displayed on the journal webpage and are freely accessible to all readers. They include a short stand first (maximum of 300 characters, including space) as well as 2-5 one-sentences bullet points that summarizes the paper. Please write the bullet points to summarize the key NEW findings. They should be designed to be complementary to the abstract - i.e. not repeat the same text. We encourage inclusion of key acronyms and quantitative information (maximum of 30 words / bullet point). Please use the passive voice. Please attach these in a separate file or send them by email, we will incorporate them accordingly.

Please also suggest a striking image or visual abstract to illustrate your article as a PNG file 550 px wide x 300-600 px high.

EMBO Molecular Medicine has a "scooping protection" policy, whereby similar findings that are published by others during review or revision are not a criterion for rejection. Should you decide to submit a revised version, I do ask that you get in touch after three months if you have not completed it, to update us on the status.

***** Reviewer's comments *****

Detailed point-by-point responses to reviewers
(Referees' comments in balck, Authors' responses in blue)

Referee #1 (Comments on Novelty/Model System for Author):

Some experiments done in mammalian cells lines (e.g. HeLa) would strengthen the paper if done in neurons.
The main goal of doing experiments in two different mammalian cell lines was first to demonstrate the recapitulation of FMRP control on DGKk transgene in a non-neuronal context, then, to perform site directed mutagenesis analyses on this transgene in order to define the region of DGKk involved in control. Neuronal cultures are not appropriate for performing such kind of gene reporter assays. We have developed an AAV vector expressing ΔN-DGKk that enables high transduction rate in neurons, however for full length DGKk, its ORF size exceeds the encapsidation size-limit of an AAV vector and is thus not usable. We believe that the recapitulation of endogenous FMRP control on a DGKk transgene (DGKk is not expressed in non-neuronal cells) is an even better argument of the robustness of the control mechanism. Concerning the other assays of ΔN-DGKk impact on cell signaling and toxicity, this was done in neurons.

Some western blots are of poor quality, which raises question about quantification.
We have replaced the western blots figures that were judged unsatisfactory.

The truncation mutant is not compared to full-length, thus reducing the potential value of the medical impact.
We have performed new experiments to compare full-lenth DGKk with its ΔN-DGKk truncation in cells, including:
- immunofluorescence data showing same localization of the two proteins at plasma membrane in Hela cells, contrarily to a protein that lack DAG binding domain and show a diffuse localization within cytoplasm (Fig2A),
- western blot data showing similar impact of the two proteins on global protein synthesis rate measure by puromycin labeling (Fig2B),
- western blot data showing similar impact of the two proteins on eIF4E and mTOR signaling (Fig EV2B).

Referee #1 (Remarks for Author):

The manuscript by Habbas et al. is a continuation of the 2016 PANAS paper by the same group which showed DGKk's link to Fmrp in FXS. The main point of the current manuscript is that a truncation mutant abolishing Fmrp control over DGKk is a potential novel therapeutic avenue. While this is an interesting approach, I feel there are certain aspects missing to support the claims of the authors.

Major points:

1) A strong confound in the paper is the lack of experimental investigation regarding how ΔN-DGKk compares to full-length DGKk in terms of translation (polysomes, binding to Fmrp), which where done in the 2016 PNAS paper for the full-length protein.
The understanding of the mechanism of mRNA control by FMRP is a burning question since over 20 years when FMRP had been identified as an mRNA binding protein. The mechanistic details of how FMRP controls DGKk mRNA translation in this context is a key fundamental question, but this question is beyond the scope of this present study, because our goal here was to demonstrate preclinical proof of concept of a gene therapy approach for Fragile X.

Moreover little is shown about the basic biology of DGKk (e.g. subcellular localisation, function) and the focus is only on its link to Fmrp and PA acid.

We fully agree that the basic biology of DGKk is an important question. In fact, the phenotypes of the *Dgkk*-KO mouse recently characterized in our laboratory are providing compelling evidence of a strong overlap with *Fmr1*-KO phenotypes, giving further support to the key role of DGKk in FXS pathology. We would be willing to share these data with the editor if requested. For the present report we believe however that a detailed characterization of DGKk function is outside the scope of the present study.

This is concerning, as a truncation mutant may engender off-target effects. I feel a lot of work is required here.
We understand the concern of reviewer#1 as this was our main question all along this work and in fact our study was mainly devoted to define the extent to which ΔN-DGKk enzyme can compensate for FMRP absence and replace full-length DGKk.
We show in this work that the truncated ΔN-DGKk does not cause undesirable effects in neurons in vitro and in vivo. The N-ter domain truncation in DGKk enzyme has previously been reported to keep its normal enzymatic function (Imai et al. 2005), and we show that strong expression of DGKk in neurons has no detectable toxic effects (Fig. EV2CF). We have added new set of data showing that ΔN-DGKk has similar impact than full-length DGKk on cell signaling (Fig. 2B, Fig. EV2AB and see comments above). Furthermore, we have added new set of data showing that ΔN-DGKk administration in WT mouse does not cause visible effects on a wide battery of behavioral tests (Table EV2, Fig. EV4A), which is a good indication of an absence of undesired effects.

2) Along these lines, treatment with the truncation mutant should be compared to the full-length protein especially in the behavioural assays (4 and 8 weeks), which I can appreciate is a colossal task, yet crucial for proving the value of this therapeutic approach.
The reason to repeat all the behavioral rescue experiments with full length protein to prove value of therapeutic value of the approach appears unclear. We did not perform these experiments for three reasons: 1) in our present study, we provide data that full length DGKk requires FMRP to be expressed from a transgene in a cell-based system and that DGKk is almost absent in Fragile X patients brain (while DGKk mRNA is normally expressed in absence of FMRP). Thus, there would be no point in treating *Fmr1*-KO animals with a full-length transgene that will most likely not be able to express DGKK in absence of FMRP, as demonstrated in cells, 2) full-length DGKK protein cannot be expressed using an AAV vector because its ORF exceeds the encapsidation size limit of AAV virus.
3) Contrarily to what seems to be assumed by reviewer, in our initial study (PNAS 2016) we used a truncated ΔN-DGKk, not a full-length to perform rescue experiments of *Fmr1*-KO dendritic spine morphology of hippocampal slice in vitro cultures. Indeed, prior to 2016 (i.e. before the release of genome annotation GRCm39), mouse DGKk gene was incorrectly annotated. mDGKk in GRCm38 corresponded to a "ΔN-DGKk" protein. Thus, our initial rescue experiments were done with a truncated DGKk protein. This point is now stated in the ms.

3) Fig. 1A: Too small number of postmortem brains. I am surprised that 4 random brains show this result. Was this shown in other studies of similar tissue? This small number of patients is not convincing. Also, GAPDH is a continuous line, which makes quantification almost impossible. The GAPDH issue is more clear in Figure 2A. These blots need to be repeated and clearly quantified.
Access to human brain postmortem samples is not easy. Ethical approval in France is a big issue, and access to samples of sufficiently good quality to quantitatively detect DGKk is even more complicated. In addition to the presented samples, we also tested samples from another Biobank, but we could not detect DGKk in these samples (WT as well as FXS). According to our prior experience, brain samples need to be frozen as quickly as possible to be able to detect DGKk, as DGKk is a large protein extremely prone to degradation. Moreover, the brain samples we used are high-quality samples received from Dr Tassone, who kindly shared these samples with us. We also tested four FXS premutation samples that showed similar bands as in unaffected control (these results can be seen in the source data files for Fig1). We used cerebellum samples, because among

the various samples tested (cerebellum, cortex, striatum) we were able to detect DGKk only in cerebellum, this is in agreement with the fact that DGKk is transcribed at 10-fold higher level in this tissue (our qRT-PCR and Gtex portalhttps://gtexportal.org/home/gene/DGKK).
New Blots with better quality were added.

Minor comments:
Some of the titles of the results are not descriptive e.g. In vivo correction of phosphatidic acid level in adult mice using multiple routes of administration
Reviewer is correct, we replaced title with: *ΔN-DGKk corrects cortical phosphatidic acid level in Fmr1-KO adult mice*
I am always sad to see the term "protein translation" in papers. mRNAs are translated and proteins are synthesized.
We thank the reviewer for this remark, and we corrected this abuse of language.

Referee #2 (Comments on Novelty/Model System for Author):

In this manuscript, Habbas et al. aimed to restore fragile X syndrome (FXS)-associated molecular and cognitive deficits by correcting Diacylglycerol Kinase kappa (DGKk)-dependent functions in the brain. The authors have previously shown that DGKk mRNA is a critical substrate of FMRP (fragile X mental retardation protein) (Tabet et al., 2016, PNAS). In this study, they showed that FMRP regulates DGKk mRNA translation by targeting its N terminal coding region. Truncating the N-terminal region relieved FMRP control of translation on DGKk mRNA. Through series of biochemical experiments, authors showed that ectopically expressing truncated DGKk (ΔN-DGKk) via AVV restored the reduced DGKk protein level in various brain regions of Fmr1 KO mouse brain. Immunostaining data suggest that DGKk levels were restored in brain reasons such as cortex, hippocampus and striatum. The normalization of DGKk in the Fmr1 KO mouse brain led to restoration of phosphatidic acid (PA) in the cortex. Finally, the authors confirmed that the restoration of DGKk significantly improved key behavioral abnormalities associated with FXS.

While the topic of this manuscript is interesting, building on their previous paper in 2016, this current does not provide too much new mechanistic insight. In addition, the study suffers from some major issues that prevent a concrete conclusion to their observations. The lack of key controls further diminishes the confidence about their data. In summary, the study needs to be significantly revised to justify their conclusion. The critiques are as below.

Major:
1. This authors claimed that the truncated DGKk is being regulated independent of FMRP and can be more beneficial. However, the authors' previous publication in 2016 has shown that overexpressing full-length DGKk is able to restore key phenotypes in FXS. It is therefore unclear how much better the truncated DGKk could be without side-by-side comparison with the full-length DGKk. In addition to that, the full-length DGKk is an important positive control for their rescue experiments, and should be included. The pCI only is not a sufficient control. Only with the full-length DGKk, the use of truncated DGKk can be justified.

As stated already above, in our initial study (Tabet et al., PNAS 2016), we used a truncated ΔN-DGKk, not the full-length protein. We have added a clear statement about this issue in the present ms.
For the several reasons indicated above (see comments to reviewer #1), full-length DGKk cannot be used as a positive control in vivo because full-length DGKk 1) needs FMRP for its efficient expression and 2) cannot be expressed from an AAV vector (exceeds AAV encapsidation size limit). As for the restored “key phenotypes in FXS”, our previous Tabet et al. 2016 study solely provided in vitro demonstration that ΔN-DGKk can correct the abnormal dendritic spine morphology, which is far from showing complex behavioral rescue in grown-up animals as shown here. Another important piece of

evidence of the rescuing ability of ΔN-DGKk in vivo is the correction of brain lipidomic profile, which is a strong molecular basis to the hypothesis model of DGKk deregulation in FXS.
As stated already above, we have added new data supporting the comparison of cellular properties of full-length DGKk vs ΔN-DGKk in vitro.

2. In Fig-4 and the supplemental figures, the authors need to include WT-Rh10 group in all their behavioral assays. Without this critical control, we won't know whether any effects on behavior are FMRP-dependent or not. The authors should include WT-Rh10 and performed two-way ANOVA to detect the genotype (WT vs FXS) and treatment (S vs Rh10) interaction.

The reviewer raises the necessity to repeat all the rescue experiments with AAV-Rh10-ΔN-DGKk in WT animals. Gene therapy applications are usually not intended to treat healthy individuals because this will result into a protein overexpression situation. We acknowledge yet the interest of these experiments for evaluating specificity of the treatment and its potential toxicity in case of overdosing. We have added a new series of experiment on a *Fmr1*-WT cohort with vector AAVRh10-ΔN-DGKk (WT-Rh10) or its saline vehicle control (WT-S), n=10. Five-week-old WT mice were treated in the same conditions as before (hippocampal+striatal injections) and tested four weeks after. ΔN-DGKk expression in WT brain was similar to that of KO animals (western blot, Fig EV3B). Measure of 27 clinical parameters using SHIRPA test to assess general health and basic sensory motor functions of animals indicated that WT-Rh10 mice are not distinguishable from WT-S. Measures of activity in open field arena (using the same setup as for previous *Fmr1*-KO analyses for the Novel Object Recognition tests) showed that locomotor activity (hyperactivity) and time spent in center of arena (anxiety) were not different between the two groups (of note hyperactivity faced to novelty was the most pronounced and robust effect we observed in *Fmr1*-KO mice). This new set of experiments shows that the effect of the AAV treatment is specific of the *Fmr1*-KO genotype and not due to off-target effect.

3. The conversion of Phosphatidic acid (PA) from DAG is mediated by DGKk. It is shown in Fig. 3E that the PA level is significantly reduced the Fmr1 KO mouse cortex. With DGKk and PA being restored in the cortex in Fmr1-Rh10 group, shouldn't DAG be reduced in Fmr1-Rh10 group (Fig. 3F)? Did the authors consider if alternative metabolic pathways of PA synthesis (such as PA production via Phospholipase D and lysophosphatidic acid acyltransferase pathway) are also affected in Fmr1 KO mice brain?

In fact, a failure of DAG to PA conversion following DGKk activity decrease is rather expected to cause an increase of DAG in Fmr1-saline group. Such a DAG increase has been observed in isolated *Fmr1*-KO neuron cultures (Tabet et al. 2016). We do not have definitive explanation why such an increase is not seen in the brain of 15 weak old mice. Several explanations can be advanced such as metabolic compensations “buffering” the increase of DAG in *Fmr1*-KO, or variability between individuals hid the existing difference. Generally, DAG to PA conversion is exclusively controlled by the various DGK enzymes but as stated by reviewer, DAG and PA can be individually produced and converted by alternative routes whose buffering retro-control in FXS and even normal situation is currently unknown. This point has been discussed in the Discussion section.

4. Fmr1-Rh10 mice showed a restoration of body weight compared to the control Fmr1 mice. Has the ΔN-DGKk reinstatement resulted in reduced food intake? What could be the possible mechanism that can explain restored body weight in Fmr1-Rh10 mice?

This is an interesting point. A reduced food intake upon ΔN-DGKk treatment could indeed have been the underlying cause of body weight restoration, suggesting this could be the result of an unspecific correction. In fact, no significant change in food intake was observed (consumption of food pellet has been measured during the actimetry test and these data were added in Fig EV5H). We can only offer

speculation at this stage that a deregulation of DAG and PA signaling is affecting growth and we added comment about this issue in discussion.

Minor:
1. Some data were not analyzed with appropriate statistical methods. For example, in Fig 1C-1D, the authors should use one-way ANOVA instead of Student's t-test.
In Fig1C, comparison was made between siC vs siFMR1 condition. Untransfected condition (“NT”) shown in the figure as negative control was not initially intended to be directly compared with transfection condition (hence the initial choice of a Student's t-test rather than an ANOVA). One way ANOVA was applied to satisfy reviewer’ comment and this had no significant impact on the P-value. In Fig1D, we totally agree that an ANOVA should have been used here and we modified accordingly the Source data and figure legend (P-values did not change $p<0.0001$).

2. Some western blots are over-exposed (Figs. 1C, 1D). The authors should select different representative blots.
New blots have been added.

3. Some references are not complete and should be corrected.
Uncomplete references have been corrected.

Referee #3 (Comments on Novelty/Model System for Author):

The manuscript is very well and clearly written, and the tables and figures are clearly presented. The findings are very exciting and relevant, and are suited for the scope of EMBO.

Referee #3 (Remarks for Author):

In the study of Habbas et al. "AAV-delivered diacylglycerol kinase DGKk achieves long-term rescue of fragile X syndrome mouse model", the authors show that diacylglycerol kinase kappa (DGKk), when modified as to become FMRP-independent and delivered into the brain of mice using adeno-associated viral vectors, corrects brain diacylglycerol and phosphatidic acid homeostasis. It also corrects main behaviors of the Fmr1 KO mouse. Possibly, this preclinical research can lead to a gene therapy to treat FXS.

The manuscript is very well and clearly written, and the tables and figures are clearly presented. The findings are very exciting and relevant, and are suited for the scope of EMBO.
We thank the reviewer for his/her appreciative remarks.

Please find below my minor comments regarding the paper:

1. Line 101: Why only in cerebellum? What about the other brain areas?
Among the various samples tested (cerebellum, cortex, striatum) we were able to detect DGKk only in cerebellum in agreement with a relatively higher expression in this region. Other regions are expected to show higher DGKk level (e.g. hypothalamus) but we have not been able to access to such human material yet. Also, it should be mentioned that is not necessary in the tissue where DGKk is the most expressed that the effect of its absence will be the most deleterious (e.g. variable compensatory effect by other DGK isoenzymes whose level is also varying across regions can be playing).

2. Line 110: The authors talk about different species but only tested in mouse and human. Is it tested in other species?

We tested and evidenced a control of FMRP only on human and mouse DGKk. This suggests that control is conserved at least in mammals. Presence of DGKk gene is conserved down to fish suggesting that the control mechanism could be conserved in these other species but this remains to be determined. We rephrased our description to avoid confusion. "Human DGKk (hDGKk) level is also affected by FMRP knock-down, indicating that FMRP control is conserved between mouse and human"

3. Figure 2C: Regarding P-eIF4E, why are the bands in E1 and E3 WT so much lower than E2? You would expect more uniformity?
We find phosphorylation levels of eIF4E subject to high variability across different neuronal cultures. This variability could reflect biological variability across different embryos or different level of neuronal activation in between different cultures.

4. Line 159: Why did you decide to start injecting in 5-week old mice? Why not younger or older?
5-week-old in mouse corresponds to about an equivalent age of 14 years in humans (i.e. adolescence), which we think is an ideal age to test proof of concept of gene therapy for intellectual disability. Younger ages could have been meaningful too, but at 4-week stereotaxic injections and anesthesia are more difficult to finely control (at 5 weeks no animal loss was observed with over 200 injected mice). P0 injections are easily done and enable wide brain coverage of the AAV when blood brain barrier is not yet closed, but have no human translatability (corresponding to prenatal stage).

5. Line 168: Did you also look for expression of ΔN-DGKk in cerebellum?
We did not look for expression of ΔN-DGKk in cerebellum because we did not expect the AAV to efficiently reach this region from the hippocampus and striatum injection areas.

6. Line 172 and sup Fig. 3B: What is the reason that other brain regions did not show a difference? Please elaborate.
We have no explanation yet for this observation. Either the difference is only present in cortex (for instance because in the other areas a compensation for DGKk absence exists by the other DGK isozymes) or some differences exist also in other areas but have been hidden due to higher variability in these other areas due to fact these other regions are more heterogenous in neuronal composition. Also, we have not tested all brain subregions. The use of 2D mass-spectrometry imaging by spatially defining the DAG and PA level could help resolve this question, but this is outside the scope of this study. Additional discussion was added. "Loss of DGKk activity is expected to cause cellular DAG excess and PA lack. While an excess of DAG has been seen in dissociated *Fmr1*-KO cortical neuron cultures (Tabet et al., 2016), such excess was not observed in the three brain region homogenates analyzed at 15 weeks of age. PA, instead, was clearly seen diminished but only in cortex homogenates. While we have no definitive explanation for these observations, metabolic buffering compensations (including from the other DGK isozymes) may exist to counteract an increase of DAG in *Fmr1*-KO, alternatively, the fact that *DGKk* is one of the least expressed gene of the DGK family (https://gtexportal.org/home/gene/DGKK) and its exquisite expression in restricted neuron populations indicated by spatially resolved transcriptomics (Hu *et al*, 2021) might have hindered the measures in the brain homogenates. "

7. Line 184-185: The authors refer to sup Fig. 7J which is not present?
We apologize, in line 184-185 "sup Fig. 7J" should have been written "sup Fig. 3J"
the corresponding figure appears now as Fig EV3K

8. Line 193: Normally Fmr1 KO mice show increased anxiety why the authors find the opposite. What is the reason of this?
In fact, contradictory results have been published on *Fmr1*-KO mice anxiety. Report of lower anxiety-like behaviors (e.g. Peier et al. 2000; Veeraragavan et al. 2012) and higher (e.g. Restivo et al. 2005).

The “elevated-plus maze (EPM)” is usually the preferred test to investigate anxiety-like phenotypes. However, similarly to the above-mentioned discrepancies, decrease (Yuskaitis et al. 2010) and increase (Bilousova et al. 2009) in anxiety have also been reported during the EPM task when compared with control animals. Because *Fmr1* KO mice are hyperactive (e.g. Peier et al. 2000; Chen and Toth 2001; Yan et al. 2004; Restivo et al. 2005; Olmos-Serrano et al. 2011), it is however most likely that the apparent decrease of anxiety of *Fmr1*-KO mice are biased by hyperactivity, these assay being highly influenced by locomotor activity. This point is now discussed in ms.

9. It is not clear what type of Fmr1 KO mouse the authors used. This needs to be specified in materials and methods.

Absolutely correct and we apologize for this omission. The mouse is the second *Fmr1*-KO mouse model, so called *Fmr1*-KO2 from Mientjes et al. (2006). This has been corrected in MATERIAL AND METHODS, Animal model section.

10. Line 223 and Fig. 3C: In the last phase of the social interaction test, the mice should show increased interaction with the novel mouse compared to the previously encountered mouse. It is not clear if that is the case here, and the authors say the KO mouse did not recognize the previously encountered mouse. Please specify the differences and behavior better.

Indeed, in the second phase of the social interaction, WT-vehicle mice showed preference for novel mouse compared to previously encountered mouse (65±2% significantly different than chance $p<0.0001$) while *Fmr1*-KO-vehicle mice were not significantly different than chance and significantly different than WT-vehicle mice ($p=0.0168$) . *Fmr1*-KO-Rh10-ΔN-DGKk mice were not significantly different than WT-vehicle mice and significantly different than chance ($p=0.019$), indicating a rescuing effect of treatment. Additional explanations have been added to better explain the differences in ms.

11. Sup Fig. 4G: This should be a main figure due to the differences found in nesting. Though it does not look statistical significant, regarding the error bars. Please explain this further.

We described the observation of reduced nest building of Fmr1-KO animals in Rh10 4-Weeks and its possible correction in treated group because WT-S is statistically different from Fmr1-KO but not from Fmr1-S (Fig EV4H). Because of the relative weight we put on the nesting phenotype (some Fmr1-KO groups showed this phenotype while others not, see table EV3), we wish to keep these data in Expended view figures.

28th Jan 2022

Dear Dr. MOINE,

Thank you for the submission of your revised manuscript to EMBO Molecular Medicine. We have now received the enclosed report from the two referees who were asked to re-assess it. As you will see from the comments below, both Referee #3 (who had reviewed the manuscript before) and Referee #4 (who provided advice on the previous study as an external arbitrating advisor) are overall supportive. I am pleased to inform you that we will be able to accept your manuscript pending the following amendments:

1. Please address the remaining minor concerns of Referee #4.

On a more editorial level, please do the following:

I look forward to reading a new revised version of your manuscript soon.

Kind regards,
Jingyi

Jingyi Hou
Editor
EMBO Molecular Medicine

*** Instructions to submit your revised manuscript ***

*** PLEASE NOTE *** As part of the EMBO Publications transparent editorial process initiative (see our Editorial at https://www.embopress.org/doi/pdf/10.1002/emmm.201000094), EMBO Molecular Medicine will publish online a Review Process File to accompany accepted manuscripts.

In the event of acceptance, this file will be published in conjunction with your paper and will include the anonymous referee reports, your point-by-point response and all pertinent correspondence relating to the manuscript. If you do NOT want this file to be published, please inform the editorial office at contact@embomolmed.org.

To submit your manuscript, please follow this link:

https://embomolmed.msubmit.net/cgi-bin/main.plex

When submitting your revised manuscript, please include:

1) a .docx formatted version of the manuscript text (including Figure legends and tables)

2) Separate figure files*

3) supplemental information as Expanded View and/or Appendix. Please carefully check the authors guidelines for formatting Expanded view and Appendix figures and tables at https://www.embopress.org/page/journal/17574684/authorguide#expandedview

4) a letter INCLUDING the reviewer's reports and your detailed responses to their comments (as Word file).

5) The paper explained: EMBO Molecular Medicine articles are accompanied by a summary of the articles to emphasize the major findings in the paper and their medical implications for the non-specialist reader. Please provide a draft summary of your article highlighting
- the medical issue you are addressing,
- the results obtained and
- their clinical impact.
This may be edited to ensure that readers understand the significance and context of the research.
Please refer to any of our published articles for an example.

6) For more information: There is space at the end of each article to list relevant web links for further consultation by our readers. Could you identify some relevant ones and provide such information as well? Some examples are patient associations, relevant databases, OMIM/proteins/genes links, author's websites, etc...

7) Author contributions: the contribution of every author must be detailed in a separate section.

8) EMBO Molecular Medicine now requires a complete author checklist (https://www.embopress.org/page/journal/17574684/authorguide) to be submitted with all revised manuscripts. Please use the checklist as guideline for the sort of information we need WITHIN the manuscript. The checklist should only be filled with page numbers were the information can be found. This is particularly important for animal reporting, antibody dilutions (missing) and exact values and n that should be indicted instead of a range.

9) Every published paper now includes a 'Synopsis' to further enhance discoverability. Synopses are displayed on the journal webpage and are freely accessible to all readers. They include a short stand first (maximum of 300 characters, including space) as well as 2-5 one sentence bullet points that summarise the paper. Please write the bullet points to summarise the key NEW findings. They should be designed to be complementary to the abstract - i.e. not repeat the same text. We encourage inclusion of key acronyms and quantitative information (maximum of 30 words / bullet point). Please use the passive voice. Please attach these in a separate file or send them by email, we will incorporate them accordingly.

You are also welcome to suggest a striking image or visual abstract to illustrate your article. If you do please provide a jpeg file 550 px-wide x 400-px high.

10) A Conflict of Interest statement should be provided in the main text

11) Please note that we now mandate that all corresponding authors list an ORCID digital identifier. This takes <90 seconds to complete. We encourage all authors to supply an ORCID identifier, which will be linked to their name for unambiguous name identification.

Currently, our records indicate that the ORCID for your account is 0000-0002-6250-7902.

Please click the link below to modify this ORCID:
Link Not Available

12) The system will prompt you to fill in your funding and payment information. This will allow Wiley to send you a quote for the article processing charge (APC) in case of acceptance. This quote takes into account any reduction or fee waivers that you may be eligible for. Authors do not need to pay any fees before their manuscript is accepted and transferred to our publisher.

*Additional important information regarding Figures

Each figure should be given in a separate file and should have the following resolution:
Graphs 800-1,200 DPI
Photos 400-800 DPI
Colour (only CMYK) 300-400 DPI"

Figures are not edited by the production team. All lettering should be the same size and style; figure panels should be indicated by capital letters (A, B, C etc). Gridlines are not allowed except for log plots. Figures should be numbered in the order of their appearance in the text with Arabic numerals. Each Figure must have a separate legend and a caption is needed for each panel.

*Additional important information regarding figures and illustrations can be found at https://bit.ly/EMBOPressFigurePreparationGuideline. See also figure legend preparation guidelines: https://www.embopress.org/page/journal/17574684/authorguide#figureformat

The system will prompt you to fill in your funding and payment information. This will allow Wiley to send you a quote for the article processing charge (APC) in case of acceptance. This quote takes into account any reduction or fee waivers that you may be eligible for. Authors do not need to pay any fees before their manuscript is accepted and transferred to our publisher.

***** Reviewer's comments *****

Referee #3 (Remarks for Author):

The authors have adequately answered all our remarks and questions, and adjusted the document accordingly.
We have no further comments.

Referee #4 (Remarks for Author):

Overall, the manuscript is significantly improved. The authors have adequately addressed most of my experimental concerns. I do have some minor edits I would recommend to their written text, which often over-reaches from their actual data.
Experimental concerns:
1) Figure 2E- This only has an n=2 per sample and I am not convinced by either the western provided, or the statistical analysis performed that they are getting correction to an appropriate control here. The DGKk expression is also all over the place. The western blot here is also uncompelling. It is not central to the findings of the paper, so I would remove this (and perhaps 2D) or do it properly with an appropriate negative control (expression of an empty vector or the non deltaN version of DGKk).
Language
2) In the abstract, I would make the following change:
Current abstract ending:
"Here we show that DGKk, when modified as to become FMRP-independent and delivered into the brain of adolescent Fmr1- 42 KO mice using adeno-associated viral vector Rh10, corrects their abnormal cerebral 43 diacylglycerol/phosphatidic acid homeostasis and their main behavioral phenotypes. 44 Altogether our data indicate that DGKk is a key triggering factor of FXS pathomechanism 45 while providing a preclinical proof of concept for FXS gene therapy."

Recommended changes to the abstract:
"Here we show that adeno-associated viral vector delivery of a modified and FMRP-independent form of DGKk corrects abnormal cerebral diacylglycerol/phosphatidic acid homeostasis and FXS relevant behavioral phenotypes in the fmr1 KO mouse. Our data suggests that DGKk is an important factor in FXS pathogenesis and provides preclinical proof of concept that its replacement could be a viable therapeutic strategy in Fragile X Syndrome."

3) Some of the sentence structures are unclear with extraneous information. For example, first sentence of discussion:
"FXS is currently uncured as no disease-modifying treatment could be validated despite several clinical trials with investigational drugs."

Would more accurately and succinctly be written as:

"There are currently no disease modifying treatments for FXS."

4) The authors often refer to the behavioral corrections as corrections of "the main" behavioral findings in the FMR1 KO mouse. It would probably be better to refer to it as correction of "disease relevant behavioral abnormalities in the FMR1 KO mouse". "The main" implies that these are the only disease relevant behavioral phenotypes- which is not true.

5) A small thing, but it is hard to read the paper given that they never indent their paragraphs but also do not include lines between their paragraphs.

Point-by-point response

Referee #3:

The authors have adequately answered all our remarks and questions, and adjusted the document accordingly.
We have no further comments.

Response: We thank the reviewer for this evaluation

Referee #4 (Remarks for Author):

Overall, the manuscript is significantly improved. The authors have adequately addressed most of my experimental concerns. I do have some minor edits I would recommend to their written text, which often over-reaches from their actual data.
Experimental concerns:
1) Figure 2E- This only has an n=2 per sample and I am not convinced by either the western provided, or the statistical analysis performed that they are getting correction to an appropriate control here. The DGKk expression is also all over the place. The western blot here is also uncompelling. It is not central to the findings of the paper, so I would remove this (and perhaps 2D) or do it properly with an appropriate negative control (expression of an empty vector or the non deltaN version of DGKk).
Language
Response: We thank the reviewer for this comment. Because increased-eIF4E phosphorylation status is a well-established marker of FXS cellular condition, we think it was important to provide a demonstration that deltaN version of DGKk had ability to act on this parameter. We have repeated these experiments and added 3 more biological replicates (i.e. primary cultures from distinct embryos) and their WT controls, totaling now n=5. These new data enabled to merged Fig 2D and E in a single figure 2D. Statistical analyses now show a better effect. Unfortunately it was not possible to test non deltaN version of DGKk in this system because DGKk exceeds the encapsidation size limit of the AAV vector.

2) In the abstract, I would make the following change:
Current abstract ending:
"Here we show that DGKk, when modified as to become FMRP-independent and delivered into the brain of adolescent Fmr1- 42 KO mice using adeno-associated viral vector Rh10, corrects their abnormal cerebral 43 diacylglycerol/phosphatidic acid homeostasis and their main behavioral phenotypes. 44 Altogether our data indicate that DGKk is a key triggering factor of FXS pathomechanism 45 while providing a preclinical proof of concept for FXS gene therapy."

Recommended changes to the abstract:
"Here we show that adeno-associated viral vector delivery of a modified and FMRP-independent form of DGKk corrects abnormal cerebral diacylglycerol/phosphatidic acid homeostasis and FXS relevant behavioral phenotypes in the fmr1 KO mouse. Our data suggests that DGKk is an important factor in FXS pathogenesis and provides preclinical proof of concept that its replacement could be a viable therapeutic strategy in Fragile X Syndrome."
Response: We thank the reviewer for this recommendation. The abstract text was modified as recommended.

3) Some of the sentence structures are unclear with extraneous information. For example, first sentence of discussion:
"FXS is currently uncured as no disease-modifying treatment could be validated despite several clinical trials with investigational drugs."

Would more accurately and succinctly be written as:

"There are currently no disease modifying treatments for FXS."
<u>Response:</u> We thank the reviewer for this recommendation. The text was modified as recommended. Several sentences throughout document were edited to improve clarity.

4) The authors often refer to the behavioral corrections as corrections of "the main" behavioral findings in the FMR1 KO mouse. It would probably be better to refer to it as correction of "disease relevant behavioral abnormalities in the FMR1 KO mouse". "The main" implies that these are the only disease relevant behavioral phenotypes- which is not true.
<u>Response:</u> We thank the reviewer for this suggestion. Text was modified as suggested.

5) A small thing, but it is hard to read the paper given that they never indent their paragraphs but also do not include lines between their paragraphs.
<u>Response:</u> We thank the reviewer for this suggestion. Lines were included between the paragraphs.

11th Mar 2022

Dear Dr. MOINE,

We are pleased to inform you that your manuscript is accepted for publication and is now being sent to our publisher to be included in the next available issue of EMBO Molecular Medicine.

We would like to remind you that as part of the EMBO Publications transparent editorial process initiative, EMBO Molecular Medicine will publish a Review Process File online to accompany accepted manuscripts. If you do NOT want the file to be published or would like to exclude figures, please immediately inform the editorial office via e-mail.

Please read below for additional IMPORTANT information regarding your article, its publication and the production process.

Congratulations on your interesting work,
Jingyi

Jingyi Hou
Editor
EMBO Molecular Medicine

Follow us on Twitter @EmboMolMed
Sign up for eTOCs at embopress.org/alertsfeeds

*** *** *** IMPORTANT INFORMATION *** *** ***

SPEED OF PUBLICATION
The journal aims for rapid publication of papers, using the advance online publication "Early View" to expedite the process: A properly copy-edited and formatted version will be published as "Early View" after the proofs have been corrected. Please help the Editors and publisher avoid delays by providing e-mail address(es), telephone and fax numbers at which author(s) can be contacted.

Should you be planning a Press Release on your article, please get in contact with embomolmed@wiley.com as early as possible, in order to coordinate publication and release dates.

LICENSE AND PAYMENT:

All articles published in EMBO Molecular Medicine are fully open access: immediately and freely available to read, download and share.

EMBO Molecular Medicine charges an article processing charge (APC) to cover the publication costs. You, as the corresponding author for this manuscript, should have already received a quote with the article processing fee separately. Please let us know in case this quote has not been received.

Once your article is at Wiley for editorial production you will receive an email from Wiley's Author Services system, which will ask you to log in and will present you with the publication license form for completion. Within the same system the publication fee can be paid by credit card, an invoice, pro forma invoice or purchase order can be requested.

Payment of the publication charge and the signed Open Access Agreement form must be received before the article can be published online.

PROOFS

You will receive the proofs by e-mail approximately 2 weeks after all relevant files have been sent o our Production Office. Please return them within 48 hours and if there should be any problems, please contact the production office at embopressproduction@wiley.com.

Please inform us if there is likely to be any difficulty in reaching you at the above address at that time. Failure to meet our

deadlines may result in a delay of publication.

All further communications concerning your paper proofs should quote reference number EMM-2021-14649-V4 and be directed to the production office at embopressproduction@wiley.com.

Thank you,

Jingyi Hou
Editor
EMBO Molecular Medicine

EMBO Press Author Checklist

Corresponding Author Name: Hervé MOINE	
Journal Submitted to: EMBO Molecular Medicine	
Manuscript Number: EMM-2021-14649-V4	

USEFUL LINKS FOR COMPLETING THIS FORM
The EMBO Journal - Author Guidelines
EMBO Reports - Author Guidelines
Molecular Systems Biology - Author Guidelines
EMBO Molecular Medicine - Author Guidelines

Reporting Checklist for Life Science Articles (updated January 2022)
This checklist is adapted from Materials Design Analysis Reporting (MDAR) Checklist for Authors. MDAR establishes a minimum set of requirements in transparent reporting in the life sciences (see Statement of Task: 10.31222/osf.io/9sm4x). Please follow the journal's guidelines in preparing your manuscript.
Please note that a copy of this checklist will be published alongside your article.

Abridged guidelines for figures
1. Data
The data shown in figures should satisfy the following conditions:
- the data were obtained and processed according to the field's best practice and are presented to reflect the results of the experiments in an accurate and unbiased manner.
- ideally, figure panels should include only measurements that are directly comparable to each other and obtained with the same assay.
- plots include clearly labeled error bars for independent experiments and sample sizes. Unless justified, error bars should not be shown for technical replicates.
- if n<5, the individual data points from each experiment should be plotted. Any statistical test employed should be justified.
- Source Data should be included to report the data underlying figures according to the guidelines set out in the authorship guidelines on Data Presentation.

2. Captions
Each figure caption should contain the following information, for each panel where they are relevant:
- a specification of the experimental system investigated (eg cell line, species name).
- the assay(s) and method(s) used to carry out the reported observations and measurements.
- an explicit mention of the biological and chemical entity(ies) that are being measured.
- an explicit mention of the biological and chemical entity(ies) that are altered/varied/perturbed in a controlled manner.
- the exact sample size (n) for each experimental group/condition, given as a number, not a range;
- a description of the sample collection allowing the reader to understand whether the samples represent technical or biological replicates (including how many animals, litters, cultures, etc.).
- a statement of how many times the experiment shown was independently replicated in the laboratory.
- definitions of statistical methods and measures:
  - common tests, such as t-test (please specify whether paired vs. unpaired), simple χ2 tests, Wilcoxon and Mann-Whitney tests, can be unambiguously identified by name only, but more complex techniques should be described in the methods section;
  - are tests one-sided or two-sided?
  - are there adjustments for multiple comparisons?
  - exact statistical test results, e.g., P values = x but not P values < x;
  - definition of 'center values' as median or average;
  - definition of error bars as s.d. or s.e.m.

Please complete ALL of the questions below.
Select "Not Applicable" only when the requested information is not relevant for your study.

Materials

Newly Created Materials	Information included in the manuscript?	In which section is the information available? (Reagents and Tools Table, Materials and Methods, Figures, Data Availability Section)
New materials and reagents need to be available; do any restrictions apply?	Not Applicable	

Antibodies	Information included in the manuscript?	In which section is the information available? (Reagents and Tools Table, Materials and Methods, Figures, Data Availability Section)
For **antibodies** provide the following information:<br>- Commercial antibodies: RRID (if possible) or supplier name, catalogue number and or/clone number<br>- Non-commercial: RRID or citation	Yes	Appendix table S3

DNA and RNA sequences	Information included in the manuscript?	In which section is the information available? (Reagents and Tools Table, Materials and Methods, Figures, Data Availability Section)
**Short novel DNA or RNA including primers, probes**: provide the sequences.	Yes	Materials and methods

Cell materials	Information included in the manuscript?	In which section is the information available? (Reagents and Tools Table, Materials and Methods, Figures, Data Availability Section)
**Cell lines:** Provide species information, strain. Provide accession number in repository **OR** supplier name, catalog number, clone number, and/**OR** RRID.	Yes	Materials and Methods
**Primary cultures:** Provide species, strain, sex of origin, genetic modification status.	Yes	Materials and Methods
Report if the cell lines were recently **authenticated** (e.g., by STR profiling) and tested for mycoplasma contamination.	Yes	Materials and Methods

Experimental animals	Information included in the manuscript?	In which section is the information available? (Reagents and Tools Table, Materials and Methods, Figures, Data Availability Section)
**Laboratory animals or Model organisms:** Provide species, strain, sex, age, genetic modification status. Provide accession number in repository **OR** supplier name, catalog number, clone number, **OR** RRID.	Yes	Materials and Methods
**Animal observed in or captured from the field:** Provide species, sex, and age where possible.	Not Applicable	
Please detail **housing and husbandry conditions**.	Yes	Materials and Methods

Plants and microbes	Information included in the manuscript?	In which section is the information available? (Reagents and Tools Table, Materials and Methods, Figures, Data Availability Section)
**Plants:** provide species and strain, ecotype and cultivar where relevant, unique accession number if available, and source (including location for collected wild specimens).	Not Applicable	
**Microbes:** provide species and strain, unique accession number if available, and source.	Not Applicable	

Human research participants	Information included in the manuscript?	In which section is the information available? (Reagents and Tools Table, Materials and Methods, Figures, Data Availability Section)
If collected and within the bounds of privacy constraints report on age, sex and gender or ethnicity for all study participants.	Not Applicable	

Core facilities	Information included in the manuscript?	In which section is the information available? (Reagents and Tools Table, Materials and Methods, Figures, Data Availability Section)
If your work benefited from core facilities, was their service mentioned in the acknowledgments section?	Yes	Materials and Methods

Design

Study protocol	Information included in the manuscript?	In which section is the information available? (Reagents and Tools Table, Materials and Methods, Figures, Data Availability Section)
If study protocol has been **pre-registered, provide DOI in the manuscript**. For clinical trials, provide the trial registration number **OR** cite DOI.	Not Applicable	
Report the **clinical trial registration number** (at ClinicalTrials.gov or equivalent), where applicable.	Not Applicable	

Laboratory protocol	Information included in the manuscript?	In which section is the information available? (Reagents and Tools Table, Materials and Methods, Figures, Data Availability Section)
Provide DOI OR other citation details if **external detailed step-by-step protocols** are available.	Not Applicable	

Experimental study design and statistics	Information included in the manuscript?	In which section is the information available? (Reagents and Tools Table, Materials and Methods, Figures, Data Availability Section)

Include a statement about **sample size** estimate even if no statistical methods were used.	Yes	Materials and Methods and Figure legends
Were any steps taken to minimize the effects of subjective bias when allocating animals/samples to treatment (e.g. **randomization procedure**)? If yes, have they been described?	Yes	Animals/samples were randomly allocated to treatment conditions, but no specific procedure was used.
Include a statement about **blinding** even if no blinding was done.	Yes	Behavioral studies were conducted under experimenter-independent conditions (actimetry, three chamber test, elevated plus maze, novel object recognition) and by experimenters naive to expected effect of genotype and treatment (nesting, grooming, digging).
Describe **inclusion/exclusion criteria** if samples or animals were excluded from the analysis. Were the criteria pre-established?<br><br>If sample or data points were omitted from analysis, report if this was due to attrition or intentional exclusion and provide justification.	Yes	No animal or sample was a priori excluded from the analyses. Where indicated, outlyers were removed from analyses using ROUT method with Q=1% with GraphPad Prism 9 software.
For every figure, are **statistical tests** justified as appropriate? Do the data meet the assumptions of the tests (e.g., normal distribution)? Describe any methods used to assess it. Is there an estimate of variation within each group of data? Is the variance similar between the groups that are being statistically compared?	Yes	Quantitative data were analyzed using single or repeated measures analysis of variance (ANOVA) and Student Newman Keuls test. For the comparison with chance, one group t-test was used. Qualitative parameters (nesting) were analyzed using χ2 test. The level of significance was set at $p < 0.05$.

Sample definition and in-laboratory replication	Information included in the manuscript?	In which section is the information available?<br>(Reagents and Tools Table, Materials and Methods, Figures, Data Availability Section)
In the figure legends: state number of times the experiment was **replicated** in laboratory.	Yes	Figures
In the figure legends: define whether data describe **technical or biological replicates**.	Yes	Figures

Ethics

Ethics	Information included in the manuscript?	In which section is the information available?<br>(Reagents and Tools Table, Materials and Methods, Figures, Data Availability Section)
Studies involving **human participants**: State details of **authority granting ethics approval** (IRB or equivalent committee(s), provide reference number for approval.	Not Applicable	
Studies involving **human participants**: Include a statement confirming that **informed consent** was obtained from all subjects and that the experiments conformed to the principles set out in the WMA Declaration of Helsinki and the Department of Health and Human Services Belmont Report.	Not Applicable	
Studies involving **human participants:** For publication of **patient photos**, include a statement confirming that consent to publish was obtained.	Not Applicable	
Studies involving experimental **animals**: State details of **authority granting ethics approval** (IRB or equivalent committee(s), provide reference number for approval. Include a statement of compliance with ethical regulations.	Yes	Materials and Methods
Studies involving **specimen and field samples:** State if relevant **permits** obtained, provide details of authority approving study; if none were required, explain why.	Not Applicable	

Dual Use Research of Concern (DURC)	Information included in the manuscript?	In which section is the information available?<br>(Reagents and Tools Table, Materials and Methods, Figures, Data Availability Section)
Could your study fall under dual use research restrictions? Please check biosecurity documents and list of **select agents and toxins** (CDC): https://www.selectagents.gov/sat/list.htm	Not Applicable	
If you used a select agent, is the security level of the lab appropriate and reported in the manuscript?	Not Applicable	
If a study is subject to dual use research of concern regulations, is the name of the **authority granting approval and reference number** for the regulatory approval provided in the manuscript?	Not Applicable	

Reporting

The MDAR framework recommends adoption of discipline-specific guidelines, established and endorsed through community initiatives. Journals have their own policy about requiring specific guidelines and recommendations to complement MDAR.

Adherence to community standards	Information included in the manuscript?	In which section is the information available?<br>(Reagents and Tools Table, Materials and Methods, Figures, Data Availability Section)
State if relevant guidelines or checklists (e.g., **ICMJE, MIBBI, ARRIVE, PRISMA**) have been followed or provided.	Yes	Materials and Methods
For **tumor marker prognostic studies,** we recommend that you follow the **REMARK** reporting guidelines (see link list at top right). See author guidelines, under 'Reporting Guidelines'. Please confirm you have followed these guidelines.	Not Applicable	
For **phase II and III randomized controlled trials**, please refer to the **CONSORT** flow diagram (see link list at top right) and submit the CONSORT checklist (see link list at top right) with your submission. See author guidelines, under 'Reporting Guidelines'. Please confirm you have submitted this list.	Not Applicable	

Data Availability

Data availability	Information included in the manuscript?	In which section is the information available?<br>(Reagents and Tools Table, Materials and Methods, Figures, Data Availability Section)
Have **primary datasets** been deposited according to the journal's guidelines (see 'Data Deposition' section) and the respective accession numbers provided in the Data Availability Section?	Not Applicable	
Were **human clinical and genomic datasets** deposited in a public access-controlled repository in accordance to ethical obligations to the patients and to the applicable consent agreement?	Not Applicable	
Are **computational models** that are central and integral to a study available without restrictions in a machine-readable form? Were the relevant accession numbers or links provided?	Not Applicable	
If publicly available data were reused, provide the respective **data citations in the reference list**.	Not Applicable	
